# The role of photobiomodulation in the functional recovery of proximal humerus fractures: a randomized controlled clinical protocol

**DOI:** 10.1371/journal.pone.0321746

**Published:** 2025-04-29

**Authors:** Luiz Claudio de Freitas, Do Sung Kim, Daniel Santana da Costa, Henrique Pellacani Fernandes Soutello, Thiago Roncoletta Salata, Luis Fumio Sato, Nilton Iuichi Takahashi, Valenthin de Souza Gomes, Priscila Terumi Kondo, Gustavo Guedes Lomonaco, Bruno Ricardo Trigo, Cinthya Cosme Gutierrez Duran, Sandra Kalil Bussadori, Lara Jansiski Motta, Raquel Agnelli Mesquita-Ferrari, Anna Carolina Ratto Tempestini Horliana, Kristianne Porta Santos Fernandes

**Affiliations:** 1 Postgraduate Program in Biophotonics-Medicine, Universidade Nove de Julho (UNINOVE), São Paulo, Brazil; 2 Clínica de Ortopedia e Traumatologia do Hospital Dr. Alípio Correa Netto (HMACN), Secretaria Municipal de Saúde, São Paulo, Brazil; 3 Postgraduate Program in Rehabilitation Sciences, Universidade Nove de Julho (UNINOVE), São Paulo, Brazil; Massachusetts General Hospital, UNITED STATES OF AMERICA

## Abstract

Pain and joint stiffness contribute to functional limitation in the postoperative period following proximal humeral fractures (PHF). Photobiomodulation (PBM) has demonstrated positive outcomes in fracture repair, analgesia, and functional improvement, as evidenced by randomized controlled trials (RCTs) and experimental animal studies. Clinical studies have shown PBM’s efficacy in reducing pain and improving functional outcomes, while preclinical studies have demonstrated enhanced bone regeneration through PBM application. This clinical study is a randomized, double-blind, controlled trial to investigate the effects of PBM on the shoulder functional recovery after proximal humerus fractures. A total of forty-two participants, aged 18–65 years of both genders, will be randomly divided into two groups: the Control group (receiving physiotherapy combined with simulated PBM) and the PBM group (receiving physiotherapy combined with active PBM). The PBM application (10 minutes) will be performed daily by the participants at home, using a device equipped with 318 light-emitting diodes (LEDs), consisting of 159 LEDs at 660 nm (28.5 mW; 12 J/cm²; 17 J per LED) and 159 LEDs at 850 nm (23 mW; 10 J/cm²; 14 J per LED). The PBM sessions, along with physiotherapy sessions (30 minutes, twice weekly), will be conducted over a 12-week period. Participants will be blinded to their group allocation and will be assessed by a single evaluator at 24 hours, 1 week, 2 weeks, 4 weeks, 8 weeks, and 12 weeks post-surgery. The evaluator will also be blinded to the participants’ group assignments. The primary outcome will be shoulder functional recovery after proximal humerus fractures, assessed using the Quick-DASH scale at all experimental time points. Secondary outcomes will include range of motion (measured with a digital goniometer), quality of life (evaluated using the SF-6D questionnaire), pain on pressure and the incidence of adverse effects, all assessed at each time point. Spontaneous pain, nocturnal pain and analgesic use will be evaluated over a 12-week period. Fracture consolidation will be assessed through radiography at weeks 4, 8, and 12. Muscle strength will be measured through dumbbell lifting at weeks 8 and 12. If the data are normally distributed, ANOVA will be used, and results will be presented as means ± standard deviation (SD). If the data are not normally distributed, they will be presented as medians and interquartile ranges, with comparisons made using non-parametric tests. A p-value of less than 0.05 will be considered statistically significant.

## Introduction

The incidence of proximal humeral fractures (PHF) accounts for 5–10% of all bone fractures, varying according to geographic location and the time period of data collection [1,2,3]. The occurrence of PHF increases with age, being the third most common fracture among individuals over 65 years old [2–4]. Most PHFs result from low-energy trauma, primarily falls from standing height or direct trauma to the lateral aspect of the shoulder, with women being more affected due to higher life expectancy and osteoporosis [3,5]. In individuals under 55 years of age, PHFs typically result from high-energy trauma, and their distribution is not significantly influenced by sex [3].

The treatment for PHFs depends on the fracture’s characteristics and the patient’s overall health [6,7]. Typically, non-displaced or minimally displaced and stable PHFs are eligible for conservative treatment, involving the use of a sling for one or more weeks followed by rehabilitation [6,8]. Conversely, displaced, unstable, open fractures, or those associated with vascular injury are generally treated surgically [9–12].

Surgical treatment involves various stabilization techniques, such as fixation with pins, rods, plates, or arthroplasty. Recent systematic reviews have compared the outcomes of these techniques in the progression of patients with PHFs, finding no statistical differences among them or between these techniques and conservative treatment [12,13].

The rehabilitation protocols following PHF are also not well defined in the literature [14]. The interventions used vary widely and may include thermal modalities, manual therapy, kinesitherapy, application of dynamic orthoses, therapeutic guidance and education, and electrophysical resources such as photobiomodulation, which involves the use of light sources (laser and LED) to stimulate healing, relieve pain, and reduce inflammation [15,16].

A systematic review with meta-analysis on the use of photobiomodulation (PBM) in the treatment of limb fractures demonstrated a clinically and statistically significant difference in favor of PBM in improving the function of the fractured limb and reducing pain, although it did not show differences in radiographic healing of the fracture [17]. Other reviews, based on studies with diverse methodologies, have indicated that PBM may accelerate bone regeneration, while also highlighting the need for standardization of treatment parameters and protocols [18–21]. Subsequent clinical studies have highlighted the adjunctive role of PBM combined with physiotherapy in fracture recovery and demonstrated significant improvements in bone healing with PBM [22,23]. Additionally, a cost-effectiveness study suggests that PBM may be a viable adjunct therapy in fracture treatment [24].

The dosimetric parameters of PBM used in the treatment of fractures involve a wavelength of 500–1000 nm, radiant power of 5–100 mW, and radiant exposure of 0.5 to 30 J/cm² [21]. Chang et al. (2014) utilized a laser device with an average power of 60 mW, peak power of 8 W, and a frequency of 10 Hz, delivering a radiant exposure of 9.7 J/cm² for a duration of 600 seconds, resulting in a total energy output of 36 J at each site to treat wrist fractures [25]. Nesioonpour et al. (2014) treated tibial fractures by employing a combination of an 808 nm laser (300 mW in continuous mode, delivering 6 J/cm² for 20 seconds, resulting in 6 J per point) and a 650 nm laser (output power of 100 mW in continuous mode, providing 3 J/cm² for 30 seconds, yielding 3 J per point).Each tibial fracture was irradiated from four sides. The same equipment was used for the irradiation of muscles and surgical wounds, applying an irradiation time of 10 seconds per point at 6–8 points [26]. Similarly, PBM was employed to treat distal radius fractures in two studies utilizing a pulsed laser with a wavelength of 904 nm (peak power of 25 W, mean output of 60 mW, pulse frequency of 60 kHz). In the first study, the treatment protocol consisted of administering 3 J on the dorsal aspect of the wrist and 1.2 J at three points on the palmar aspect of the wrist, delivered over nine sessions within three weeks [27]. The second study likewise examined the application of PBM for distal radius fractures, employing three targeted irradiations (1.2 J per point) at the fracture site on both sides of the arm. This treatment protocol was delivered over a total of nine sessions [23].

In the case of proximal humerus fractures (PHF), in addition to the importance of identifying optimal dosimetric parameters for PBM application, there is also a recognized need to standardize the outcomes to be evaluated [28,29] to establish a gold standard treatment. Most cases involve a prolonged recovery period accompanied by pain and functional limitations. The objective of this study is to evaluate the effects of PBM, using a home-use LED device, on shoulder functional recovery after proximal humerus fractures. This will be assessed through a controlled, randomized, and double-blind clinical trial.

## Materials and methods

This study hypothesizes that photobiomodulation (PBM) improves functional recovery following proximal humerus fractures. It is a randomized, double-blind, controlled trial with two parallel groups: a PBM group receiving active PBM and a Control group receiving simulated PBM, in a 1:1 randomization ratio. Blinding will be maintained for participants, evaluators, and therapists. Assessments will be conducted at 24 hours and at 1, 2, 4, 8, and 12 weeks post-surgery, evaluating functional performance (Quick-DASH), pain, range of motion, quality of life, muscle strength, and fracture consolidation. The primary outcome is shoulder functional recovery, assessed using the Quick-DASH questionnaire.

**Fig 1:**
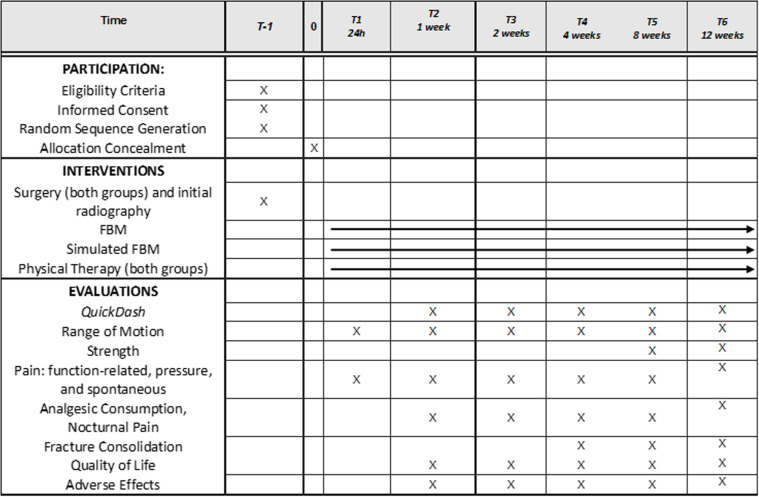
SPIRIT Flowchart. No legend.

This is a parallel, controlled, randomized, double-blind clinical trial will be conducted at Dr. Alípio Correa Netto Hospital, São Paulo, Brazil. The study design follows the international recommendations for randomized clinical trials as outlined (Fig 1 and S1 Appendix) in the SPIRIT protocol (Standard Protocol Items: Recommendations for Interventional Trials). This protocol (S4 and S5 Appendixs) was approved (S2 and S3 Appendixs) on May 23^rd^, 2023, by the Ethics Committee (process 6.075.552, CAAE 69030123.7.0000.5511). The project was registered at ClinicalTrials.gov (NCT06113614, first posted on 2023-11-02) (S6 Appendix). The recruitment began on April 30^th^, 2024, and is expected to be completed by December 30^th^, 2025. The estimated study completion date is April 30^th^, 2026.

In the emergency department, the examining researchers will request imaging tests according to hospital standards (shoulder X-rays in anteroposterior, scapular lateral, and axillary views, as well as a CT scan when indicated). Preoperative tests will also be requested, including a complete blood count, coagulation profile, blood typing, serum levels of sodium, potassium, urea, and creatinine, blood glucose, electrocardiogram, and chest X-rays in posteroanterior and lateral views. An echocardiogram will be ordered when clinically indicated. Evaluations from other specialties may be requested when relevant. The potential inclusion of participants in the study will be determined only after clinical and radiographic assessment.

### Ethical consideration

Participants will be informed about the study’s objectives and methods through a verbal explanation and written information on the procedures to be used, and they will be invited to participate by the principal investigator. Those who agree will sign the Informed Consent Form (ICF).

### Study population

Patients with isolated closed proximal humerus fractures with displacement, scheduled for surgery, willing to participate in the study and eligible according to the following criteria:

### Inclusion criteria

- Participants aged 18 to 65 years of both genders, representing a broad demographic of individuals commonly affected by PHF. These participants, with PHF classified as:
- Neer group III
- Neer group IV
- Neer group V
- AO/OTA subgroups A2 and A3
- AO/OTA group B
- AO/OTA group C (only in patients younger than 55 years).

### Exclusion criteria

- Individuals with prior injuries or sequelae in the shoulder or scapular girdle, or motor deficits due to central or peripheral neurological injuries.
- Patients with pathological fractures (caused by metabolic, infectious, tumoral, or osteoporotic diseases).
- Participants with postoperative infection or implant loosening.
- Those with ipsilateral fractures in other areas of the limb.
- Patients with neurovascular injuries causing sensory deficits at the injury site.
- Individuals with local or systemic conditions contraindicating surgical intervention or complicating the postoperative period.
- Participants with a history of photosensitivity.
- Individuals with neurological or psychiatric disorders.
- Those with proliferative or infectious skin lesions in the shoulder area where LED light will be applied.
- Participants who used anti-inflammatory medications within 5 days prior to the trauma.
- Pregnancy.
- Participants who experience surgical complications, such as neurological or vascular injuries or fracture line extension during surgery, as they will not meet the desired progression criteria.

### Calculation of sample size

The number of participants in each experimental group was determined based on the variability of one study [20], which assessed the effects of laser PBM on functional recovery in wrist and hand fractures using the Quick DASH questionnaire. Using the power/sample size calculator developed by the University of British Columbia (https://www.stat.ubc.ca/~rollin/stats/ssize/n2.html) and considering a significance level of 0.05, a power of 90%, and an anticipated 15% loss rate for the specified outcome, the required sample size will be 42 individuals, with 21 per group.

Anatomical and functional differences between wrist and shoulder fractures may introduce variability in the sample size calculation. However, due to the limited published data on PBM effects in proximal humerus fractures, the sample size was based on the most relevant study assessing functional recovery using the Quick-DASH questionnaire in a similar musculoskeletal context. An interim analysis will be performed to reassess the sample size based on actual study data, ensuring adequate statistical power. Sensitivity analyses will also be conducted to validate the findings and assess result robustness.

### Strategies for achieving adequate participant enrolment

To reach the required number of participants, the attending physicians at the hospital will be contacted to notify the principal investigator upon the arrival of patients with isolated, closed proximal humerus fractures with displacement and surgical indication.

### Experimental groups

**Fig 2:**
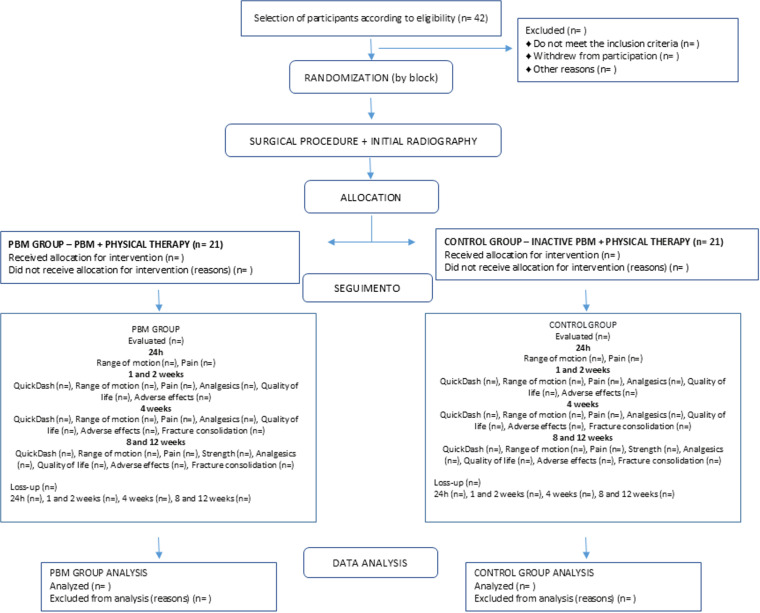
Study Flowchart. No legend.

Participants will be divided into two groups (Fig 2):

PBM Group: Participants will receive active PBM applied at home, in conjunction with physiotherapy.

Control Group: Participants will receive placebo PBM applied at home, also in conjunction with physiotherapy.

### Randomization

The sequence generation and envelope preparation will be conducted by a researcher not involved in the study. To randomly allocate participants into the two experimental groups, a random sequence generator program (https://www.randomizer.org/tutorial/) will be used, selecting the option for block randomization with blocks of 6 participants, thus forming 7 blocks. Opaque envelopes will be labeled with each number, and inside each envelope, a sheet containing the corresponding experimental group information, based on the generated sequence, will be placed. The envelopes will be sealed and kept in numerical order within a secure plastic box in a safe location.

### Blinding

A researcher not involved in any evaluations will prepare the randomization and the envelopes to ensure allocation concealment. Once participants are enrolled in the study, another researcher, also not involved in any evaluations, will retrieve the envelope containing the allocation information and provide the appropriate PBM device (active or placebo) along with instructions for its use at home. Four evaluators, orthopedic surgeons (blinded to each participant’s experimental group), will conduct all assessments from the postoperative period through outpatient follow-up. A physical therapist (blinded to each participant’s experimental group) will oversee the participants throughout all therapy sessions. Three resident physicians (blinded to each participant’s experimental group) will be responsible for daily contact with participants to monitor PBM device usage, provide guidance, answer questions, and address concerns. Participants will remain unaware of the group to which they are allocated, as they will receive shoulder pad for home use. The shoulder pads in the control group will simulate treatment by emitting the same sound and incorporating a guide light in the activation plug. Furthermore, participants will be informed that the shoulder pad is equipped with both visible (red) and invisible (infrared) LEDs, and they will be advised that they might not see the light emitted by the device during treatment.

### Examiner calibration and training

The principal investigator (orthopedic surgeon) will collect all study outcomes. He will conduct range of motion assessments on 10 volunteers, outside of the study sample, across two assessment periods with a 10-day interval. Agreement between these measurements will be evaluated using the Intraclass Correlation Coefficient [30].

### Medication prescription

During the hospitalization period, intravenous analgesia will be administered to ensure pain control: dipyrone 1g every 6 hours and tramadol hydrochloride 100 mg every 8 hours for the first 24 hours. If the patient requires rescue analgesia, tramadol hydrochloride administration may be adjusted to every 6 hours. Morphine sulfate will be available as a second-line rescue option at a dose of 2 mg IV, up to every 8 hours. Patients will be reassessed to adjust the standard analgesic prescription, transitioning to an “as-needed” opioid prescription whenever feasible. All analgesic consumption will be documented. Corticosteroids and anti-inflammatory drugs will not be prescribed.

At hospital discharge, participants will be prescribed oral dipyrone 1g every 6 hours for 5 days and tramadol hydrochloride 50 mg orally every 8 hours, to be used only as needed, for up to 5 days. Participants will be instructed to use tramadol hydrochloride only if pain persists despite dipyrone use or in cases of moderate pain, classified as 4–7 on the pain scale. Participants reporting the use of non-steroidal anti-inflammatory drugs (NSAIDs) or corticosteroids will undergo a sensitivity analysis to assess the impact of their use on study outcomes. However, continuous or excessive use of NSAIDs will be considered a withdrawal criterion due to potential interference with the study’s primary outcomes.

### Surgical procedure

Four orthopedic surgeons with experience in proximal humerus fractures (PHF) will be responsible for all surgical procedures.

All participants will undergo general anesthesia combined with a brachial plexus block, and the surgical procedure will follow the standard protocol of the hospital’s orthopedic department.

Fractures will be approached through a deltopectoral incision. Careful dissection will be performed to avoid excessive exposure of the fragments and to preserve vascularization. The fragments will be anatomically reduced [6] through indirect maneuvers and temporarily stabilized with Kirschner wires (Biomecânica, Jaú, SP, Brazil). Final stabilization will be achieved using a 3.5 mm anatomically contoured locking plate for the proximal humerus (GM Reis, Campinas, SP, Brazil), which is considered the gold standard for PHF stabilization [6,23]. The plate will be positioned 1 cm below the superior edge of the greater tuberosity and 1 cm lateral to the long head of the biceps tendon. Reduction and positioning of the plate and screws will be assessed intraoperatively using fluoroscopy.

### Physiotherapy

All participants will receive treatment in 30-minute sessions, twice a week, for 12 weeks, following the standardized physiotherapy protocol [9].

The sessions will include:

- Sling immobilization, pendulum exercises, assisted passive movements, no external rotation (weeks 1–3).
- Assisted active flexion and abduction, gradual loading after six weeks (weeks 3–9).
- Isotonic, eccentric, and concentric exercises; passive stretching if contracture persists after Week 12 (after week 9).

The hospital protocols related to surgical procedures, analgesia and physiotherapy remained unchanged.

### Photobiomodulation (PBM) Application

**Fig 3:**
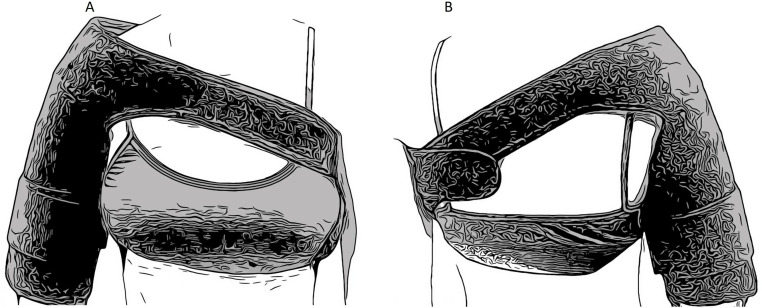
PBM device. PBM device: A: front view, B: back view.

PBM will be applied using LED devices in the form of a shoulder pad (Fig 3). Applications will occur daily throughout the experimental period, beginning 24 hours after the surgical procedure. Each pad will be for individual use, and all participants will be instructed to disinfect its plastic surface with 70% alcohol before and after each PBM application. Specifically, during the period when the dressing covers the suture area (for a maximum of 15 days), participants will be guided to wash their hands, remove the dressing, clean the suture area, and apply the shoulder pad. Treatments will be conducted at the participant’s home, with instructions provided on how to wear and operate the device at the time of hospital discharge, along with written guidance. Each application session will last 10 minutes.

**Fig 4:**
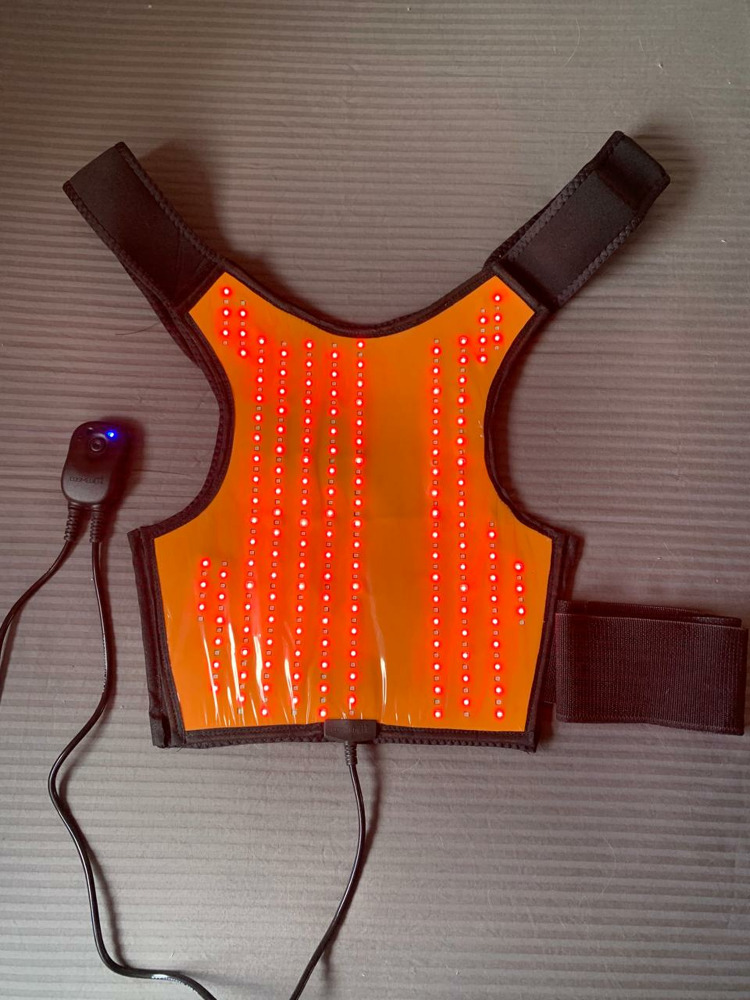
PBM device. Figure 4: Internal view of the activated shoulder pad highlighting the visible red LEDs and the invisible infrared LEDs.

The shoulder pad by Cosmedical (Mauá, SP, Brazil) contains 159 red LEDs and 159 infrared LEDs arranged alternately. The device has been designed to ensure adequate contact with the shoulder region, targeting the proximal humerus. It is equipped with Velcro fasteners to allow for precise adjustment to various body types. The coverage extending to the upper arm is intended to deliver consistent irradiation to the surrounding tissues that support the functional recovery of the shoulder joint. The area corresponding to the fracture fixation plate, with an additional 2 cm margin around the entire extent, will not be irradiated, as no LEDs will be placed in this area (Fig 4).

Initial tests utilizing a surface thermometer were conducted to assess skin temperature both before and immediately after the 10-minute application. The results indicated an increase of 0.5 °C, confirming the device’s safety and its effectiveness in preventing excessive warming during treatment. Participants in the control group will receive an identical device. Only the activation light and sound will be triggered upon pressing the button, with the internal LEDs remaining off. The dosimetric parameters of the LED device (Table 1) allow for a combination of higher and lower energy wavelengths (red and infrared, respectively) with varying depths of penetration (infrared deeper than red), delivering energy per point and radiant exposures within the limits reported in the literature, as mentioned before [21–28].

**Table 1:** Dosimetric parameters.

Parameter	PBM	PBM
	red	infrared
Central wavelength (nm)	**660**	**850**
Spectral width (FWHM) (nm)	**19**	**30**
Mode of operation	continuous	continuous
Average radiant power per LED (mW)	28.5	23
Quantity of LEDs	159	159
Radiant energy per LED (J)	17	14
Polarization	random	random
Diameter of each LED opening [mm]	3	3
Irradiance at the opening per LED mW/cm²]	20.4	16.5
Beam profile	multimode	multimode
Exposure time (s)	600	600
Radiant exposure per LED [J/cm²]	12	10
Device area	900 cm^2^	
Mode of application	in contact with the skin	
Session frequency	Once a day for 90 days	

nm (nanometers), FWHM (Full Width at Half Maximum), mW (milliwatts), cm² (square centimeters), mW/cm² (milliwatts per square centimeter), s (seconds), J (Joules), J/cm² (Joules per square centimeter), cm (centimeters), h (hours).

Considering the total area of the device (900 cm²) and the cumulative energy dispensed by all LEDs, the final radiant exposure is 5.46 J/cm². When considering the total power output of all LEDs relative to the device area, the total irradiance is 9.1 mW/cm².

The radiant power of each LED was measured using a power meter with a pyroelectric sensor (Coherent, model Field Max2, Wilsonville, OR, United States).

### Daily follow-up

Three physicians will establish daily contact with participants through a messaging app to monitor PBM device usage, provide guidance, answer questions, and collect data on spontaneous and nocturnal pain, as well as analgesic consumption. The first contact will be conducted via video call to reinforce proper equipment positioning and usage instructions. During the first five days, participants will be specifically questioned about the administration of 1g oral dipyrone every 6 hours and 50mg oral tramadol hydrochloride every 8 hours, with tramadol being used only as needed. In addition to monitoring device usage and outcomes, these daily messages are intended to improve participant adherence to the protocol. Telephone calls will be used only for the initial contact or when necessary.

### Criteria for discontinuing or modifying allocated interventions

Therapy will be discontinued if bleeding, sepsis, urticaria, or any discomfort occurs that justifies stopping either the active or placebo PBM application. In cases of treatment discontinuation for any reason, absence from in-person evaluations, or lack of response during scheduled virtual contacts, data will be collected up to the point of interruption and included in the statistical analysis using a mixed-effects regression model.

### Outcome assessment in brief

#### a) Primary outcome.

The primary outcome of the study is shoulder functional recovery, assessed using the Quick-DASH (Disabilities of the Arm, Shoulder, and Hand) questionnaire at all experimental time points. The Quick-DASH score ranges from 0 to 100, with higher scores indicating greater disability and lower scores indicating better functional recovery [31,32].

#### b) Secondary outcomes.

Range of Motion (ROM): Measured using a digital goniometer in degrees of flexion, extension, abduction, adduction, internal, and external rotation [33].

Quality of Life: Assessed using the SF-6 questionnaire, with scores ranging from 0 (worst health) to 1 (best health state) [34–36]

Pain: Evaluated through the Visual Analog Scale (VAS), ranging from 0 (no pain) to 10 (worst pain imaginable) for spontaneous and nocturnal pain [37].

Analgesic Consumption: Recorded based on patient self-reports and verified through daily monitoring.

Muscle Strength: Assessed by progressive dumbbell lifting, with incremental weight measurements to determine the participant’s maximum capacity [5].

Fracture Consolidation: Evaluated through radiographic assessments at 4, 8, and 12 weeks, based on callus formation criteria [33,38]

#### c) Safety outcomes.

Occurrence of adverse effects, including skin irritation, discomfort, or any potential complications related to PBM application. These will be monitored through daily follow-ups and reported based on participant feedback.

### Outcome assessment in details

#### a) Questionnaire QuickDASH (Disabilities of the Arm, Shoulder and Hand).

Shoulder function will be assessed using the QuickDASH questionnaire [24], which has been validated for use in Brazil [32]. This questionnaire will be administered at 1, 2, 4, 8, and 12 weeks following the surgical procedure.

#### b)Range of motion.

Shoulder range of motion will be evaluated with the participant in a standing position. The maximum tolerated position in extension, flexion, abduction, adduction, lateral rotation, and medial rotation will be recorded by the evaluator. Rotations will be assessed at 0° of abduction and flexion [33,39]. All measurements will be conducted using a digital goniometer (Kaptron 360, Shenzhen, Dongguan, China). Range of motion will be assessed 1 day post-surgery and subsequently at 1 and 2 weeks using passive movement. At 4, 8, and 12 weeks, active movement will be evaluated.

#### c)Muscle strength.

Muscle strength will be evaluated at 8 and 12 weeks through progressive dumbbell holding (RLM, Maringá, Paraná, Brazil) with elbow flexion in both arms. Progressive holding will start with the unaffected arm, beginning at 500 grams and increasing up to the maximum weight the participant can hold, not exceeding 5 kg [5]. Each dumbbell weight will be lifted only once per arm.

#### d)Pain assessment.

Spontaneous pain intensity and pain related to the injured limb will be assessed using the visual analog pain scale [37,39]. Pressure pain at the fracture site will be evaluated with a digital algometer (MED DOR, Governador Valadares, MG, Brazil). Pain data will be collected 1 day post-surgery and subsequently at 1, 2, 4, 8, and 12 weeks. The occurrence of nocturnal pain will be inquired about during daily monitoring of PBM usage.

#### e)Analgesic consumption.

The type and dosage of analgesics consumed will be recorded during the daily monitoring of PBM usage.

#### f) Quality of life.

Quality of life will be assessed using the SF-6 instrument from 2002 [34,35], in the version adapted for use in Brazil [36], at 1, 2, 4, 8, and 12 weeks.

#### g) Occurrence of adverse effects.

The occurrence of adverse effects will be inquired about during the daily monitoring of PBM usage.

#### h) Fracture consolidation.

Shoulder X-rays in anteroposterior, scapular lateral, and axillary views will be taken at 4, 8, and 12 weeks to evaluate bone consolidation, defined as the presence of callus formation in 3 out of 4 bone cortices uniting the main fracture fragments, compared to the immediate postoperative X-ray [33,38]**.**

### Criteria of success

**Criteria of Success:**

1. Primary Outcome: Significant improvement in shoulder functional recovery in the PBM group vs. control.
2. Secondary Outcomes: Improved range of motion, quality of life, pain reduction, muscle strength, and faster fracture consolidation in the PBM group, with no severe adverse effects.
3. Benefit-Risk Ratio: Greater clinical benefits with PBM and physiotherapy combined with minimal risks.

### Criteria of compliance

Treatment Adherence: Daily PBM sessions and twice-weekly physiotherapy for 12 weeks.

Device Usage: Correct and consistent use of the PBM device as instructed.

Follow-Up: Completion of all evaluations at scheduled time points.

Data Reporting: Accurate reporting of pain, analgesic use, and adverse effects.

Compliance will be assessed based on session completion, follow-up attendance, and accurate data reporting.

### Data management plans

Initial descriptive analyses will encompass all study variables, reporting quantitative data as means and standard deviations, and qualitative data as frequencies and percentages. For normally distributed data, a mixed-design ANOVA will be applied, with Group as a between-subjects factor and Time as a within-subjects factor, accounting for the repeated measures nature of the data. Bonferroni correction will be applied to control Type I error inflation across multiple outcome measures. Additionally, the false discovery rate (FDR) method will be considered where appropriate to further ensure statistical findings’ validity. Results will be presented as means ± standard deviation (SD). Medians and interquartile ranges will be reported for non-normally distributed data, and comparisons will be made using the Kruskal-Wallis and Friedman tests for time-related analyses. Categorical variables will be assessed using the chi-square test, Fisher’s exact test, or likelihood ratio tests as appropriate.

Covariate adjustment will be performed using multiple regression models to account for potential confounding factors, such as variability in surgical skill, postoperative care adherence, and comorbidities. Subgroup analyses will be pre-specified based on baseline characteristics, including age, gender, trauma energy, and dominant limb involvement. Statistical tests for subgroup comparisons will incorporate interaction terms within regression models to avoid post hoc bias and enhance the interpretation of results. A significance level of 5% or the corresponding p-value will be utilized for all statistical tests. All analyses will be performed using SAS for Windows statistical software, version 9.1. Intention-to-treat analysis will be utilized in conjunction with multiple imputation methods if data loss exceeds 10%, as this level of missing data necessitates a more robust approach to uphold the validity of the results. To account for the potential non-randomness of missing data, auxiliary variables predictive of missingness will be incorporated into the imputation model. Additionally, sensitivity analyses will be conducted to assess the robustness of the results under different missing data assumptions, including missing at random (MAR) and missing not at random (MNAR) scenarios. No interim analyses or stopping guidelines are planned, as the interventions are regarded as safe. Personal information of potential and enrolled participants will be collected, shared, and maintained with strict confidentiality protocols among researchers before, during, and after the trial.

### Plans for communicating important protocol modifications

Changes to eligibility criteria, outcomes, and analyses will be communicated to relevant parties, including investigators, trial participants, trial registries, and journals, to uphold transparency and comply with ethical and regulatory standards.

## Discussion

There is substantial evidence demonstrating PBM’s capacity to control pain and assist in tissue repair. However, in the treatment of fractures, there remains a need for randomized controlled trials (RCTs) with high methodological rigor to establish the optimal parameters and outcomes of PBM [14–17,33,34]. In the case of proximal humerus fractures (PHF), in addition to the importance of identifying optimal dosimetric parameters for PBM application, there is a recognized need to standardize the outcomes to be evaluated [28,29] in order to establish a gold standard treatment, as the majority of cases involve prolonged recovery periods accompanied by pain and functional limitations. This study will evaluate the effects of PBM (using a home-use LED device with parameters supported by the literature) primarily on functional recovery in the postoperative period of surgically treated proximal humerus fractures using ORIF (Open Reduction and Internal Fixation) with an angular-stable plate, through a controlled, randomized, and double-blind clinical trial.

This study explores the innovative use of photobiomodulation (PBM) for fracture recovery and pain management. The implementation of a safe and user-friendly home device enhances patient adherence to treatment protocols while reducing the need for clinic visits. The robust randomized, controlled, and double-blind study design ensures the collection of high-quality data. A comprehensive evaluation will focus on functional recovery, pain levels, range of motion, and fracture consolidation. However, a limitation of this study is the exclusive inclusion of surgically treated cases, which may restrict the generalizability of findings to non-surgical or conservatively treated populations. Future studies should consider expanding eligibility criteria to include patients undergoing conservative treatment to evaluate the broader applicability of PBM in different clinical scenarios.

### Monitoring

Interim analyses are not planned, as no serious adverse events related to PBM use are expected, given that no adverse effects have been reported in the literature. Adverse events related to surgery will be managed at the hospital according to the institution’s standard protocol. However, all adverse events will be recorded.

## Supporting information

S1 Appendix Spirit. (DOCX)

S2 Appendix IRB aproval in English. (PDF)

S3 Appendix IRB aproval in original language. (PDF)

S4 Appendix Study protocol approved by IRB in English. (DOCX)

S5 Appendix Study protocol approved by IRB in original language. (DOCX)

S6 Appendix Clinicaltrials. (PDF)
