# Supplementary material for: The role of photobiomodulation in the functional recovery of proximal humerus fractures: a randomized controlled clinical protocol
Source: PLoS One. 2025 Apr 29;20(4):e0321746. doi: 10.1371/journal.pone.0321746 (PMC12040229; doi:10.1371/journal.pone.0321746)
Supplement: S2 Appendix — (PDF) [file pone.0321746.s002.pdf]

CONSOLIDATED IRB OPINION

RESEARCH PROJECT DATA

Title of the Study: EFEITOS DA FOTOBIOMODULAÇÃO NA RECUPERAÇÃO FUNCIONAL DE FRATURAS DO UMERO PROXIMAL: ESTUDO CLÍNICO CONTROLADO RANDOMIZADO DUPLO CEGO.

Researcher: LUIZ CLAUDIO DE FREITAS

Thematic area:

Versio:2

CAAE: 69030123.7.0000.5511

Proponent Institution: ASSOCIACAO EDUCACIONAL NOVE DE JULHO

Main sponsor: Self-financing

RESEARCH PROJECT DATA

IRB Opinion Number:6.075.552

Presentation of project:

Among the various complications in the postoperative progression of proximal humerus fractures (PHF) are pain and joint stiffness, leading to significant functional limitations in the affected limb. Physical therapy is the standard treatment for both surgical and non-surgical cases. Studies have shown positive effects of photobiomodulation (PBM) in fracture repair and regeneration, as well as in analgesia and functional improvement. However, they suggest the need for standardization and additional evidence.

This randomized double-blind controlled clinical study aims to evaluate the effects of PBM on the functional recovery of participants with surgically treated PHF using specialized locked plates. The 42 participants will be randomized (1:1) into two groups: Control (standard physiotherapy treatment with simulated PBM) and PBM (standard physiotherapy treatment with active PBM). PBM will be applied by the participant at home daily for 10 minutes using a device with 318 LEDs—159 LEDs of 660 nm (28.5 mW; 12 J/cm²; 17 J per LED) and 159 LEDs of 850 nm (23 mW; 10 J/cm²; 14 J per LED).

PBM and physical therapy sessions (30 minutes, twice a week) will be performed for 12 weeks. Participants will be blinded to their allocation and assessed at 24 hours, 1, 2, 4, 8, and 12 weeks post-surgery by four evaluators, also blinded to each participant's allocation.

Primary Outcome: Functional recovery of the shoulder, evaluated at all experimental periods using the Quick-DASH functional scale.

Secondary Outcomes: These include assessments of shoulder range of motion with a digital goniometer, quality of life using the SF-6 questionnaire, and the occurrence of adverse effects across all experimental periods. Additional evaluations include spontaneous pain, pressure pain (dolorimeter), nighttime pain, and analgesic use at 1, 2, 4, 8, and 12 weeks; fracture consolidation at 4, 8, and 12 weeks through radiographic examinations; and muscle strength assessed via progressive dumbbell support at 8 and 12 weeks. Each participant will also be followed daily by phone contact.

Collected data will be stored, organized in a repository, and analyzed with appropriate statistical tests. A 5% significance level will be adopted for all tests.

Primary Objective: To evaluate functional recovery after proximal humerus fractures treated with open reduction and internal fixation (ORIF), stabilized with fixed-angle plates, and treated with physical therapy and photobiomodulation using the Brazilian version of the Quick DASH (Disabilities of the Arm, Shoulder, and Hand) questionnaire.

Secondary Objectives: To assess the effects of PBM applied after proximal humerus fractures treated with ORIF, stabilized with fixed-angle plates, and treated with physical therapy on:

- Shoulder range of motion (ROM) over time and in comparison with the unaffected limb
- Muscle strength over time and compared to the unaffected limb
- Intensity of spontaneous and functional pain
- Occurrence of nighttime pain
- Pressure pain at the fracture site
- Analgesic consumption

- Fracture consolidation
- Quality of life
- Incidence of adverse events
- Direct and indirect costs related to research procedures for subsequent cost-effectiveness analysis of the intervention.

Risk and Benefit Assessment:

Risks: During medical consultations, there may be brief pain during movement, strength, and light pressure tests at the site, which are necessary for evaluating recovery progress. Regarding LED use, no risks have been described to date. Participants should avoid looking directly at the lights, as with any light source. The shoulder device will be connected to an outlet and, like any electrical appliance, should not be exposed to water. Medications for pain control (dipyrone and tramadol) are standard for all shoulder surgery patients. Dipyrone may cause skin allergies like redness and itching, or, more rarely, serious reactions like shortness of breath. Tramadol may cause dizziness, headache, constipation, and nausea. Rare side effects of tramadol include shortness of breath and palpitations. As participants will be contacted via cell phone, there is a risk of device theft or hacking, which could expose their contact details.

Benefits: There will be no direct benefits to research participants.

Risk-Benefit Assessment: Second version of the project.

Comments and Considerations:

- Initial descriptive analyses will be conducted considering all measured variables

- both quantitative (mean and standard deviation) and qualitative (frequencies and percentages). Subsequent normality analyses will determine appropriate statistical tests for each dataset, with subgroup analyses (osteoporosis, osteopenia, high/low-intensity trauma, smoking, other comorbidities) conducted where necessary. A 5% significance level or corresponding p-value will be adopted for all tests, using SAS for Windows, version 9.1.
- Primary study outcome: QuickDASH Questionnaire.

Considerations on Mandatory Presentation Terms:
Mandatory Documentation:

- Dated Cover Page, signed by the director with the director's stamp: Presented and fulfilled. OK
- Research Project: Presented and fulfilled. OK
- Timeline: Presented and fulfilled. OK
- Informed Consent Form (ICF): Presented and fulfilled. OK

- O TCLE deve ser redigido para o participante (direcionada ao leigo). A linguagem utilizada deve ser diferente da linguagem científica dos projetos. Apresentado e atendido OK.

- Por se tratar de um projeto que terá uma etapa em ambiente virtual (acompanhamento telefônico diário), sugere-se adequar o TCLE de acordo com o OFÍCIO CIRCULAR No 2/2021/CONEP/SECNS/MS disponível em: http://conselho.saude.gov.br/images/Oficio_Circular_2_24fev2021.pdf

Sugere-se uma atenção especial aos itens riscos e garantia do sigilo. Apresentado e atendido OK.

- No item 4, adicionar a possibilidade da prescrição da medicação, deixando claro ser um procedimento independente do projeto de pesquisa. Apresentado e atendido OK.

- Ainda no item 4 não está claro se o uso da ombreira será no domicílio no participante e quem irá manusear a ombreira. Será o próprio participante da pesquisa? No projeto está descrito que "As aplicações ocorrerão no próprio domicílio do participante que será orientado para vestir e manusear o equipamento no momento da alta hospitalar. Cada aplicação terá duração de 10 minutos." Favor deixar claro este ponto. No caso de ser o participante de pesquisa que irá manusear o aparelho, há algum risco quanto ao uso errado? Os participantes receberão óculos de proteção? Especificar os riscos do aparelho. Apresentado e atendido OK.

- No projeto de pesquisa, nos apêndices 9 e 10, está descrito que o participante de pesquisa

will take the shoulder device home. However, this procedure is not clearly specified in the ICF. Presented and fulfilled. OK.

Although it is clear that there will be two groups (in item 4 of the ICF), it is not clear that the participant could be assigned to either of these groups. Appropriate. OK.

In item 5, mention the risks related to medication prescription, LED usage, and handling of the shoulder device. Also, mention the risks related to participating in research with a virtual component. Presented and fulfilled. OK.

In item 7, it is stated that "The participant will have the benefit of daily monitoring by the medical team for 3 months"; however, item 11 states that "evaluations will be conducted during routine consultations." Clarify whether this monitoring is a direct benefit. If it is not, it is suggested to add that "There will be no direct benefits for research participants." Presented and fulfilled. OK.

Item 11, "Reimbursement Guarantee," needs to be rewritten. "There is no provision for reimbursing participant expenses and those of their companions, such as transport and food, as the evaluations will occur during routine consultations. Res. No 466/12 – Item II.21." The cited resolution states that even as part of routine consultations, the research participant is entitled to reimbursement upon request. Please refer to the Research Participants' Rights Guide. Presented and fulfilled. OK.

Include the address of the research site in item 12 of the ICF and clarify if any research stages will occur at the participant's home. Presented and fulfilled. OK.

In Appendix 9, it is stated that the participant should "use [the device] for 12 weeks," and later, "at the end of 8 weeks, the device should be returned to team members, and you will sign a return agreement." Please clarify both in the project and the ICF the usage period of the shoulder device. Presented and fulfilled. OK.

Also in Appendix 9, it is specified that the participant should "reimburse the equipment's value in cases of loss, misplacement, or damage caused by improper use or negligence." Please consult the Research Participants' Rights Guide and rewrite the text. Presented and fulfilled. OK.

Consent letter from the co-participating institution - Presented and fulfilled. OK.

Conclusions or Pending Issues and List of Inadequacies:

The reported pending issues have been addressed, and the Project has been approved.

Final Considerations at the CEP's Discretion:

The researcher must report to the institution conducting the study (which authorized the research) to initiate data collection. The research participant (or their representative) and the responsible researcher must initial each page of the Informed Consent Form (ICF) and sign the last page of the form, as per Circular Letter No. 003/2011 from CONEP/CNS.

We emphasize that the researcher must carry out the research according to the approved protocol. Any modifications or amendments to the protocol must be submitted to the CEP clearly and concisely, identifying the part of the protocol to be modified along with its justification. This modification will require ethical approval from the CEP before implementation. All altered documents must be presented objectively with justification for re-evaluation to facilitate the review process.

The researcher is responsible for archiving and safeguarding the research data for 5 years, including individual records and all other documents recommended by the CEP (CNS Resolution 466/12, item X.1.2.f).

According to CNS Resolution 466/12, X.3.b), the researcher must submit semi-annual reports to this CEP/SMS. The final report must be sent via the Plataforma Brasil, under the Notification icon. A digital copy of the completed project must be sent to the institution that authorized the study, by mail, email, or delivered in person, once the study is concluded.

Continuing of report: 6.075.552

This opinion was prepared based on the documents listed below:

Document Type	File	Posting Date	Author	Status
Basic Project Information	PB_INFORMAÇÕES_BÁSICAS_DO_PROJETO_2278654.pdf	12/05/2023 15:22:59		Accepted
Detailed Project / Investigator Brochure	PROJETO REVISADO PARA CEP.PDF	12/05/2023 15:22:06	Kristianne Porta Santos Fernandes	Accepted
Investigator	PROJETO_REVISADO_PARA_CEP.pdf	12/05/2023 15:22:06		
ICF / Assent Forms / Justification for Absence	TCLE_REVISADO.pdf	12/05/2023 15:10:59	Kristianne Porta Santos Fernandes	Accepted
Cover Page	folhaDeRosto.pdf	12/05/2023 15:03:21	Kristianne Porta Santos Fernandes	Accepted

Opinion Status:

Approved

Requires CONEP Review: No

SAO PAULO, 23 de Maio de 2023

Signed by:

Maria Aparecida Dalboni

(Coordinator)

Adress: VERGUEIRO nº 235/249
Neiborhood: LIBERDADE **ZIP CODE** : 01.504-001
UF: SP **Municipality:** SAO PAULO
Phone: (11)3385-9010 **E-mail:** comitedeetica@uninove.br
