# Supplementary material for: The role of photobiomodulation in the functional recovery of proximal humerus fractures: a randomized controlled clinical protocol
Source: PLoS One. 2025 Apr 29;20(4):e0321746. doi: 10.1371/journal.pone.0321746 (PMC12040229; doi:10.1371/journal.pone.0321746)
Supplement: S3 Appendix — (PDF) [file pone.0321746.s003.pdf]

PARECER CONSUBSTANCIADO DO CEP

DADOS DO PROJETO DE PESQUISA

Título da Pesquisa: EFEITOS DA FOTOBIOMODULAÇÃO NA RECUPERAÇÃO FUNCIONAL DE FRATURAS DO UMERO PROXIMAL: ESTUDO CLÍNICO CONTROLADO RANDOMIZADO DUPLO CEGO.

Pesquisador: LUIZ CLAUDIO DE FREITAS

Área Temática:

Versão: 2

CAAE: 69030123.7.0000.5511

Instituição Proponente: ASSOCIACAO EDUCACIONAL NOVE DE JULHO

Patrocinador Principal: Financiamento Próprio

DADOS DO PARECER

Número do Parecer: 6.075.552

Apresentação do Projeto:

Dentre as várias complicações da evolução pós-operatória das fraturas do úmero proximal (FUP) estão a dor e a rigidez articular, gerando limitação
funcional importante no membro afetado. A fisioterapia é o tratamento padrão tanto para os casos cirúrgicos quanto os não cirúrgicos. Estudos tem
demonstrado efeitos positivos da fotobiomodulação (FBM) na reparação e regeneração das fraturas, bem como na analgesia e melhora funcional.
No entanto, sugerem padronização e evidências adicionais. O presente estudo clínico duplo cego controlado randomizado terá como objetivo avaliar
os efeitos da FBM na recuperação funcional de participantes com FUP tratadas cirurgicamente com placas bloqueadas especiais. Os 42
participantes serão randomizados (1:1) em 2 grupos, sendo grupo Controle (tratamento padronizado de fisioterapia associada a FBM simulada) e
grupo FBM (tratamento padronizado de fisioterapia associada a FBM ativa). A FBM será aplicada pelo próprio participante em seu domicílio todos os
dias, por 10 minutos, com uso de um dispositivo contendo 318 LEDs light emitting diodes, sendo 159 LEDs de 660 nm (28,5 mW; 12 J/cm2; 17 J por
LED) e 159 LEDs de 850 nm (23 mW; 10 J/cm2; 14 J por LED). As sessões de FBM e as de

fisioterapia (30 minutos, 2 vezes por semana) serão
realizadas por 12 semanas. Os participantes não terão conhecimento de sua alocação e serão avaliados em 24h, 1, 2, 4, 8 e 12 semanas após o
procedimento cirúrgico por 4 examinadores também cegos em relação a alocação de cada participante. O desfecho principal, avaliado em todos os
períodos experimentais, será a recuperação da função do ombro utilizando a escala funcional Quick-DASH. Os desfechos secundários serão as
avaliações de amplitude dos movimentos do ombro com goniômetro digital, qualidade de vida com uso do questionário SF-6 e ocorrência de efeitos
adversos em todos os períodos experimentais. Já a dor espontânea, à pressão (dolorímetro), noturna e o uso de analgésicos serão avaliados em 1,
2, 4, 8 e 12 semanas; a consolidação das fraturas em 4, 8 e 12 semanas por meio de exames radiográficos; e a força muscular por sustentação
progressiva de halteres em 8 e 12 semanas. Além das avaliações pontuais, cada participante será acompanhado diariamente por meio de contato
telefônico. Os dados coletados serão armazenados, organizados em repositório e serão aplicados os testes estatísticos apropriados para cada
análise específica. Em todos os testes, será adotado o nível de significância de 5%.

Objetivo da Pesquisa:

Objetivo Primário:
O desfecho principal do estudo será a avaliação da recuperação funcional após fraturas de úmero proximal, procedidas com RAFI, estabilizadas com
placa de ângulo fixo e tratadas com fisioterapia e fotobiomodulação, por meio da versão brasileira do questionário Quick DASH (Disabilities of the
Arm, Shoulder and Hand).
Objetivo Secundário:
Os desfechos secundários serão avaliar os efeitos da FBM aplicada após fraturas de úmero proximal, procedidas com RAFI, estabilizadas com placa
de ângulo fixo e tratadas com fisioterapia sobre:• A amplitude dos movimentos (ADM) do ombro avaliada de maneira temporal e em comparação
com o membro não afetado• A força muscular mensurada de maneira temporal e em comparação com o membro não afetado• A intensidade
da dor espontânea e durante a função do membro• A ocorrência de dor noturna• A dor à pressão

no local da fratura• O consumo de
analgésicos • A consolidação da fratura• A qualidade de vida• A incidência de eventos adversos • Os custos diretos e indiretos relacionados aos
procedimentos realizados no período da pesquisa para posterior cálculo da relação custo efetividade da intervenção.

Avaliação dos Riscos e Benefícios:

Riscos:
Durante as consultas médicas, pode ocorrer sensação de dor nos testes de movimentação, força e na leve pressão no local, porém a duração da dor
é bem rápida. Esses testes não trazem nenhum risco e são necessários para que o médico avalie como está caminhando sua recuperação. Quanto
ao uso do LED, não temos até o momento deste trabalho descrição de riscos relacionados ao seu uso. Não é aconselhável ficar olhando fixamente
para as luzes da ombreira, assim como não é aconselhável olhar fixamente para qualquer lâmpada. A ombreira funcionará ligada a uma tomada e
como todo aparelho elétrico, não deve ser molhada. Os medicamentos que serão usados para controle da dor (dipirona e tramadol) são receitados
para todos os pacientes que passam por cirurgia de ombro, mesmo os que não participam da pesquisa. A dipirona pode causar reações alérgicas na
pele como vermelhidão e coceira e mais raramente reações como falta de ar que são mais graves. A dipirona também pode causar alterações nas
células do sangue e de defesa do organismo como também sangramento no estômago. O tramadol pode causar sintomas como tontura, dor de
cabeça, intestino preso e náusea. Outros efeitos mais raros do tramadol são falta de ar e palpitação. Como faremos contato com você usando um
telefone celular, existe o risco do aparelho ser roubado ou invadido virtualmente e seu contato ficar exposto para pessoa que não participe do
estudo.
Benefícios:
Não haverá benefícios diretos ao participante da pesquisa.

Comentários e Considerações sobre a Pesquisa:

Projeto de segunda versão.
Serão realizadas análises descritivas iniciais considerando todas as variáveis medidas no estudo,

tanto quantitativas (média e desvio padrão) quanto qualitativas (frequências e porcentagens). Posteriormente serão realizadas as análises de normalidade para determinar os testes estatísticos apropriados para cada conjunto de dados e aplicados os testes estatísticos apropriados para cada análise específica. Serão realizadas também análises por subgrupos (presença de osteoporose, osteopenia, traumas de alta e baixa intensidade, tabagismo, outras comorbidades). Em todos os testes, será adotado o nível de significância de 5% de probabilidade ou o p-valor correspondente. Todas as análises serão realizadas utilizando o programa estatístico SAS for Windows, versão 9.1. Desfecho primário do estudo: Questionário QuickDASH (Disabilities of the Arm, Shoulder and Hand).

Considerações sobre os Termos de apresentação obrigatória:

Documentação obrigatória:

Folha de rosto datada, assinada pelo diretor com carimbo do diretor - Apresentada e atendida. OK

Projeto de pesquisa - Apresentada e atendida. OK

Cronograma - Apresentado e atendido. OK

TCLE - Apresentado e atendido. OK

- O TCLE deve ser redigido para o participante (direcionada ao leigo). A linguagem utilizada deve ser diferente da linguagem científica dos projetos. Apresentado e atendido OK.
- Por se tratar de um projeto que terá uma etapa em ambiente virtual (acompanhamento telefônico diário), sugere-se adequar o TCLE de acordo com o OFÍCIO CIRCULAR No 2/2021/CONEP/SECNS/MS disponível em: http://conselho.saude.gov.br/images/Oficio_Circular_2_24fev2021.pdf

Sugere-se uma atenção especial aos itens riscos e garantia do sigilo. Apresentado e atendido OK.

- No item 4, adicionar a possibilidade da prescrição da medicação, deixando claro ser um procedimento independente do projeto de pesquisa. Apresentado e atendido OK.
- Ainda no item 4 não está claro se o uso da ombreira será no domicílio no participante e quem irá manusear a ombreira. Será o próprio participante da pesquisa? No projeto está descrito que "As aplicações ocorrerão no próprio domicílio do participante que será orientado para vestir e manusear o equipamento no momento da alta hospitalar. Cada aplicação terá duração de 10 minutos." Favor deixar claro este ponto. No caso de ser o participante de pesquisa que irá manusear o aparelho, há algum risco quanto ao uso errado? Os participantes receberão óculos de proteção? Especificar os riscos do aparelho. Apresentado e atendido OK.
- No projeto de pesquisa, nos apêndices 9 e 10, está descrito que o participante de pesquisa

levará o a ombreira para casa. No entanto esse procedimento não está claro no TCLE. Apresentado e atendido Ok.

- Apesar de estar claro que haverá dois grupos (no item 4 do TCLE), não está claro que o participante poderá ser sorteado para qualquer um desses dois grupos. Adequado Ok.
- No item 5, mencionar os riscos relativos a prescrição das medicações. Mencionar os riscos relativos ao uso do LED e ao manuseio da ombreira. Mencionar os riscos relativos a participação de pesquisa com etapa em ambiente virtual. Apresentado e atendido Ok.
- No item 7, está descrito que "O participante terá o benefício do acompanhamento diário da equipe médica durante 3 meses" no entanto no item 11 está descrito que "as avaliações serão realizadas durante as consultas de rotina". Deixar claro se o acompanhamento é benefício direto ou não. Caso não seja, sugere-se adicionar que "Não haverá benefícios diretos ao participante de pesquisa". Apresentado e atendido Ok.
- O item 11 "Garantia de ressarcimento" tem que ser reescrito. "Não há previsão de ressarcimento dos custos de despesas do participante e seus acompanhantes como transporte e alimentação, uma vez que as avaliações serão realizadas durante as consultas de rotina. Res. No 466/12 – Item II.21". A resolução citada afirma que mesmo fazendo parte das consultas de rotina, o participante de pesquisa tem direito a ressarcimento, caso solicite. Favor consultar a Cartilha dos Direitos dos Participantes de Pesquisa. Apresentado e atendido Ok.
- Colocar o endereço do local da pesquisa no item 12 do TCLE bem como deixar claro se haverá etapas da pesquisa no domicílio do participante. Apresentado e atendido Ok
- No apêndice 9 está descrito que o participante deverá: "- Usar durante 12 semanas." e mais a frente "- No final de 8 semanas, o dispositivo deverá ser devolvido aos membros da equipe e você assinará o termo de devolução." Favor deixar claro tanto no projeto quanto no TCLE o período de uso da ombreira. Apresentado e atendido OK.
- Ainda no apêndice 9 está descrito que o participante deverá: "- Restituir o valor do equipamento em casos de perda, extravio ou danos causados por uso inadequado ou descuido". Favor consultar a Cartilha dos Direitos dos Participantes de Pesquisa e reescrever o texto. Apresentado e atendido. OK
- Carta de anuência da instituição coparticipante - Apresentada e atendida. Ok

Conclusões ou Pendências e Lista de Inadequações:

As pendências relatadas foram atendidas e o Projeto aprovado.

Endereço: VERGUEIRO nº 235/249 12º andar sala 02
Bairro: LIBERDADE **CEP:** 01.504-001
UF: SP **Município:** SAO PAULO
Telefone: (11)3385-9010 **E-mail:** comitedeetica@uninove.br

Considerações Finais a critério do CEP:

O pesquisador deverá se apresentar na instituição de realização da pesquisa (que autorizou a realização do estudo) para início da coleta dos dados.

O participante da pesquisa (ou seu representante) e o pesquisador responsável deverão rubricar todas as folhas do Termo de Consentimento Livre e Esclarecido - TCLE apondo sua assinatura na última página do referido Termo, conforme Carta Circular no 003/2011 da CONEP/CNS.

Salientamos que o pesquisador deve desenvolver a pesquisa conforme delineada no protocolo aprovado.

Eventuais modificações ou emendas ao protocolo devem ser apresentadas ao CEP de forma clara e sucinta, identificando a parte do protocolo a ser modificada e suas justificativas. Lembramos que esta modificação necessitará de aprovação ética do CEP antes de ser implementada. De forma objetiva com justificativa para nova apreciação, os documentos alterados devem ser evidenciados para facilitar a nova análise.

Ao pesquisador cabe manter em arquivo, sob sua guarda, por 5 anos, os dados da pesquisa, contendo fichas individuais e todos os demais documentos recomendados pelo CEP (Res. CNS 466/12 item X1. 2. f).

De acordo com a Res. CNS 466/12, X.3.b), o pesquisador deve apresentar a este CEP/SMS os relatórios semestrais. O relatório final deverá ser enviado através da Plataforma Brasil, ícone Notificação. Uma cópia digital do projeto finalizado deverá ser enviada à instância que autorizou a realização do estudo, via correio, e-mail ou entregue pessoalmente, logo que o mesmo estiver concluído.

Este parecer foi elaborado baseado nos documentos abaixo relacionados:

Tipo Documento	Arquivo	Postagem	Autor	Situação
Informações Básicas do Projeto	PB_INFORMAÇÕES_BÁSICAS_DO_PROJETO_2124002.pdf	12/05/2023 15:22:59		Aceito
Projeto Detalhado / Brochura	PROJETO_REVISADO_PARA_CEP.pdf	12/05/2023 15:22:06	Kristianne Porta Santos Fernandes	Aceito

Endereço: VERGUEIRO nº 235/249 12º andar sala 02
Bairro: LIBERDADE **CEP:** 01.504-001
UF: SP **Município:** SAO PAULO
Telefone: (11)3385-9010 **E-mail:** comitedeetica@uninove.br

Investigador	PROJETO_REVISADO_PARA_CEP.pdf	12/05/2023 15:22:06	Kristianne Porta Santos Fernandes	Aceito
Declaração de concordância	CARTA_ANUENCIA_HOSPITAL.pdf	12/05/2023 15:11:36	Kristianne Porta Santos Fernandes	Aceito
TCLE / Termos de Assentimento / Justificativa de Ausência	TCLE_REVISADO.pdf	12/05/2023 15:10:59	Kristianne Porta Santos Fernandes	Aceito
Folha de Rosto	folhaDeRosto.pdf	12/05/2023 15:03:21	Kristianne Porta Santos Fernandes	Aceito

Situação do Parecer:
Aprovado

Necessita Apreciação da CONEP:
Não

SAO PAULO, 23 de Maio de 2023

Assinado por:
Maria Aparecida Dalboni
(Coordenador(a))
