# Supplementary material for: The role of photobiomodulation in the functional recovery of proximal humerus fractures: a randomized controlled clinical protocol
Source: PLoS One. 2025 Apr 29;20(4):e0321746. doi: 10.1371/journal.pone.0321746 (PMC12040229; doi:10.1371/journal.pone.0321746)
Supplement: S4 Appendix — (DOCX) [file pone.0321746.s004.docx]

**POSTGRADUATE PROGRAM IN BIOPHOTONICS APPLIED TO HEALTH SCIENCES**

**LUIZ CLÁUDIO DE FREITAS**

**PHOTOBIOMODULATION EFFECTS IN THE POSTOPERATIVE PERIOD OF PROXIMAL HUMERAL FRACTURES: A DOUBLE-BLIND RANDOMIZED CONTROLLED CLINICAL STUDY**

**São Paulo, SP**

**2023**

**UNIVERSIDADE NOVE DE JULHO**

**POSTGRADUATE PROGRAM IN BIOPHOTONICS APPLIED TO HEALTH SCIENCES**

**LUIZ CLÁUDIO DE FREITAS**

**PHOTOBIOMODULATION EFFECTS IN THE POSTOPERATIVE CARE OF PROXIMAL HUMERAL FRACTURES: A DOUBLE-BLIND RANDOMIZED CONTROLLED CLINICAL STUDY**

Research project submitted to the

Research Ethics Committee of Universidade Nove de Julho

**São Paulo, SP**

**2023**

**ABSTRACT**

Among the various complications of the postoperative evolution of fractures of the proximal humerus (FUP) are pain and joint stiffness, generating significant functional limitation in the affected limb. Physical therapy is the standard treatment for both surgical and non-surgical cases. Studies have shown positive effects of photobiomodulation (PBM) on repair and regeneration of fractures, as well as on analgesia and functional improvement. However, they suggest standardization and additional evidence. This randomized controlled double-blind clinical study will aim to evaluate the effects of PBM on the functional recovery of participants with FUP surgically treated with special locking plates. The 42 participants will be randomized (1:1) into 2 groups, the Control group (standardized physiotherapy treatment associated with simulated PBM) and the PBM group (standardized physiotherapy treatment associated with active PBM). The PBM will be applied by the participant at home every day, for 10 minutes, using a device containing 318 LEDs light emitting diodes, 159 LEDs of 660 nm (28.5 mW; 12 J/cm2; 17 J per LED) and 159 850 nm LEDs (23 mW; 10 J/cm2; 14 J per LED). The PBM and physiotherapy sessions (30 minutes, 2 times a week) will be held for 12 weeks. Participants will not be aware of their allocation and will be assessed at 24h, 1, 2, 4, 8 and 12 weeks after the surgical procedure by 4 examiners who are also blinded to each participant's allocation. The main outcome, evaluated in all experimental periods, will be the recovery of shoulder function using the Quick-DASH functional scale. Secondary outcomes will be assessments of range of motion of the shoulder using a digital goniometer, quality of life using the SF-6 questionnaire and the occurrence of adverse effects in all experimental periods. Spontaneous pain, pressure pain (dolorimeter), nocturnal pain and the use of analgesics will be evaluated at 1, 2, 4, 8 and 12 weeks; consolidation of fractures at 4, 8 and 12 weeks through radiographic examinations; and muscle strength by progressive weight bearing at 8 and 12 weeks. In addition to punctual evaluations, each participant will be monitored daily through telephone contact. The collected data will be stored, organized in a repository and the appropriate statistical tests will be applied for each specific analysis. In all tests, a significance level of 5% will be adopted.

Keywords: Photobiomodulation, Humeral Fracture, Quick-DASH, Quality of life, Phototherapy

**LIST OF TABLES**

Table 1. Randomized Controlled Clinical Trials on PBM in Bone Fractures................... 32
Table 2. Dosimetric Parameters of the PBM Device...................................................... 32
Table 3. Treatment Costs.............................................................................................. 38

LIST OF ABBREVIATIONS AND ACRONYMS

ADM ....... Range of Motion

AMP ....... Adenosine Monophosphate

AO ....... Arbeitsgemeinschaft fur Osteosynthesefragen (German for the Association for the Study of Internal Fixation)

ASES ....... American Shoulder and Elbow Surgeon’s Score

ATP ....... Adenosine Triphosphate

CEP ....... Research Ethics Committee

CONSORT ....... Consolidated Standards of Reporting Trials

DASH ....... Disabilities of the Arm, Shoulder and Hand

ECR ....... Randomized Clinical Trial

EQ-5D ....... Euroqol-5Dimension

EROS ....... Reactive Oxygen Species

EVA ....... Visual Analog Scale

PBM ....... Photobiomodulation

FUP ....... Proximal Humerus Fractures

HMACN ....... Municipal Hospital Alípio Correa Netto

ICC ....... Intraclass Correlation Coefficient

LEDs ....... Light Emitting Diodes

OTA ....... Orthopaedic Trauma Association

PRO ....... Patient Reported Outcomes

ProFHER ....... Proximal Fracture of Humerus: Evaluation by Randomisation

RAFI ....... Open Reduction and Internal Fixation

SF36 ....... 36-Item Short-Form Health Survey

SF6D ....... Short-Form 6 dimension

SPIRIT ....... Standard Protocol Items: Recommendations for Interventional Trials

TCLE ....... Free and Informed Consent Form

TRP ....... Transient Receptor Potential

**1. CONTEXTUALIZATION**

Fractures of the proximal humerus (FUP) are those that occur in the surgical neck (the narrowing just below the greater and lesser tuberosities of the humerus) or in regions proximal to it. Their incidence varies depending on the geographic region and the date of data collection, but in general, they represent about 5 to 10% of all bone fractures in the body (Court-Brown & Cesar 2006, Relvas Silva et al 2021, Iglesias-Rodríguez et al 2021). The incidence of FUP tends to increase with age and is identified as the third most frequent fracture in the population over 65 years of age, depending on the state of health, level of activity, and care received (Roux et al 2012, Relvas Silva et al 2021, Iglesias-Rodríguez et al 2021).

In older adults, most FUP cases are related to low-energy traumas, primarily due to falls from their own height or direct trauma to the lateral aspect of the shoulder. In these cases, women are more affected due to longer life expectancy and osteoporosis (Barbosa et al 2008, Iglesias-Rodríguez et al 2021). In individuals under 55 years, traumas leading to FUP tend to be of higher energy, and their distribution is not as affected by gender (Iglesias-Rodríguez et al 2021).

**Classification**

The primary classifications used for proximal humeral fractures are Neer and AO/OTA. Neer's classification (1970) defined the four fracture fragments: head, greater and lesser tuberosities, and diaphysis. Fractures are considered displaced when the fragments are displaced at least 1 cm or angulated at a minimum of 45°. Thus, fractures can be classified as non-displaced and displaced/dislocated in 2, 3, or 4 parts, as follows (Carrerra et al 2012, Petros et al 2019):

Group I: Minimally displaced fracture (displacement less than 1 cm or angulation less than 45°).

Group II: Displaced fracture (more than 1 cm of displacement or more than 45° of angulation). Fragment = anatomical neck of the proximal humerus.

Group III: Displaced fracture (more than 1 cm of displacement or more than 45° of angulation). Fragment = surgical neck of the proximal humerus.

Group IV: Fracture with displacement of the greater tuberosity of the humerus (more than 1 cm of displacement or more than 45° of angulation).

2 parts: (no displacement of the surgical neck);

3 parts: (displacement of the surgical neck);

4 parts: (displacement of the surgical neck and lesser tuberosity).

Group V: Fracture with a displacement of the lesser tuberosity (more than 1 cm of displacement or more than 45° of angulation).

2 parts: (no displacement of the surgical neck);

3 parts: (displacement of the surgical neck);

4 parts: (displacement of the surgical neck and lesser tuberosity).

Group VI: Fracture associated with glenohumeral dislocation and can also be subdivided into two, three, or four parts.

The AO/OTA classification is an alphanumeric structure based on imaging exams (X-rays, tomography, and, if necessary, magnetic resonance imaging). Each bone is defined by a number, and for the humerus, it is number 1. The location of the fracture within the bone is also defined by numbers: proximal segment (1), diaphysis (2), and distal segment (3), following Heim's square system. Regarding the type of fracture, it is classified as extra-articular unifocal or two parts (A), extra-articular bifocal or three parts (B), and articular or four parts (C) as follows (Carrera et al 2012):

**11 - Proximal humerus region**

A - Extra-articular unifocal

A1: Tuberosities

A1.1 - Greater tuberosity

A1.2 - Lesser tuberosity

A2: Surgical neck

A2.1 - Simple

A2.2 - Wedge

A2.3 - Multifragmented

A3 - Vertical

**11B - Extra-articular, bifocal**

B1: Surgical neck

B1.1 - With greater tuberosity

B1.2 - With lesser tuberosity

**11C - Articular or four parts**

C1: Anatomic neck

C1.1 - Impacted in valgus

n - Greater tuberosity

o - Lesser tuberosity

p - Both

C1.2 - Isolated neck

**Treatment**

The treatment of proximal humeral fractures (PHF) remains controversial and can be technically challenging, often requiring significant resources and care depending on factors such as fracture complexity, bone quality, functional demands, patient age, and comorbidities (Ziegler et al., 2020; Iglesias-Rodríguez et al., 2021; Relvas Silva et al., 2021; Handoll et al., 2022).

A recent systematic review evaluated the best methods to restore shoulder function and avoid adverse treatment outcomes. The review included 47 studies (3,179 participants with shoulder fractures, most of whom were women aged 60 years or older) conducted across 21 countries, with most studies following participants for at least one year. The authors concluded that there is still insufficient scientific evidence from randomized controlled trials to support definitive choices between conservative treatment and various surgical modalities for these fractures. Furthermore, even regarding conservative treatment, there was no conclusive evidence that one week of immobilization is better or worse than three or more weeks (Handoll et al., 2022).

In clinical practice, non-displaced or minimally displaced and stable fractures are typically eligible for conservative treatment, involving sling use for one or more weeks followed by rehabilitation. Conversely, displaced, unstable, open fractures or those associated with vascular injuries are generally treated surgically (Ratajczack et al., 2019; Petros et al., 2019; George et al., 2021; Handoll et al., 2022).

Surgical treatment involves various stabilization techniques, including pin fixation, rods, plates, or arthroplasty. A recent systematic review comparing outcomes of these techniques for displaced PHF found no significant differences among them or between them and conservative treatment. However, some trends emerged: Hemiarthroplasty showed better results in quality of life. Reverse arthroplasty demonstrated superior outcomes in abduction, flexion, Constant score, and lower rates of malunion, osteonecrosis, and the need for surgical reintervention. Open reduction and internal fixation (ORIF) yielded better results in the Quick-DASH disability score and the visual analog scale for pain (Davey et al., 2022).

ORIF with locking plates aims to preserve vascularization and stabilize fracture fragments sufficiently to allow early mobilization, resulting in a lower rate of reduction loss (Oldrini et al., 2022; Petros et al., 2019). In a series of 33 patients treated with angular stability locking plates for PHF, Monteiro et al. (2011) reported 91.2% excellent and good results using the UCLA (University of California at Los Angeles) functional score, deeming the method effective for stabilizing PHF without additional complications.

Ockert et al. (2014) described outcomes in 43 cases of displaced and unstable PHF classified according to Neer and AO/OTA criteria, treated with locking plates. After ten years of surgical treatment, most patients exhibited excellent or good results, with poor long-term outcomes occurring mainly in elderly female patients.

In a series of 35 cases treated with fixed-angle locking plates, George PK et al. (2021) concluded that effective stability achieved during surgery, maintained throughout follow-up, allowed early mobilization. This resulted in better range of motion (ROM) and quicker return to activities, with minimal reduction loss compared to other implants.

Another systematic review in the same year analyzed the best approaches to restore shoulder function and prevent adverse treatment outcomes, also evaluating 47 studies (3,179 participants, mostly older women) across 21 countries. Similar to earlier findings, the review concluded that there remains insufficient evidence from randomized controlled trials to guide decisions between conservative and surgical treatment modalities for PHF. Additionally, no definitive evidence was found to support the superiority of one or three weeks of immobilization during conservative treatment (Handoll et al., 2022).

**Rehabilitation**

After an upper limb fracture, patients are often referred to physiotherapy for rehabilitation, aiming to reduce pain, improve the range of motion, and progressively gain muscle strength in both surgical and conservative cases. Although these interventions have clear objectives, their approaches vary significantly and may include thermal modalities, electrophysical resources, manual therapy, kinesitherapy, application of dynamic orthoses, guidance, and education (Peluso et al., 2022).

Rehabilitation protocols following proximal humeral fractures (PHF) are not well defined in the literature. A recent systematic review (Schnackers et al., 2019) highlighted the scarcity of detailed physiotherapy protocols based on scientific evidence aimed at restoring patients' daily activities. Among the evaluated studies, the ProFHER clinical trial (PROximal Fracture of the Humerus Evaluation by Randomisation), published in 2015 (Handoll et al., 2015), was the only one to provide a complete description in this regard.

The ProFHER physiotherapy protocol progresses in phases. The timeline of these phases depends on various factors: age, fracture healing stage, pain tolerance, patient expectations, activity level, and general health. If the fracture is stable, phase 3 begins after sling removal at three weeks, and phase 4 starts three weeks later. Most patients regain functional status within six weeks, achieving independent shoulder function.

In 2019, Ratajczak et al. emphasized that the ideal treatment for PHF depends on the type of fracture, patient expectations, physical fitness level, and acceptance of the proposed treatment. They recommended adjusting exercises based on the fracture's stability and the patient's abilities and expectations. For surgical cases, they indicated that rehabilitation should begin immediately after immobilization removal, typically between the fourth and ninth weeks. Rehabilitation, according to the authors, should include passive exercises, active-passive exercises, and monitored active isometric, concentric, and eccentric exercises as the limb's function improves. They proposed a three-stage postoperative rehabilitation program using kinesitherapy:

**Stage 1: First 3 weeks**

- Immobilization with a sling on the operated limb without weight-bearing (except for arthroplasty).
- Initiation of pendulum exercises.
- Assisted passive movements.
- Avoid external rotation during the first 6 weeks.

**Stage 2: Third to ninth week (provided there are no abnormalities in healing or secondary fragment displacement):**

- Assisted active exercises, flexion, and abduction of the shoulder joint.
- Careful active exercises with limb abduction until pain occurs, with no weight-bearing in the first 6 weeks. Gradually add weight.

**Stage 3: After the ninth week**

- Isotonic, eccentric, and concentric exercises.
- For patients with radiographic fracture healing and joint contracture, passive stretching exercises with an experienced professional are recommended.

Monticone et al. (2021) compared the effectiveness of task-oriented exercises based on patients' specific work activities and occupational therapy versus conventional exercises. They found superior results in reducing disability, pain, and improving quality of life in patients after surgery for PHF. After early mobilization (at the end of the first postoperative week), basic exercises were introduced to improve glenohumeral mobility and muscle awareness in the upper limb. Patients learned techniques to progressively gain resistance, speed, strength, and complexity in the standard movements of the muscles involved in the injury.

Subsequently, exercises based on patients' specific work activities were introduced, including moving objects of different shapes and sizes in various directions, picking up objects from a table in different positions, assembling complex items by mixing components arranged on a table, and catching objects thrown at different heights and speeds. Additional exercises aimed to recover dexterity, balance, and other functional demands, such as transitioning from a sofa to a seated position, standing up from a chair, walking and turning at a preferred speed, and ascending and descending stairs and obstacles. Occupational therapists also provided guidance on sling care and ergonomic principles based on patients' pre-fracture activities.

As mentioned earlier, rehabilitation after fractures may also involve using electrophysical resources, including light sources.

**Photobiomodulation**

Photobiomodulation (PBM), formerly known as low-level laser therapy, is defined as the use of light sources to stimulate healing, relieve pain, and reduce inflammation (Hamblin, 2017). This therapy is safe, minimally invasive, and has no side effects (Hamblin, 2017; Sharma et al., 2023). The most commonly used sources for PBM are lasers and LEDs (light-emitting diodes) of low intensity or power in the red (600–700 nm) and near-infrared (770–1200 nm) wavelengths.

PBM action is based on light absorption by cellular chromophores, which can be categorized into three groups: mitochondrial cytochromes (proteins bound to the inner mitochondrial membrane, such as cytochrome C oxidase); opsins (3 and 4); and heat- or light-sensitive calcium ion channels, and nano-structured water clusters (Sharma et al., 2023). Light-sensitive ion channels are activated by light absorption by opsins, while mitochondrial cytochromes absorb red and near-infrared wavelengths. Light above 980 nm is likely absorbed by nano-structured water clusters, activating transient receptor potential (TRP) ion channels. Heat-sensitive TRPs may also be activated by minor temperature changes (Sharma et al., 2023).

Red or infrared light absorbed by mitochondrial cytochrome C oxidase may dissociate the inhibitory nitric oxide associated with it, increasing enzymatic activity, electron transport, mitochondrial membrane potential, and ATP production (Sharma et al., 2023). Another possible explanation for PBM effects is the activation of light-sensitive ion channels, allowing calcium influx into cells or mitochondria. Both pathways activate mitochondrial metabolism and numerous signaling pathways, such as reactive oxygen species (ROS), cyclic AMP, nitric oxide, and calcium ions. These pathways lead to transcription factor activation, enhancing gene expression related to protein synthesis, cell migration, proliferation, anti-inflammatory signaling, anti-apoptotic proteins, and antioxidant enzymes (de Freitas & Hamblin, 2016; Sharma et al., 2023).

Regarding PBM use in limb fracture treatment, a systematic review and meta-analysis published in 2020 highlighted the lack of randomized controlled trials (RCTs) on this topic, including only two low-evidence studies. Combined analysis of these RCTs revealed clinically and statistically significant differences favoring PBM in improving fractured limb function and significantly reducing pain. However, no differences were found regarding radiographic fracture healing (Neto et al., 2020). The review also emphasized the need for new RCTs following CONSORT (Consolidated Standards of Reporting Trials) recommendations, aiming to determine the best dosimetric parameters and evaluate potential adverse effects. Other reviews assessing PBM's role in bone repair using diverse methodologies concluded that PBM might accelerate bone regeneration. However, standardizing parameters and treatment protocols remains necessary (Deana et al., 2018; Escudero et al., 2019; Cheng et al., 2020).

Subsequently, Saebo et al. (2021) evaluated the effect of PBM applied during the plaster immobilization period (conservative, non-surgical treatment) on pain and stiffness in patients with distal radius fractures. In a double-blind RCT, 53 participants received nine PBM applications (904 nm, 25W pulse peak, 60 kHz, 60 mW average power, 6.6 J per session) and were assessed at 4, 8, 12, and 26 weeks post-trauma. The PBM-treated group showed significantly better results than the placebo group in range of motion and grip strength throughout the assessment period, with statistically significant differences observed after 4 weeks. The discrepancies between the injured and uninjured wrists of each patient were significantly smaller in the PBM group for grip and pinch strength at weeks 4 and 26. Additionally, significantly fewer patients in the PBM group reported nighttime pain during the third week of follow-up compared to the placebo group. However, no differences were found between the PBM and placebo groups in standardized wrist function and pain perception questionnaires, analgesic use, or local edema.

The same group of authors assessed the effect of PBM (904 nm, 25W pulse peak, 60 kHz, 60 mW average power, 7.2 J per session, 1.2 J per point), combined with exercises, applied after immobilization removal in 50 patients with distal radius fractures treated conservatively (non-surgically). The triple-blind RCT evaluated standardized wrist function and pain perception questionnaires, nighttime pain, and analgesic consumption at 4, 8, 12, and 26 weeks. Nighttime pain and analgesic use were also assessed after 7 weeks. The PBM group showed significantly better outcomes in standardized function and pain perception questionnaires at 8, 12, and 26 weeks. Nighttime pain and analgesic use were significantly lower in the PBM group between 7 and 26 weeks (Saebo et al., 2022).

Jana Neto et al. (2023) evaluated the effect of PBM with LEDs on the healing process of soft tissue injuries associated with surgically treated tibia fractures. The study included 27 adult individuals awaiting resolution of soft tissue injuries before definitive surgery. PBM (144 LED diodes at wavelengths of 420 nm, 660 nm, and 850 nm, 3 J per point, for 10 minutes) was applied daily until the soft tissues were ready for definitive surgery. The control group was treated with inactive devices identical in appearance. The PBM-treated group showed better daily healing rates, greater average daily reduction in pain scores, four times fewer infections at the pin sites of external fixators, and faster resolution (10 days earlier). No adverse effects were reported.

**Evaluation of Therapeutic Success in PHF Treatment**

Even after surgical or conservative treatment, PHF can still cause pain, limitations in daily activities, limb disability, and reduced quality of life. Clinical trials on PHF often report diverse outcomes, complicating treatment comparisons and consensus development (Nowak et al., 2019; Richard et al., 2020).

It has been suggested that the evaluation of therapeutic success in PHF treatment should primarily reflect the restoration of patients' daily activities, using tools known as Patient Reported Outcomes (PRO). For PHF follow-up, it is recommended to include at least one functional score (e.g., DASH - Disabilities of the Arm, Shoulder, and Hand; ASES - American Shoulder and Elbow Surgeon’s Score; or Oxford Shoulder Score) and PROs reflecting health status and quality of life, such as the EQ-5D - EuroQol-5 Dimension and the SF-6D - Short Form-6 Dimension (Nowak et al., 2019; Richard et al., 2020).

The DASH functional disability index, developed in 1996, is a self-administered instrument for measuring symptoms and physical function of the upper limbs. It can be used for clinical and research purposes. The DASH consists of questions related to daily activities, symptoms, and social/role functions over the past week. The 30 questions are scored on a scale of 1 to 5 points, with a total score ranging from 0 (no difficulty) to 100 (significant difficulties/disabilities) (Hudak et al., 1996). This tool has been adapted into over 50 languages and extensively validated in numerous scientific articles (Kennedy & Beaton, 2017; Richard et al., 2020). DASH has demonstrated strong reliability, moderately strong validity, and high psychometric properties in evaluating PHF patients (Slobogean et al., 2010; van de Water et al., 2014).

A shorter version, Quick DASH, was released in 2005, containing 11 items (Beaton et al., 2005). Like the original version, Quick DASH offers five response options and a total score of 100 points. Both questionnaires have optional modules, scored separately, designed for athletes/performers and work-related assessments (Kennedy & Beaton, 2017). Quick DASH can replace DASH for evaluating upper extremity disorders with similar accuracy, offering easier and faster administration (Beaton et al., 2005; Gummesson et al., 2006).

The Brazilian version of DASH was validated in 2006 (Orfale et al., 2006), while Quick DASH was more recently validated (da Silva et al., 2020), proving to be reliable, responsive, and a viable alternative to DASH for evaluating upper extremity dysfunctions related to orthopedic and traumatic injuries.

EQ-5D and SF-6D have shown strong reliability and moderately strong validity in PHF patients. EQ-5D also demonstrated good responsiveness and was recommended as a tool for assessing health-related quality of life in PHF patients in a systematic review (Olerud et al., 2011; Rabi et al., 2015; Richard et al., 2020).

EQ-5D-3L comprises five health domains (mobility, self-care, usual activities, pain/discomfort, anxiety/depression), each with three levels (no problems, some problems, extreme problems). Additionally, it includes a visual analog scale (VAS) where participants rate their health status from 0 ("Worst possible health state") to 100 ("Best possible health state"), generating 243 possible health states (EuroQol, 2010).

The SF-6 is derived from the Brazilian-adapted version of the SF-36 questionnaire (Medical Outcomes Study 36-Item Short-Form Health Survey), which has been evaluated in over 200 diseases and translated into 40 countries (Ware & Sherbourne, 1992; Garrat et al., 2002; Campolina et al., 2011).

**2. JUSTIFICATION**

There is substantial evidence highlighting the capacity of photobiomodulation (PBM) to control pain and assist in tissue repair. However, in the treatment of fractures, there is a need to develop randomized controlled trials (RCTs) with high methodological rigor to establish the best parameters and outcomes for PBM. In the case of PUF, besides the importance of finding the best dosimetric parameters for PBM application, there is a need to standardize the outcomes to be evaluated to obtain a gold standard treatment. This is because the majority of PUF cases present a slow recovery period accompanied by pain and functional limitations.

This study aims to assess the effects of PBM, using a home-based LED device with parameters supported by the literature, primarily concerning post-operative recovery of function and pain relief in surgically treated proximal humerus fractures. The study will be conducted in a controlled, randomized, and double-blind manner.

Hypotheses:

H0: Photobiomodulation is not effective in the post-operative phase of PUF.

H1: Photobiomodulation is effective in the post-operative phase of PUF.

**3. GENERAL OBJECTIVE**

The general objective of this study is to evaluate the effects of photobiomodulation (PBM) on the functional recovery of proximal humeral fractures treated with open reduction and internal fixation (ORIF) and stabilized with fixed-angle plates.

**3.1. Primary Objective**

The primary outcome of the study is to assess the functional recovery following proximal humeral fractures treated with ORIF, stabilized with fixed-angle plates, and managed with physiotherapy and photobiomodulation. This will be evaluated using the Brazilian version of the Quick DASH (Disabilities of the Arm, Shoulder, and Hand) questionnaire.

**3.2. Secondary Objectives**

The secondary outcomes will evaluate the effects of PBM applied after proximal humeral fractures treated with ORIF, stabilized with fixed-angle plates, and managed with physiotherapy on:

- Shoulder range of motion (ROM), assessed over time and compared with the unaffected limb.
- Muscle strength, measured over time and compared with the unaffected limb.
- Intensity of spontaneous pain and pain during limb function.
- Occurrence of nighttime pain.
- Pressure pain at the fracture site.
- Analgesic consumption.
- Fracture consolidation.
- Quality of life.
- Incidence of adverse events.
- Direct and indirect costs associated with the procedures performed during the study period, for subsequent cost-effectiveness analysis of the intervention.

**4. MATERIAL AND METHODS**

This is a parallel, double-blind, randomized controlled clinical trial. The study design will adhere to international recommendations for randomized clinical trials outlined in the SPIRIT (Standard Protocol Items: Recommendations for Interventional Trials - Chan et al., 2013) protocol, as shown in Figure 1, and the regulatory norms for research involving human subjects established by the National Research Ethics Committee (CONEP).

This research project will be submitted to the Research Ethics Committee of Universidade Nove de Julho (UNINOVE), and participants will sign the informed consent form after receiving all necessary explanations (APPENDIX 1). The protocol will be registered on the Clinical Trials platform (<https://clinicaltrials.gov/>). The study report will be conducted in accordance with the CONSORT (Consolidated Standards of Reporting Trials) guideline. The study flowchart is presented in Figure bellow.

###
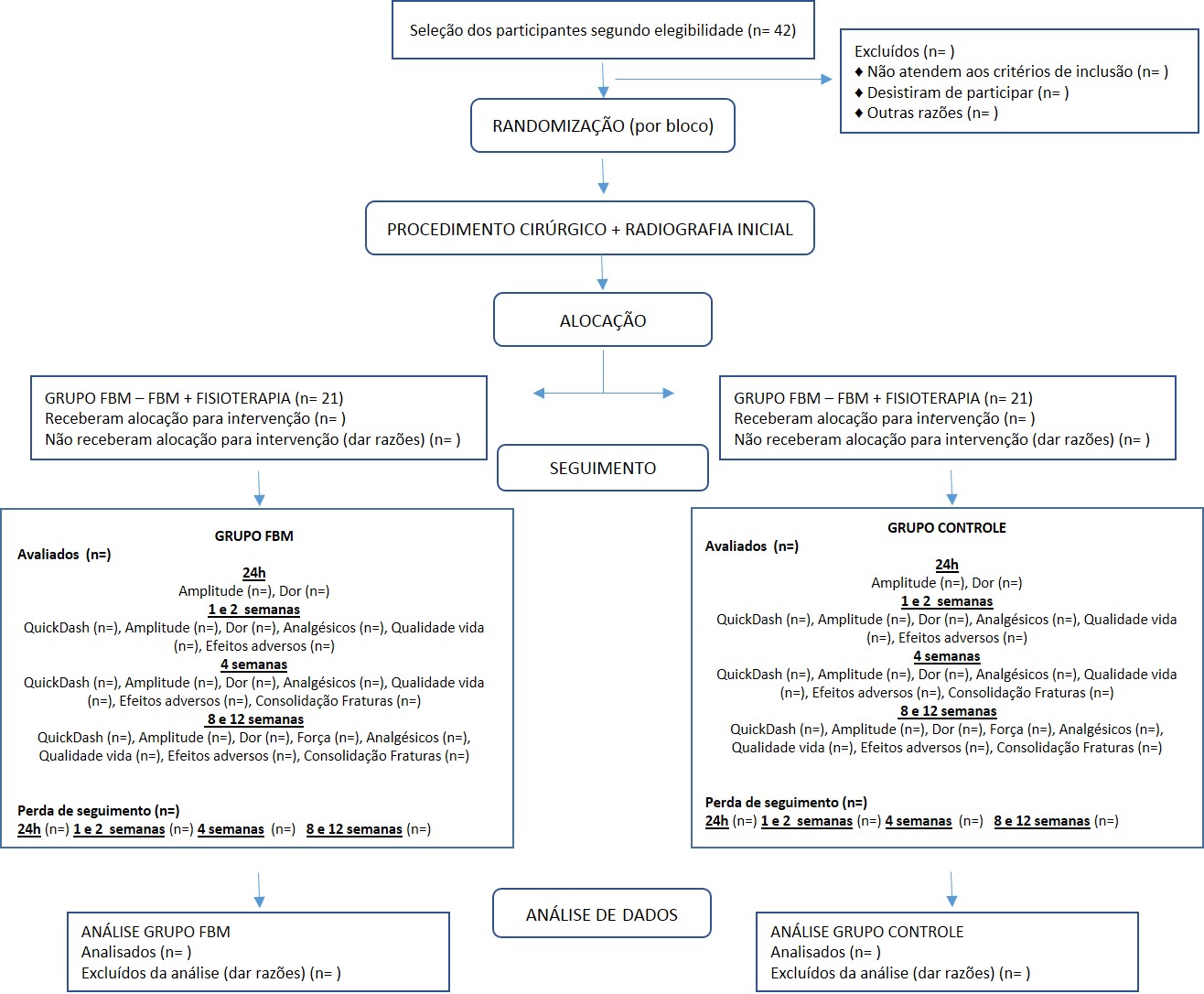


**4.1 Study Location and Sample Selection**

The surgical procedures, outpatient follow-ups, and physiotherapy will be conducted at the Orthopedics and Traumatology Clinic of Dr. Alípio Correa Netto Hospital (HMACN), part of the Municipal Health Department of São Paulo, Brazil, between April 2024 and December 2025.

**4.2 Research Team**

The research team will consist of:

- **1 Researcher**: Responsible for preparing the randomization process and envelopes to ensure allocation confidentiality. This researcher will not participate in any evaluations.
- **1 Researcher (medical resident)**: Responsible for retrieving the allocation envelope upon participant enrollment, delivering the appropriate PBM equipment (active or placebo), and providing instructions for home use.
- **4 Surgeons with experience in proximal humerus fractures (PHF)**: Responsible for all surgical procedures and participant eligibility evaluations.
- **4 Blinded Orthopedic Medical Examiners**: Responsible for all evaluations, from postoperative assessments to outpatient follow-ups, while remaining blinded to the experimental group of each participant.
- **1 Principal Researcher**: An orthopedic physician aware of group allocations during the data collection phase and responsible for coordinating data collection. This researcher will not participate in any evaluations and will oversee data interpretation.
- **1 Physiotherapist (blinded to the experimental group)**: Responsible for accompanying participants during all sessions.
- **3 Medical Residents (blinded to the experimental group)**: Responsible for daily contact with participants to monitor PBM device usage, provide guidance, and address any questions.

**4.3 Examiner Calibration and Training**

The training and calibration process for the four researchers conducting postoperative evaluations will include three joint practice sessions with one volunteer who will not be part of the experimental groups. The data collected during these sessions will be discussed among the researchers to achieve excellent agreement levels. Subsequently, each examiner will independently perform the proposed measurements on ten adult volunteers who will also not be part of the study sample. The data collected will be analyzed using the Intraclass Correlation Coefficient (ICC) test (Fleiss, 1986). Once the agreement among examiners is rated as excellent, the measurements will be applied to the study participants.

**4.4 Participant Characterization**

Participants of both genders admitted to HMACN with isolated, closed proximal humerus fractures requiring surgical treatment and treated with ORIF using a locked angular stability plate for the proximal humerus (Neer 1070, Carrera et al., 2012; Petros, 2019) will be included according to the following eligibility criteria.

**Inclusion Criteria:**

- Participants aged 18 to 65 years of both genders, with PHF classified as:
  - Neer group III
  - Neer group IV
  - Neer group V
  - AO/OTA subgroups A2 and A3
  - AO/OTA group B
  - AO/OTA group C (only in patients younger than 55 years).

**Exclusion Criteria:**

- Individuals with prior injuries or sequelae in the shoulder or scapular girdle, or motor deficits due to central or peripheral neurological injuries.
- Patients with pathological fractures.
- Participants with postoperative infection or implant loosening.
- Those with ipsilateral fractures in other areas of the limb.
- Patients with neurovascular injuries causing sensory deficits at the injury site.
- Individuals with local or systemic conditions contraindicating surgical intervention or complicating the postoperative period.
- Participants with a history of photosensitivity.
- Individuals with neurological or psychiatric disorders.
- Those with proliferative or infectious skin lesions in the shoulder area where LED light will be applied.
- Participants who used anti-inflammatory medications within 5 days prior to the trauma.
- Pregnant individuals.
- Participants who experience surgical complications, such as neurological or vascular injuries or fracture line extension during surgery, as they will not meet the desired progression criteria.

Such participants will be excluded from statistical analyses but will have their data collected and documented.

**4.5 Sample Size Calculation**

The sample size required for each experimental group was calculated based on the variability of results from the study by Chang et al. (2014), which evaluated the effects of PBM with a laser on functional recovery of wrist and hand fractures using the Quick DASH questionnaire. Using the power/sample size calculator developed by the University of British Columbia (available at <https://www.stat.ubc.ca/~rollin/stats/ssize/n2.html>), the required sample size was determined to be 42 participants, with 21 per group. Calculations were based on a significance level of 0.05, 90% power, and a 15% loss rate, considering the highest obtained value.

**4.6 Group Composition**

Participants will be divided into two groups:

- **PBM Group**: Participants will receive active PBM administered at home, combined with conventional therapeutic treatment. The equipment, dosimetry parameters, and PBM application frequency are described in item 4.10.
- **Control Group**: Participants will receive placebo PBM administered at home, combined with conventional therapeutic treatment. The equipment description for placebo application is detailed in item 4.10.

**4.7 Randomization and Allocation Concealment**

The sequence generation and envelope preparation will be conducted by a researcher not involved in the study.

Participants will be randomly assigned to the two experimental groups using a random sequence generator (<https://www.randomizer.org/tutorial/>), with the option for block randomization of six participants, forming seven blocks. Opaque envelopes will be numbered sequentially, each containing a sheet with the corresponding experimental group based on the generated order. The envelopes will be sealed and stored in numerical order in a secure plastic container.

Participants meeting all previously described eligibility criteria will be evaluated by four surgeons and included in the study. All participants will undergo the same surgical protocol. The allocation envelope will be opened 24 hours after the surgical procedure when the PBM device is delivered. The device delivery will be performed by the medical resident researcher, who will be aware of the allocations.

**4.8 Initial Procedures and Participant Recruitment**

Participants will be recruited from patients presenting to the emergency room at Dr. Alípio Correa Netto Hospital (HMACN) with shoulder trauma and suspected fractures. All patients will receive treatment according to the hospital's standard protocol.

The cases will be evaluated by the on-call orthopedic team, which will notify the research team. In the emergency room, the examining researchers will request standard imaging tests, including anteroposterior, scapular profile, and axillary shoulder radiographs, and CT scans when indicated. Preoperative tests, including complete blood count, coagulation profile, blood typing, serum levels of sodium, potassium, urea, creatinine, glucose, electrocardiogram, and chest X-rays (posteroanterior and lateral views), will also be requested. Echocardiograms and consultations with other specialties will be requested when necessary.

Potential study inclusion will be determined only after clinical and radiographic evaluations confirm eligibility. Participants will receive verbal and written explanations regarding the study's objectives and methods. Those who agree to participate will sign the informed consent form (Appendix 1).

**4.9 Medication Prescription**

During hospitalization, analgesic prescriptions will include dipyrone 1g IV every 6 hours and tramadol hydrochloride 100 mg IV every 8 hours, maintained for the first 24 hours. If rescue analgesics are needed, the first option will be to administer tramadol hydrochloride 100 mg IV every 6 hours. Morphine sulfate will be the second rescue drug, administered at a dose of 2 mg IV up to every 8 hours. Patients will be reassessed for adjustments to the standard analgesic regimen, transitioning to "as needed" prescriptions for opioids when possible. All analgesic drug use will be documented. Corticosteroids or anti-inflammatory drugs will not be prescribed.

Upon discharge, participants will be prescribed dipyrone 1g orally every 6 hours for 5 days and tramadol hydrochloride 50 mg orally every 8 hours (if needed) for a maximum of 5 days. Participants will be instructed to use tramadol hydrochloride only if pain persists with dipyrone or in cases of moderate pain (4–7 on the pain scale). Participants reporting anti-inflammatory or corticosteroid use will be excluded from statistical analysis, and the case will be documented and discussed.

**4.10 Surgical Procedure**

All participants will undergo general anesthesia combined with brachial plexus block and a standard surgical procedure as practiced at the Orthopedics Department of HMACN.

Fractures will be approached using a deltopectoral incision with the patient in a beach chair position. Careful dissection will be performed to avoid excessive exposure of fragments and preserve vascularization. Fragments will be anatomically reduced using indirect maneuvers and temporarily stabilized with Kirschner wires (Biomecânica, Jaú, SP, Brazil). Definitive stabilization will be achieved with an anatomical locked proximal humerus plate (GM Reis, Campinas, SP, Brazil) of 3.5 mm, the gold standard for PHF stabilization (Ratajczak et al., 2019; Oldrini et al., 2022). The plate will be positioned 1 cm below the superior edge of the greater tuberosity and 1 cm lateral to the long head of the biceps tendon.

The reduction, plate positioning, and screw placement will be evaluated intraoperatively using fluoroscopy.

Parte inferior do formulário


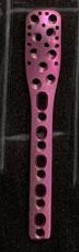


Figure –Blocked plate. Source: own author.

**4.11 Physiotherapy**

All participants will follow a standardized physiotherapy protocol as suggested by Ratajczak et al. (2019) and the AO Trauma group (<https://www.aofoundation.org/trauma>), detailed below:

**Stage 1: First 3 weeks, starting 24 hours after surgery**

- Immobilization with a sling for the operated limb without weight-bearing.
- Initiation of pendulum exercises.
- Assisted passive movements.
- Avoid external rotation during the first 6 weeks.

**Stage 2: Third to ninth week, provided there are no abnormalities in consolidation or secondary displacement of fragments**

- Assisted active exercises, including flexion and abduction of the shoulder joint.
- Careful active exercises with limb abduction until pain occurs, with no weight-bearing in the first 6 weeks. Gradual weight addition will follow.

**Stage 3: After the ninth week**

- Isotonic, eccentric, and concentric exercises.
- For participants with radiographic fracture consolidation (evaluated at the 12th week) and joint contracture, passive stretching exercises will be recommended under the guidance of an experienced professional.

Participants will attend 30-minute physiotherapy sessions twice weekly for 12 weeks.

**4.12 Photobiomodulation (PBM) Application**

PBM will be administered using LED devices in the form of a shoulder brace. Applications will commence 24 hours after surgery and will continue daily throughout the experimental period. Participants will perform the applications at home, receiving guidance on how to wear and operate the equipment upon hospital discharge, along with written instructions (Appendix 9). Each application will last 10 minutes.

The shoulder brace, manufactured by Cosmedical (Mauá, SP, Brazil), contains 159 red LEDs and 159 infrared LEDs arranged alternately. The area corresponding to the fixation plate, with a 2 cm safety margin added along its entire length, will not be irradiated (LEDs will not be placed there) to avoid potential heating of the plate from energy absorption.

Participants allocated to the control group will receive a device identical to the active one. However, only the activation plug light and sound will turn on when the button is pressed, leaving the internal LEDs deactivated


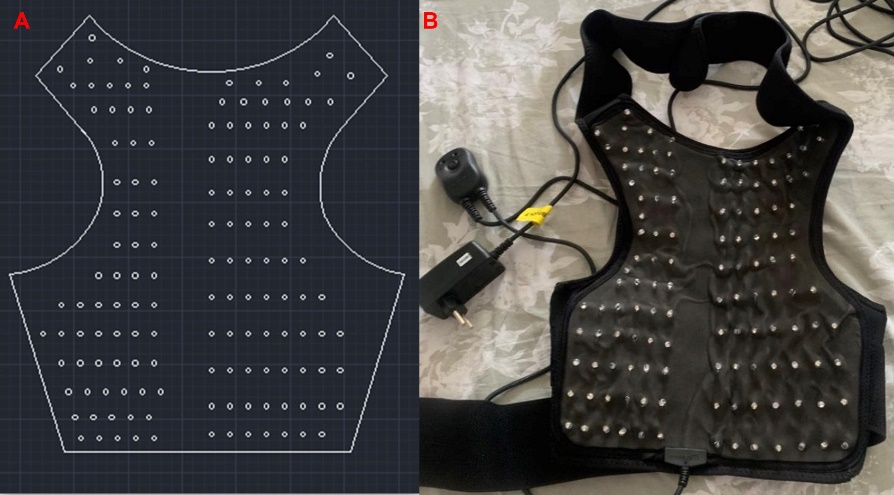


Figure . Design (A) and model (B) of the shoulder brace for PBM application.

Literature reports on the use of PBM after bone fractures indicate the use of red and infrared light sources with varying dosimetric parameters (Table 1), with radiant exposures ranging from 3 to 16 J/cm². The study by Chang et al. (2014), which served as the basis for the sample size calculation in this project, demonstrated positive effects of PBM on functional recovery of wrist and hand fractures using the Quick DASH questionnaire, with a radiant exposure of 9.7 J/cm². Accordingly, this project adopted radiant exposures of 10 J/cm² and 12 J/cm²

| **Wavelength (nm)** | **Power (mW)** | **Irradiance (mW/cm²)** | **Time (sec)** | **Radiant Exposure (J/cm²)** | **Radiant Energy (J) per Point** | **Number of Applications** | **Application Points** | **Results** | **Authors** |
| --- | --- | --- | --- | --- | --- | --- | --- | --- | --- |
| 830 | - | - | - | 8 and 16 | - | Once per day | Sensitive points | Earlier symptom resolution and ambulation | Chauhan & Sarin (2005) |
| 830 | 60 (average power) | 16.2 ± 1 | 600 | 9.7 | 2.5 | 10 | 1 | Pain relief and improved consolidation in conservative wrist fracture treatment | Chang et al. (2014) |
| 808 & 650 | 300 & 100 | 30 ± 20 | 20 ± 30 | 6 ± 3 | 6 ± 3 | 1 | 10 to 12 | Less opioid consumption in tibia fracture treatment | Nesioonpour et al. (2014) |
| 904 | 60 (average power) | 120 | 120 | 3 and 1.2 | 9 | 6 | Improved nighttime pain after 3 weeks; better ROM and grip strength | - |  |
| 904 | 60 (average power) | 120 | - | 1.2 | 9 | 6 | Significant improvement in functional score (PRWHE) after 8, 12, and 26 weeks |  | Jana Neto (2023) |

In the present study, the dosimetric parameters (Table 2) will combine higher and lower energy wavelengths (red and infrared, respectively) with higher and lower penetration depths (infrared and red, respectively), ensuring energy per point and radiant exposures within the limits reported in the literature. To calculate the parameters, the radiant power of each LED was measured using a power meter with a pyroelectric sensor (Coherent, Field Max2 model, Wilsonville, OR, United States).


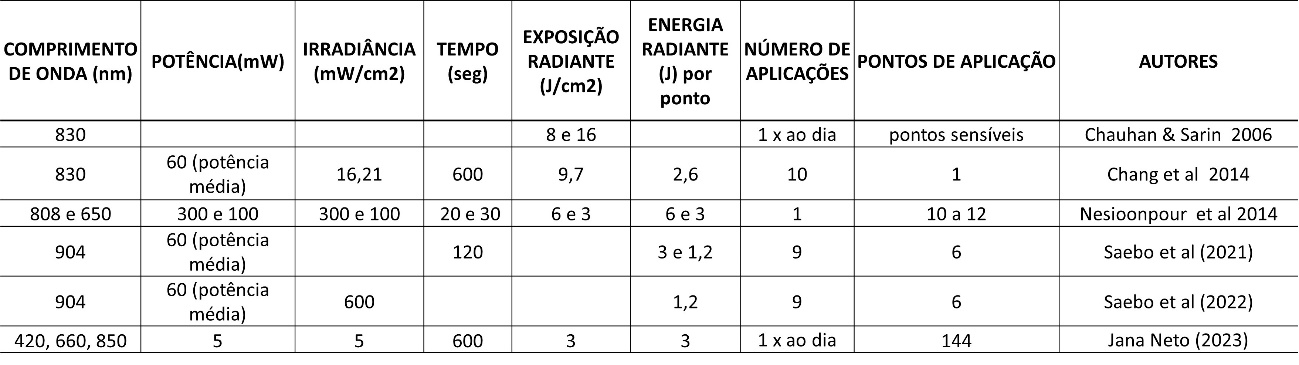


In this study, the dosimetric parameters (Table 2) will allow the combination of photons with higher and lower energy (red and infrared, respectively), greater and lesser penetration (infrared and red, respectively), with energy per point and radiant exposures within the limits reported in the literature. The radiant power of each LED was measured using a power meter with a pyroelectric sensor (Coherent, Field Max2 model, Wilsonville, OR, United States).

**Table 2: Dosimetric Parameters of the PBM Device**

| **Parameter** | **PBM Red** | **PBM Infrared** |
| --- | --- | --- |
| Central Wavelength [nm] | 660 | 850 |
| Spectral Bandwidth (FWHM) [nm] | 19 | 30 |
| Operation Mode | Continuous | Continuous |
| Radiant Power per LED [mW] | 28.5 | 23 |
| Number of LEDs | 159 | 159 |
| Total Radiant Power [mW] | 4531.5 | 3657 |
| Energy per LED [J] | 17 | 14 |
| Polarization | Random | Random |
| Aperture Diameter per LED [mm] | 3 | 3 |
| Irradiance at Aperture [mW/cm²] | 20.4 | 16.5 |
| Beam Profile | Multimode | Multimode |
| Exposure Time (s) | 600 | 600 |
| Radiant Exposure per LED [J/cm²] | 12 | 10 |
| Radiant Energy per Session [J] | 2719 | 2194 |
| Application Mode | Direct contact with the skin |  |
| Session Frequency | Once daily for 12 weeks |  |

**4.13 Daily Telephone Follow-Up**

Three medical residents will conduct daily telephone follow-ups with participants to monitor the use of the PBM device, provide guidance, address questions, and record data on spontaneous and nighttime pain, as well as analgesic use. The first contact will be made via video call to reiterate instructions on equipment positioning and use.

During the first five days, participants will specifically be asked about their use of dipyrone 1g orally every 6 hours and tramadol hydrochloride 50 mg orally every 8 hours (prescribed for use as needed).

In addition to monitoring usage and outcomes, the telephone follow-up aims to improve participant adherence. During these calls, participants can ask questions or request additional guidance. The research team will only contact participants after they have been discharged from the hospital, after receiving all instructions and signing the informed consent form (TCLE) in person.

The form template for this evaluation is included in **Appendix 2**, and the commitment form for equipment delivery is provided in **Appendix 10**.

A single cell phone, with a number exclusively designated for the project, will be kept at the hospital. Text or voice messages will not include sensitive data and will be deleted upon project completion.

**4.14 Intervention Failure**

Therapy failure will be characterized if any of the following occurs:

1. Inability to apply PBM daily.
2. The participant develops bleeding, sepsis, urticaria, or any discomfort justifying the discontinuation of PBM or placebo PBM.
3. Discontinuation of treatment for any other reason. In these cases, data collected up to that point will be included in the statistical analysis using a mixed-effects regression model.
4. Failure to attend in-person evaluations or respond to scheduled virtual contacts. Data collected up to this point will also be included in the statistical analysis using a mixed-effects regression model.

**4.15 Outcome Evaluation**

**4.15.1 QuickDASH Questionnaire (Disabilities of the Arm, Shoulder, and Hand)**

This study will use the dysfunction/symptom component of the QuickDASH score, validated for Brazil, available online, and included in **Appendix 1** and **Appendices 3 to 8**. (<https://dash.iwh.on.ca/sites/dash/public/translations/Scoring_QuickDASH_Portuguese_Brazil.pdf>).

The Dysfunction and Symptom Score will be calculated according to the instructions provided online, as follows:

- At least 10 out of 11 items must be answered.
- Each answered question will have a maximum value of 5.
- Scores will be transformed to a 100-point scale by subtracting 1 and multiplying by 25. This transformation facilitates comparison with other 0–100 scales. A higher score indicates greater dysfunction.

**QuickDASH Score Calculation**:

QuickDASH Score=[(Sum of responsesn)−1]×25\text{QuickDASH Score} = \left[\left(\frac{\text{Sum of responses}}{n}\right) - 1\right] \times 25QuickDASH Score=[(nSum of responses​)−1]×25

Where nnn is the number of answered questions.

The QuickDASH will be administered at 1, 2, 4, 8, and 12 weeks post-surgery.

**4.15.2 Range of Motion**

Shoulder range of motion (ROM) will be assessed on both sides with the participant in an upright position. The maximum tolerated positions in extension, flexion, abduction, adduction, lateral rotation, and medial rotation will be recorded by the evaluator (Figure 9). Rotations will be assessed at 0° abduction and flexion (Gracitelli, 2015; Tenor Junior et al., 2016). All measurements will be performed using a digital goniometer (Kaptron 360, Shenzhen, Dongguan, China).


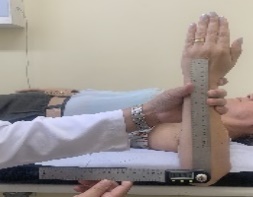

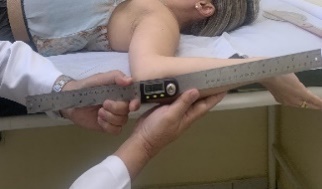

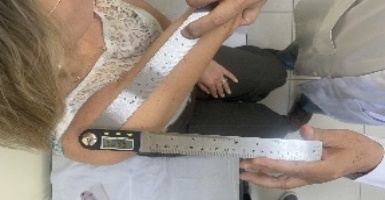

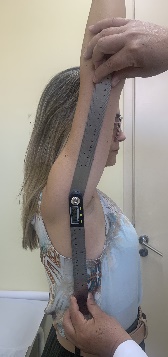

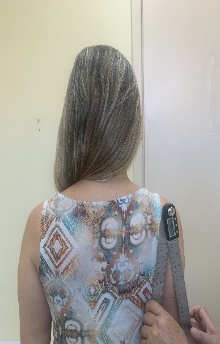

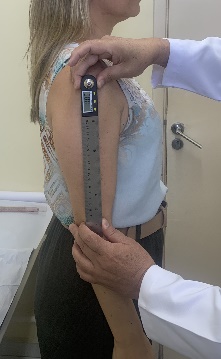

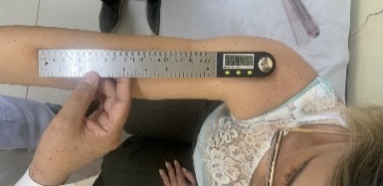

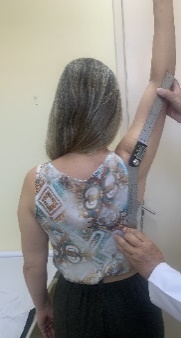

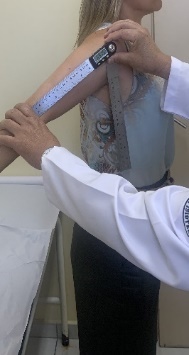

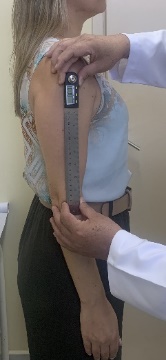


E

A

B

C

D


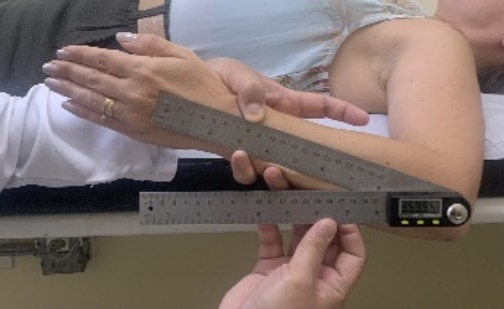

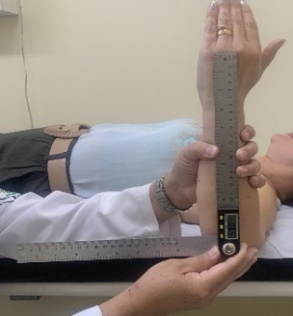


F

**Figure.** Measurement of range of motion (ROM) using a digital goniometer during flexion (A), extension (B), abduction (C), adduction (D), lateral rotation (E), and medial rotation (F), with the goniometer in position.

The ROM assessment will be performed 1 day after surgery and subsequently at 1 and 2 weeks through passive movements. During evaluations at 4, 8, and 12 weeks, active movements will be assessed. Results will be recorded on the corresponding clinical forms for each experimental period (**Appendices 3 to 8**).

**4.15.3 Muscle Strength**

Muscle strength will be evaluated at 8 and 12 weeks by progressive dumbbell lifting (RLM, Maringá, Paraná, Brazil) with elbow flexion in both arms. Progressive lifting will begin with 500 grams in the unaffected arm and increase to the maximum weight the participant can sustain, not exceeding 5 kg (Barbosa et al., 2008). Each dumbbell weight will be lifted only once per arm. Results will be recorded on the corresponding clinical forms for each experimental period (**Appendices 7 and 8**).

**4.15.4 Pain Assessment**

The intensity of spontaneous pain and pain during the function of the injured limb will be evaluated using the visual analog scale (VAS) for pain (Jensen et al., 1986). The chosen scale, represented in Figure consists of a 10 cm numbered line featuring facial expression illustrations, a color gradient, and indications of mild, moderate, and severe intensities to facilitate understanding (Thong et al., 2018).


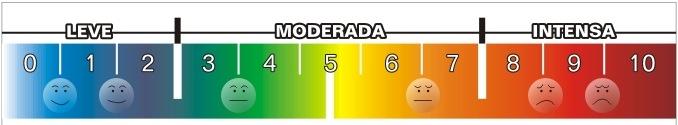


**Figure 11.** Visual analog pain scale.

Pressure pain at the fracture site (**Figure**) will be assessed using a digital algometer (MED DOR, Governador Valadares, MG, Brazil).


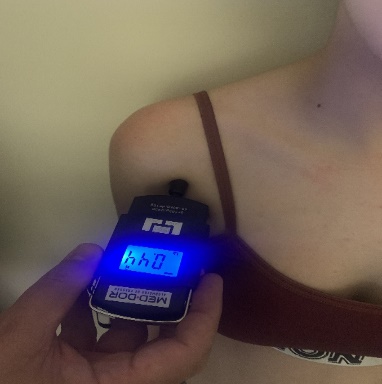


**Figure 12.** Digital algometer in position for measuring pressure pain.

Results regarding spontaneous pain, pain during function (flexion and abduction), and pressure pain measured using the algometer will be collected 1 day after surgery and subsequently at 1, 2, 4, 8, and 12 weeks. These results will be recorded in the respective clinical forms (Appendices 3 to 8) for each experimental period.

Nighttime pain occurrence will be assessed during daily PBM usage monitoring and recorded in the participant's daily control form (Appendix 2).

**4.15.5 Analgesic Consumption**

The type and dosage of analgesics consumed will be assessed during daily PBM usage monitoring and recorded in the participant's daily control form (**Appendix 2**).

**4.15.6 Quality of Life**

Quality of life will be evaluated using the SF-6 instrument from 2002 (Appendix 3) in its version adapted for use in Brazil (Campolina et al., 2011). Assessments will take place at 1, 2, 4, 8, and 12 weeks, with results recorded in the respective clinical forms (Appendices 3 to 8) for each experimental period.

In the most recent Brazilian version of SF-6 (2002), as in the original, six items are included:

- Functional capacity (items 1, 2, and 10)
- Global limitation (item 3 for physical aspects and item 2 for emotional aspects)
- Social aspects (item 2)
- Pain (all items)
- Mental health (item 1)
- Vitality (item 2) (Campolina et al., 2011).

**4.15.7 Adverse Events**

The occurrence of adverse events will be assessed during daily PBM usage monitoring and recorded in the participant's daily control **form (Appendix 2).**

**4.15.8 Fracture Consolidation**

Shoulder X-rays (anteroposterior, scapular profile, and axillary views) will be taken at 4, 8, and 12 weeks to evaluate bone consolidation. Consolidation will be defined by the presence of a callus in three out of four bone cortices uniting the main fracture fragments, compared with the immediate postoperative X-ray (Gracitelli, 2015).

**4.15.9 Cost Analysis**

The costs will be calculated from the perspective of the Unified Health System (SUS) as the service purchaser. Direct costs of the procedures performed during the study period will be considered, including material costs, professional fees, hospital expenses, and productivity loss due to work absence.

Cost references in Brazilian currency (Real) will be sourced from the ComprasNet, SIGTAP, and Price Database of the Ministry of Health. The final cost-effectiveness and incremental cost analysis will follow the guidelines of the Methodological Guidelines: Economic Evaluation Studies of Health Technologies and the National Policy for Health Technology Management (BRASIL, 2010).

**Table 3: Treatment Costs**

| **Costs** | **Estimated Value (R$)** |
| --- | --- |
| Daily hospital stay in public network ward |  |
| Orthopedic care with provisional immobilization |  |
| Emergency room fee |  |
| Cast room fees |  |
| Antiseptic solution |  |
| Suture threads |  |
| Surgical compress |  |
| Surgical arch/intensifier usage |  |
| Pneumatic drill for surgery usage |  |
| Aspirator usage |  |
| Oximeter usage |  |
| Multifunction monitor (per hour) |  |
| Anesthesia machine usage |  |
| Dipyrone Monohydrate 2 ml ampoule |  |
| Tramadol Hydrochloride 100 mg ampoule |  |
| Enoxaparin 40 mg ampoule |  |
| Omeprazole 40 mg ampoule |  |
| Ondansetron 8 mg ampoule |  |
| Cefazolin Sodium 1 g ampoule |  |
| Morphine Sulfate 2 ml ampoule |  |
| Imaging exams (CT and X-rays) |  |
| Sterile gauze 13 threads |  |
| Cremer elastic bandage 10 cm x 1.8 m |  |
| Hospital dressing change |  |
| Daily cost of absent worker (indirect cost) |  |
| Disability aid (estimated, indirect cost) |  |
| Physiotherapy session |  |
| PBM device |  |
| **TOTAL** |  |

**2. Data Analysis**

Initial descriptive analyses will be performed considering all variables measured in the study, both quantitative (mean and standard deviation) and qualitative (frequencies and percentages). Subsequently, normality tests will be conducted to determine the appropriate statistical tests for each data set. Appropriate statistical tests will then be applied for each specific analysis.

Subgroup analyses will also be performed, considering variables such as the presence of osteoporosis, osteopenia, high- and low-intensity traumas, smoking, and other comorbidities.

A significance level of 5% probability or the corresponding p-value will be adopted for all tests. All analyses will be conducted using the **SAS for Windows** statistical software, version 9.1.

# **BIBLIOGRAFIA**

Abhishek gujar, Mitushi Deshmukh. An overview on low laser therapy in distal radius fracture. Journal of Pharmaceutical Negative Results [Internet]. 2022 Oct. 17 [cited 2022 Nov. 10]:2930-2

Alispahic N, Brorson S, Bahrs C, Joeris A, Steinitz A, Audigé L. Complications after surgical management of proximal humeral fractures: a systematic review of event terms and definitions. BMC Musculoskelet Disord. 2020 May 26;21(1):327. doi: 10.1186/s12891-020-03353-8. PMID: 32456631; PMCID: PMC7251821.

Audigé L, Brorson S, Durchholz H, Lambert S, Moro F, Joeris A. Core set of unfavorable events of proximal humerus fracture treatment defined by an international Delphi consensus process. BMC Musculoskelet Disord. 2021 Nov 30;22(1):1002. doi: 10.1186/s12891-021-04887-1. PMID: 34847888; PMCID: PMC8630858.

Barbosa RI, Marcolino AM, Fonseca MCR, Mazzer N, Zatiti SC. Retrospective functional assessment of patients with humerus proximal fractures fixed internally with a fixed-angle plate of the proximal humerus. Acta Ortop Bras 16(2): 89-92, 2008

Barreto RPG, Barbosa MLL, Balbinotti MAA, Mothes FC, Rosa LHT, Silva MF. The Brazilian version of the Constant-Murley Score (CMS-BR): convergent and construct validity, internal consistency, and unidimensionality. Revista Brasileira de Ortopedia [online]. 2016, v. 51, n. 05 [Accessed 15 December 2022], pp. 515-520. Available from: <https://doi.org/10.1016/j.rboe.2016.08.017>.<https://doi.org/10.1016/j.rboe.2016.08.017>.

Beaton DE, Wright JG, Katz JN; Upper Extremity Collaborative Group. Development of the QuickDASH: comparison of three item-reduction approaches. J Bone Joint Surg Am. 2005 May;87(5):1038-46. doi: 10.2106/JBJS.D.02060. PMID: 15866967.

Bougher H, Nagendiram A, Banks J, Hall LM, Heal C. Imaging to improve agreement for proximal humeral fracture classification in adult patient: A systematic review of quantitative studies. J Clin Orthop Trauma. 2020 Feb;11(Suppl 1):S16-S24. doi: 10.1016/j.jcot.2019.06.019. Epub 2019 Jun 26. PMID: 31992911; PMCID: PMC6977161.

Brasil. Ministério da Saúde. Secretaria de Ciência, Tecnologia e Insumos Estratégicos. Departamento de Ciência e Tecnologia. Política Nacional de Gestão de Tecnologias em Saúde / Ministério da Saúde, Secretaria de Ciência, Tecnologia e Insumos Estratégicos, Departamento de Ciência e Tecnologia. – Brasília: Ministério da Saúde, 2010. 48 p. – (Série B. Textos Básicos em Saúde).

Brorson S, Elliott J, Thillemann T, Aluko P, Handoll H. Interventions for proximal humeral fractures: key messages from a Cochrane review. Acta Orthop. 2022 Jul 4;93:610-612. doi: 10.2340/17453674.2022.3495. PMID: 35819454; PMCID: PMC9275419.

Campolina AG, Bortoluzzo AB, Ferraz MB, Ciconelli RM. O questionário SF-6D Brasil: modelos de construção e aplicações em economia da saúde. Rev Assoc Med Bras [Internet]. 2010;56(Rev. Assoc. Med. Bras., 2010 56(4)). Available from: https://doi.org/10.1590/S0104-42302010000400012

Campolina AG, Bortoluzzo AB, Ferraz MB, Ciconelli RM. Validação da versão brasileira do questionário genérico de qualidade de vida short-form 6 dimensions (SF-6D Brasil). Ciênc saúde coletiva [Internet]. 2011Jul;16(Ciênc. saúde coletiva, 2011 16(7)). Available from: https://doi.org/10.1590/S1413-81232011000800010

Carrerra, Eduardo da Frota et al. Reproducibility of three classifications of proximal humeral fractures. Einstein (São Paulo) [online]. 2012, v. 10, n. 4 [Accessed 3 November 2022], pp. 473-479. Available from: <https://doi.org/10.1590/S1679-45082012000400014>. Epub 22 Jan 2013. ISSN 2317-6385. <https://doi.org/10.1590/S1679-45082012000400014>.

Chan AW, Tetzlaff JM, Altman DG, Laupacis A, Gøtzsche PC, Krleža-Jerić K, Hróbjartsson A, Mann H, Dickersin K, Berlin JA, Doré CJ, Parulekar WR, Summerskill WS, Groves T, Schulz KF, Sox HC, Rockhold FW, Rennie D, Moher D. SPIRIT 2013 statement: defining standard protocol items for clinical trials. Ann Intern Med. 2013 Feb 5;158(3):200-7. doi: 10.7326/0003-4819-158-3-201302050-00583.

Chang WD, Wu JH, Wang HJ, Jiang JA (2014) Therapeutic outcomes of low-level laser therapy for closed bone fracture in the human wrist and hand. Photomed Laser Surg 32(4):212–218.

Chauhan A, Sarin P. Low Level Laser Therapy in Treatment of Stress Fractures Tibia: A Prospective Randomized Trial. Med J Armed Forces India. 2006 Jan;62(1):27-9. doi: 10.1016/S0377-1237(06)80148-6. Epub 2011 Jul 21. PMID: 27407838; PMCID: PMC4923284.

Cheng W, Yao M, Sun K, Li W. Progress in Photobiomodulation for Bone Fractures: A Narrative Review. Photobiomodul Photomed Laser Surg. 2020 May;38(5):260-271. doi: 10.1089/photob.2019.4732. PMID: 32427551.

Constant CR, Murley AH. A Clinical method of functional assessment of the shoulder. Clin Orthop Relat Res. 1987;(214):160-4.

Court-Brown CM, Caesar B. Epidemiology of adult fractures: A review. Injury. 2006 Aug;37(8):691-7. doi: 10.1016/j.injury.2006.04.130. Epub 2006 Jun 30. PMID: 16814787.

da Silva NC, Chaves TC, Dos Santos JB, Sugano RMM, Barbosa RI, Marcolino AM, Mazzer N, Fonseca MCR. Reliability, validity and responsiveness of Brazilian version of QuickDASH. Musculoskelet Sci Pract. 2020 Aug;48:102163. doi: 10.1016/j.msksp.2020.102163. Epub 2020 Apr 5. PMID: 32560867.

Davey MS, Hurley ET, Anil U, Condren S, Kearney J, O'Tuile C, Gaafar M, Mullett H, Pauzenberger L. Management options for proximal humerus fractures - A systematic review & network meta-analysis of randomized control trials. Injury. 2022 Feb;53(2):244-249. doi: 10.1016/j.injury.2021.12.022. Epub 2021 Dec 15. PMID: 34974908.

Deana AM, de Souza AM, Teixeira VP, Mesquita-Ferrari RA, Bussadori SK, Fernandes KPS. The impact of photobiomodulation on osteoblast-like cell: a review. Lasers Med Sci. 2018 Jul;33(5):1147-1158. doi: 10.1007/s10103-018-2486-9. Epub 2018 Mar 23. PMID: 29572767.

de Freitas LF, Hamblin MR. Proposed Mechanisms of Photobiomodulation or Low-Level Light Therapy. IEEE J Sel Top Quantum Electron. 2016 May-Jun;22(3):7000417. doi: 10.1109/JSTQE.2016.2561201. PMID: 28070154; PMCID: PMC5215870.

Escudero JSB, Perez MGB, de Oliveira Rosso MP, Buchaim DV, Pomini KT, Campos LMG, Audi M, Buchaim RL. Photobiomodulation therapy (PBMT) in bone repair: A systematic review. Injury. 2019 Nov;50(11):1853-1867. doi: 10.1016/j.injury.2019.09.031. Epub 2019 Sep 21. PMID: 31585673.

EuroQol. EQ-5D value sets: inventory, comparative review and user guide, 2010. EQ-5D value sets The EuroQol Group’s Task Force On Value Sets, 2010.

Garrat AM, Schmidt L, Mackintosh A, Fitzpatrick R. Quality of life measurement: bibliographic study of patient assessed health outcome measures. BMJ 2002; 324(7351):1417-1421

Gracitelli, Mauro Emilio Conforto. Estudo randomizado da osteossíntese das fraturas da extremidade proximal do úmero com placa ou haste intramedular [tese]. São Paulo: , Faculdade de Medicina; 2015 [citado 2023-02-18]. doi:10.11606/T.5.2016.tde-24022016-091653.

Gummesson C, Ward MM, Atroshi I. The shortened disabilities of the arm, shoulder and hand questionnaire (QuickDASH): validity and reliability based on responses within the full-length DASH. BMC Musculoskelet Disord. 2006 May 18;7:44. doi: 10.1186/1471-2474-7-44. PMID: 16709254; PMCID: PMC1513569.

Hamblin MR. Mechanisms and applications of the anti-inflammatory effects of photobiomodulation. AIMS Biophys. 2017;4(3):337-361. doi: 10.3934/biophy.2017.3.337. Epub 2017 May 19. PMID: 28748217; PMCID: PMC5523874.

Handoll HH, Elliott J, Thillemann TM, Aluko P, Brorson S. Interventions for treating proximal humeral fractures in adults. Cochrane Database Syst Rev. 2022 Jun 21;6(6):CD000434. doi: 10.1002/14651858.CD000434.pub5. PMID: 35727196; PMCID: PMC9211385.

Iglesias-Rodríguez, S., Domínguez-Prado, D.M., García-Reza, A. *et al.* Epidemiology of proximal humerus fractures. *J Orthop Surg Res* **16**, 402 (2021).

Jana Neto FC, Martimbianco ALC, Mesquita-Ferrari RA, Bussadori SK, Alves GP, Almeida PVD, Delgado FG, Fonseca LR, Gama MZG, Jorge MD, Hamblin MR, Fernandes KPS. Effects of multiwavelength photobiomodulation for the treatment of traumatic soft tissue injuries associated with bone fractures: A double-blind, randomized controlled clinical trial. J Biophotonics. 2023 Jan 14:e202200299. doi: 10.1002/jbio.202200299. Epub ahead of print. PMID: 36640122.

Jensen MP, Karoly P, Braver S. The measurement of clinical pain intensity: a comparison of six methods. Pain. 1986;27(1):117-26.

Kennedy CA, Beaton DE. A user's survey of the clinical application and content validity of the DASH (Disabilities of the Arm, Shoulder and Hand) outcome measure. J Hand Ther. 2017 Jan-Mar;30(1):30-40.e2. doi: 10.1016/j.jht.2016.06.008. Epub 2016 Jul 26. PMID: 27469538.

Monticone M, Portoghese I, Cazzaniga D, Liquori V, Marongiu G, Capone A, Campagna M, Zatti G. Task-oriented exercises improve disability of working patients with surgically-treated proximal humeral fractures. A randomized controlled trial with one-year follow-up. BMC Musculoskelet Disord. 2021 Mar 20;22(1):293. doi: 10.1186/s12891-021-04140-9. PMID: 33743670; PMCID: PMC7981858.

Neer II CS. Displaced proximal humerus fractures. Part I. Classification and evaluation. J Bone Joint Surg 1970;52A:1077–1089.

Nesioonpour S, Mokmeli S, Vojdani S, Mohtadi A, Akhondzadeh R, Behaeen K, Moosavi S, Hojjati S. The effect of low-level laser on postoperative pain after tibial fracture surgery: a double-blind controlled randomized clinical trial. Anesth Pain Med. 2014 Jun 21;4(3):e17350. doi: 10.5812/aapm.17350. PMID: 25237637; PMCID: PMC4165037.

Neto FCJ, Martimbianco ALC, de Andrade RP, Bussadori SK, Mesquita-Ferrari RA, Fernandes KPS. Effects of photobiomodulation in the treatment of fractures: a systematic review and meta-analysis of randomized clinical trials. Lasers Med Sci. 2020 Apr;35(3):513-522. doi: 10.1007/s10103-019-02779-4. Epub 2019 Apr 13. PMID: 30982176.

Nowak LL, Davis AM, Mamdani M, Beaton D, Schemitsch EH. A concept analysis and overview of outcome measures used for evaluating patients with proximal humerus fractures. Disabil Rehabil. 2021 May;43(10):1450-1462. doi: 10.1080/09638288.2019.1649728. Epub 2019 Sep 3. PMID: 31479302.

Nussbaum EL, Downes L. Reliability of clinical pressurepain algometric measurements obtained on consecutive days. Phys Ther 1998;78:160–169.

Oldrini LM, Feltri P, Albanese J, Marbach F, Filardo G, Candrian C. PHILOS Synthesis for Proximal Humerus Fractures Has High Complications and Reintervention Rates: A Systematic Review and Meta-Analysis. Life (Basel). 2022 Feb 19;12(2):311. doi: 10.3390/life12020311. PMID: 35207598; PMCID: PMC8880552.

Orfale AG, Araújo PM, Ferraz MB, Natour J. Translation into Brazilian Portuguese, cultural adaptation and evaluation of the reliability of the Disabilities of the Arm, Shoulder and Hand Questionnaire. Braz J Med Biol Res. 2005 Feb;38(2):293-302. doi: 10.1590/s0100-879x2005000200018. Epub 2005 Feb 15. PMID: 15785841.

Peluso R, Hesson J, Aikens J, Bullock M. An Update on Physical Therapy Adjuncts in Orthopedics. Arthroplast Today. 2022 Mar 18;14:163-169. doi: 10.1016/j.artd.2022.02.013. PMID: 35330664; PMCID: PMC8938198.

Petros RSB, Ribeiro FR, Tenor AC, Brasil R, Filardi CS, Molin DCD. PROXIMAL HUMERUS FRACTURE WITH LOCKING PLATE: FUNCTIONAL AND RADIOGRAPHIC RESULTS. Acta ortop bras [Internet]. 2019May;27(Acta ortop. bras., 2019 27(3)). Available from: https://doi.org/10.1590/1413-785220192703142049

Rabi S, Evaniew N, Sprague SA, Bhandari M, Slobogean GP. Operative vs non-operative management of displaced proximal humeral fractures in the elderly: A systematic review and meta-analysis of randomized controlled trials. World J Orthop. 2015 Nov 18;6(10):838-46. doi: 10.5312/wjo.v6.i10.838. PMID: 26601066; PMCID: PMC4644872.

Ratajczak K, Szczęsny G, Małdyk P. Comminuted fractures of the proximal humerus - principles of the diagnosis, treatment and rehabilitation. Ortop Traumatol Rehabil. 2019 Apr 30;21(2):77-93. doi: 10.5604/01.3001.0013.1544. PMID: 31180034.

Relvas Silva M, Linhares D, Leite MJ, Nunes B, Torres J, Neves N, Ribeiro Silva M. Proximal humerus fractures: epidemiology and trends in surgical management of hospital-admitted patients in Portugal. JSES Int. 2022 Jan 24;6(3):380-384. doi: 10.1016/j.jseint.2021.12.003. PMID: 35572441; PMCID: PMC9091738.

Richard GJ, Denard PJ, Kaar SG, Bohsali KI, Horneff JG, Carpenter S, Fedorka CJ, Mamelson K, Garrigues GE, Namdari S, Abboud JA, Paxton ES, Kovacevic D, Hebert-Davies J, Ponce BA, King JJ. Outcome measures reported for the management of proximal humeral fractures: a systematic review. J Shoulder Elbow Surg. 2020 Oct;29(10):2175-2184. doi: 10.1016/j.jse.2020.04.006. Epub 2020 Jun 9. PMID: 32951643.

Roux A, Decroocq L, El Batti S, Bonnevialle N, Moineau G, Trojani C, et al. Epidemiology of proximal humerus fractures managed in a trauma center. Orthop Traumatol Surg Res. 2012;98(6):715–9. <https://doi.org/10.1016/j.otsr.2012.05.013>.

Saebo H, Naterstad IF, Bjordal JM, Stausholm MB, Joensen J. Treatment of Distal Radius Fracture During Immobilization with an Orthopedic Cast: A Double- Blinded Randomized Controlled Trial of Photobiomodulation Therapy. Photobiomodul Photomed Laser Surg. 2021 Apr;39(4):280-288. doi: 10.1089/photob.2020.4964. Epub 2021 Mar 19. PMID: 33751924.

Sæbø H, Naterstad IF, Joensen J, Stausholm MB, Bjordal JM. Pain and Disability of Conservatively Treated Distal Radius Fracture: A Triple-Blinded Randomized Placebo-Controlled Trial of Photobiomodulation Therapy. Photobiomodul Photomed Laser Surg. 2022 Jan;40(1):33-41. doi: 10.1089/photob.2021.0125. PMID:

35030040.

Saebo H, Naterstad IF, Stausholm MB, Bjordal JM, Joensen J. Reliability of pain pressure threshold algometry in persons with conservatively managed wrist fractures. Physiother Res Int 2020;25:e1797

Schnackers MLAP, van Horn YY, Meys GHH, Brink PRG, Smeets RJEM, Seelen HAM. Evidence-based rehabilitation therapy following surgery for (peri-)articular fractures: A systematic review. J Rehabil Med. 2019 Oct 4;51(9):638-645. doi: 10.2340/16501977-2599. PMID: 31495902.

Sharma SK, Sardana S, Hamblin MR. Role of opsins and light or heat activated transient receptor potential ion channels in the mechanisms of photobiomodulation and infrared therapy. Journal of Photochemistry and Photobiology (2023), doi: <https://doi.org/10.1016/j.jpap.2023.100160>

Slobogean GP, Noonan VK, O’Brien PJ. The reliability and validity of the Disabilities of Arm, Shoulder, and Hand, EuroQol-5D, Health Utilities Index, and Short Form-6D outcome instruments in patientswith proximal humeral fractures. J Shoulder Elbow Surg 2010;19:342-8. <https://doi.org/10.1016/j.jse.2009.10.021>

Tenor Junior AC, Granja Cavalcanti AM, Albuquerque BM, Ribeiro FR, da Costa MP, Filho RB. Treatment of proximal humeral fractures using anatomical locking plate: correlation of functional and radiographic results. Rev Bras Ortop. 2016 Apr 19;51(3):261-7. doi: 10.1016/j.rboe.2015.08.018. PMID: 27284546; PMCID: PMC4887510.

Thong ISK, Jensen MP, Miro J, Tan G. The validity of pain intensity measures: what do the NRS, VAS, VRS, and FPS-R measure? Scand J Pain. 2018;18(1):99-107.

van de Water AT, Shields N, Davidson M, Evans M, Taylor NF. Reliability and validity of shoulder function outcome measures in people with a proximal humeral fracture. Disabil Rehabil 2014;36: 1072-9. <https://doi.org/10.3109/09638288.2013.829529>

Ware JE, Sherbourne CD. The MOS 36-item short health survey (SF-36). I. Conceptual framework and item selection. Med Care 1992; 30(6):473-483.
