# Supplementary material for: The role of photobiomodulation in the functional recovery of proximal humerus fractures: a randomized controlled clinical protocol
Source: PLoS One. 2025 Apr 29;20(4):e0321746. doi: 10.1371/journal.pone.0321746 (PMC12040229; doi:10.1371/journal.pone.0321746)
Supplement: S5 Appendix — (DOCX) [file pone.0321746.s005.docx]

**PROGRAMA DE PÓS-GRADUAÇÃO EM BIOFOTÔNICA APLICADA ÀS CIÊNCIAS DA SAÚDE**

**LUIZ CLÁUDIO DE FREITAS**

**EFEITOS DA FOTOBIOMODULAÇÃO NA RECUPERAÇÃO FUNCIONAL DE FRATURAS DO ÚMERO PROXIMAL: ESTUDO CLÍNICO CONTROLADO RANDOMIZADO DUPLO CEGO**

**São Paulo, SP**

**2023**

**UNIVERSIDADE NOVE DE JULHO**

**PROGRAMA DE PÓS-GRADUAÇÃO EM BIOFOTÔNICA APLICADA ÀS CIÊNCIAS DA SAÚDE**

**LUIZ CLÁUDIO DE FREITAS**

**EFEITOS DA FOTOBIOMODULAÇÃO NA RECUPERAÇÃO FUNCIONAL DE FRATURAS DO UMERO PROXIMAL: ESTUDO CLÍNICO CONTROLADO RANDOMIZADO DUPLO CEGO**

Projeto de pesquisa apresentado ao

Comitê de ética em Pesquisa da Universidade Nove de Julho

**São Paulo, SP**

**2023**

**RESUMO**

Dentre as várias complicações da evolução pós-operatória das fraturas do úmero proximal (FUP) estão a dor e a rigidez articular, gerando limitação funcional importante no membro afetado. A fisioterapia é o tratamento padrão tanto para os casos cirúrgicos quanto os não cirúrgicos. Estudos tem demonstrado efeitos positivos da fotobiomodulação (FBM) na reparação e regeneração das fraturas, bem como na analgesia e melhora funcional. No entanto, sugerem padronização e evidências adicionais. O presente estudo clínico duplo cego controlado randomizado terá como objetivo avaliar os efeitos da FBM na recuperação funcional de participantes com FUP tratadas cirurgicamente com placas bloqueadas especiais. Os 42 participantes serão randomizados (1:1) em 2 grupos, sendo grupo Controle (tratamento padronizado de fisioterapia associada a FBM simulada) e grupo FBM (tratamento padronizado de fisioterapia associada a FBM ativa). A FBM será aplicada pelo próprio participante em seu domicílio todos os dias, por 10 minutos, com uso de um dispositivo contendo 318 LEDs *light emitting diodes*, sendo 159 LEDs de 660 nm (28,5 mW; 12 J/cm^2^; 17 J por LED) e 159 LEDs de 850 nm (23 mW; 10 J/cm^2^; 14 J por LED). As sessões de FBM e as de fisioterapia (30 minutos, 2 vezes por semana) serão realizadas por 12 semanas. Os participantes não terão conhecimento de sua alocação e serão avaliados em 24h, 1, 2, 4, 8 e 12 semanas após o procedimento cirúrgico por 4 examinadores também cegos em relação a alocação de cada participante. O desfecho principal, avaliado em todos os períodos experimentais, será a recuperação da função do ombro utilizando a escala funcional Quick-DASH. Os desfechos secundários serão as avaliações de amplitude dos movimentos do ombro com goniômetro digital, qualidade de vida com uso do questionário SF-6 e ocorrência de efeitos adversos em todos os períodos experimentais. Já a dor espontânea, à pressão (dolorímetro), noturna e o uso de analgésicos serão avaliados em 1, 2, 4, 8 e 12 semanas; a consolidação das fraturas em 4, 8 e 12 semanas por meio de exames radiográficos; e a força muscular por sustentação progressiva de halteres em 8 e 12 semanas. Além das avaliações pontuais, cada participante será acompanhado diariamente por meio de contato telefônico. Os dados coletados serão armazenados, organizados em repositório e serão aplicados os testes estatísticos apropriados para cada análise específica. Em todos os testes, será adotado o nível de significância de 5%.

**Palavras Chaves:** Fotobiomodulação, Fratura do Úmero, Quick-DASH, Qualidade de vida, Fototerapia

**ABSTRACT**

Among the various complications of the postoperative evolution of fractures of the proximal humerus (FUP) are pain and joint stiffness, generating significant functional limitation in the affected limb. Physical therapy is the standard treatment for both surgical and non-surgical cases. Studies have shown positive effects of photobiomodulation (FBM) on repair and regeneration of fractures, as well as on analgesia and functional improvement. However, they suggest standardization and additional evidence. This randomized controlled double-blind clinical study will aim to evaluate the effects of FBM on the functional recovery of participants with FUP surgically treated with special locking plates. The 42 participants will be randomized (1:1) into 2 groups, the Control group (standardized physiotherapy treatment associated with simulated FBM) and the FBM group (standardized physiotherapy treatment associated with active FBM). The FBM will be applied by the participant at home every day, for 10 minutes, using a device containing 318 LEDs light emitting diodes, 159 LEDs of 660 nm (28.5 mW; 12 J/cm2; 17 J per LED) and 159 850 nm LEDs (23 mW; 10 J/cm2; 14 J per LED). The FBM and physiotherapy sessions (30 minutes, 2 times a week) will be held for 12 weeks. Participants will not be aware of their allocation and will be assessed at 24h, 1, 2, 4, 8 and 12 weeks after the surgical procedure by 4 examiners who are also blinded to each participant's allocation. The main outcome, evaluated in all experimental periods, will be the recovery of shoulder function using the Quick-DASH functional scale. Secondary outcomes will be assessments of range of motion of the shoulder using a digital goniometer, quality of life using the SF-6 questionnaire and the occurrence of adverse effects in all experimental periods. Spontaneous pain, pressure pain (dolorimeter), nocturnal pain and the use of analgesics will be evaluated at 1, 2, 4, 8 and 12 weeks; consolidation of fractures at 4, 8 and 12 weeks through radiographic examinations; and muscle strength by progressive weight bearing at 8 and 12 weeks. In addition to punctual evaluations, each participant will be monitored daily through telephone contact. The collected data will be stored, organized in a repository and the appropriate statistical tests will be applied for each specific analysis. In all tests, a significance level of 5% will be adopted.

Keywords: Photobiomodulation, Humeral Fracture, Quick-DASH, Quality of life, Phototherapy

**LISTA DE TABELAS**

| Tabela 1. Ensaios clínicos randomizados controlados sobre FBM em fraturas ósseas…. | 32 |
| --- | --- |
| Tabela 2. Parâmetros dosimétricos do dispositivo de FBM............................................. | 32 |
| Tabela 3. Custos do tratamento ..................................................................................... | 38 |
|  |  |
|  |  |
|  |  |
|  |  |
|  |  |
|  |  |
|  |  |
|  |  |
|  |  |

**LISTA DE ABREVIATURAS E SIGLAS**

| ADM | ....... | Amplitude De Movimento |
| --- | --- | --- |
| AMP | ....... | do inglês *Adenosine Monophosphate* |
| AO | ....... | do alemão *Arbeitsgemeinschaft fur Osteosynthesefragen* |
| ASES | ....... | do inglês *American Shoulder and Elbow Surgeon’s Score* |
| ATP | ....... | do inglês *Adenosine Triphosphate* |
| CEP | ....... | Comitê em Ética e Pesquisa |
| CONSORT | ....... | do inglês *Consolidated Standards of Reporting Trials* |
| DASH | ....... | do inglês *Disabilities of the Arm, Shoulder and Hand* |
| ECR | ....... | Estudo Clínico Randomizado |
| EQ-5D | ....... | do inglês *Euroqol-5Dimension* |
| EROS | ....... | Espécies Reativas de Oxigênio |
| EVA | ....... | Escala Visual Analógica |
| FBM | ....... | Fotobiomodulação |
| FUP | ....... | Fraturas do Úmero Proximal |
| HMACN | ....... | Hospital Municipal Alípio Correa Netto |
| ICC | ....... | Coeficiente de Correlação Intraclasse |
| LEDs | ....... | do inglês *Light Emitting Diodes* |
| OTA | ....... | do inglês *Orthopaedic Trauma Association* |
| PRO | ....... | do inglês *Patient Reported Outcomes* |
| ProFHER | ....... | do inglês *Proximal Fracture of Humerus: Evaluation by Randomisation* |
| RAFI | ....... | Redução Aberta e Fixação Interna |
| SF36 | ....... | do inglês *36-Item Short-Form Health Survey* |
| SF6D | ....... | do inglês *Short-Form 6 dimension* |
| SPIRIT | ....... | do inglês *Standard Protocol Items: Recommendations for Interventional Trials* |
| TCLE | ....... | Termo de Consentimento Livre e Esclarecido |
| TRP | ....... | do inglês *Transient Receptor Potential* |

# **CONTEXTUALIZAÇÃO**

As fraturas do úmero proximal (FUP) são as que ocorrem no colo cirúrgico (estreitamento logo abaixo das tuberosidades maior e menor do úmero) ou nas regiões proximais a ele. Sua incidência varia de acordo com a região geográfica e a data do levantamento de dados, porém de maneira geral, representam de 5 a 10% de todos os tipos de fraturas ósseas do corpo (Court-Brown & Cesar 2006, Relvas Silva et al 2021, Iglesias-Rodríguez et al 2021). A incidência de FUP tende a subir com o avançar da idade, sendo apontada como a terceira fratura mais frequente na população acima de 65 anos de idade a depender do estado de saúde, grau de atividade e do cuidado recebido (Roux et al 2012, Relvas Silva et al 2021, Iglesias-Rodríguez et al 2021).

Em idosos a maioria das FUP está relacionada à traumas de baixa energia, principalmente devido a quedas da própria altura ou trauma direto na face lateral do ombro. Nestes casos, as mulheres são as mais atingidas pela maior expectativa de vida e pela osteoporose (Barbosa et al 2008, Iglesias-Rodríguez et al 2021). Nos indivíduos abaixo de 55 anos, os traumas que geram as FUP tendem a ser de maior energia e sua distribuição não é tão afetada pelo gênero (Iglesias-Rodríguez et al 2021).

### ***Classificação***

As principais classificações utilizadas para fraturas do úmero proximal são a de Neer e AO/OTA (*Arbeitsgemeinschaft fur Osteosynthesefragen* /*Orthopaedic Trauma Association)*. A classificação de Neer (1970) definiu os 4 fragmentos da fratura: cabeça, tuberosidades maior e menor e diáfise. As fraturas são consideradas com desvio quando os fragmentos estão transladados ao menos 1 cm ou angulados no mínimo 45°. Assim as fraturas podem ser classificadas como não desviadas e desviadas/deslocadas em 2, 3 ou 4 partes, como segue (Carrerra et al 2012, Petros et al 2019):

Grupo I: fratura minimamente desviada (desvio menor que 1cm ou angulação menor que 45º.

Grupo II: fratura com desvio (maior que 1cm ou angulação maior que 45º). Fragmento = colo anatômico do úmero proximal.

Grupo III: fratura com desvio (maior que 1cm ou angulação maior que 45º).

Fragmento = colo cirúrgico do úmero proximal.

Grupo IV: fratura com desvio do tubérculo maior do úmero (maior que 1cm ou angulação maior que 45º).

2 partes: (sem desvio do colo cirúrgico);

3 partes: (com desvio do colo cirúrgico);

4 partes: (com desvio do colo cirúrgico e tubérculo menor).

Grupo V: fratura com desvio do tubérculo menor (maior que 1cm ou angulação maior que 45º).

2 partes: (sem desvio do colo cirúrgico);

3 partes: (com desvio do colo cirúrgico);

4 partes: (com desvio do colo cirúrgico e tubérculo menor).

Grupo VI: fratura associada à luxação glenoumeral e que também se subdividem em duas, três ou quatro partes.

A classificação AO/OTA consiste em estrutura alfa numérica baseada em exames de imagem (radiografia, tomografia e ressonância magnética quando necessário). Cada osso é definido por um número, no caso do úmero, número 1. A localização da fratura no osso também é definida por números, sendo o segmento proximal (1), diáfise (2) e segmento distal (3), de acordo com o sistema de quadrados de Heim. Quanto ao tipo, extra articular unifocal ou duas partes (A), extra articular bifocal ou três partes (B) e articular ou quatro partes (C), (Carrera et al 2012).

**11- Região proximal do úmero**

A-Extra articular unifocal

A1: tuberosidades

A1.1 maior

A1.2 menor

A2- colo cirúrgico

A2.1 simples

A2.2 cunha

A2.3 multifragmentada

-A3 vertical

**11B- Extra articular, bifocal**

B1 colo cirúrgico

B1.1 com tuberosidade maior

B2.2 com tuberosidade menor

**11C- Articular ou 4 partes**

C1 colo anatômico

C1.1 impactada em valgo

n- tuberosidade maior

o -Tuberosidade menor

p -ambas

C1.2- colo isolado

### ***Tratamento***

O tratamento destas fraturas ainda gera controvérsias e pode ser tecnicamente desafiador, sendo associado a uma carga significativa de recursos e cuidados a depender de sua complexidade, da qualidade óssea, demanda funcional, idade e comorbidades do paciente (Ziegler et al 2020, Iglesias-Rodríguez et al 2021, Relvas Silva et al 2021, Handoll et al 2022).

Uma revisão sistemática recente, buscou as melhores maneiras de restaurar a função do ombro e evitar efeitos prejudiciais do tratamento avaliando 47 estudos (3.179 pessoas com fratura de ombro, maioria mulheres com 60 anos ou mais) realizados em 21 países, sendo que a maioria deles seguiu os participantes por pelo menos um ano. Os autores concluíram que ainda não existe evidência científica suficiente, por meio de ensaios clínicos controlados e randomizados, para basear as escolhas entre tratamento conservador e as diferentes modalidades cirúrgicas no tratamento destas fraturas (Handoll et al 2022). Mesmo em relação ao tratamento conservador, não foram encontradas evidências suficientes para concluir que 1 semana de repouso com imobilização seja melhor ou pior que 3 ou mais semanas (Handoll et al 2022).

Na prática clínica, normalmente as fraturas não desviadas ou minimamente desviadas e estáveis são elegíveis para tratamento conservador, com uso de tipoia por uma ou mais semanas e reabilitação. Já as desviadas, instáveis, abertas ou associadas a lesão vascular em geral são tratadas cirurgicamente (Ratajczack et al 2019, Petros et al 2019, George et al 2021, Handoll et al 2022).

O tratamento cirúrgico envolve diversas técnicas de estabilização como fixação com pinos, hastes, placas ou artroplastia. Uma revisão sistemática recente comparou os resultados das técnicas na evolução de pacientes portadores de FUP com desvio sem encontrar diferenças estatísticas entre elas e nem entre elas e o tratamento conservador. Os achados das comparações mostraram as tendências descritas a seguir. A hemiartroplastia evidenciou melhores resultados na qualidade de vida. A artroplastia reversa mostrou melhores resultados na abdução, flexão, escala de Constant, taxas mais baixas de consolidação viciosa e osteonecrose e necessidade de reintervenção cirúrgica. Já a redução aberta acompanhada de fixação interna (RAFI) mostrou melhores resultados no escore de desabilidade Quick-DASH e na escala visual analógica dor (Davey et al 2022).

A RAFI acompanhada do uso de placa bloqueada tem o objetivo de preservar a vascularização, estabilizando o(s) fragmento(s) de modo suficiente para mobilização precoce apresentando menor índice de perda de redução (Oldrini et al 2022, Petros et al 2019). Em série de 33 pacientes procedidos com placa bloqueada com estabilidade angular devido fraturas do úmero proximal, Monteiro et al 2011, obtiveram 91,2% de ótimos e bons resultados no escore funcional UCLA (*University of Califórnia at Los Angeles*), considerando este método eficiente na estabilização de FUP, sem mais complicações.

Ockert et al 2014 relataram a evolução de 43 casos de FUP desviadas e instáveis segundo critérios de Neer e AO/OTA, procedidas com placa bloqueada. Após 10 anos do tratamento cirúrgico, a maioria dos pacientes apresentaram excelentes e bons resultados. Os casos de pobre resultado a longo prazo foram de pacientes idosos e do gênero feminino (Ockert et al 2014).

George PK et al 2021, em série de 35 casos procedidos com placa bloqueada de ângulo fixo, concluíram que a manutenção de estabilidade efetiva obtida no ato cirúrgico mantida durante o seguimento ambulatorial, mobilização precoce se torna possível o que permite ao paciente atingir melhor ADM e retorno mais rápido às atividades. Perda de redução foi raramente vista comparada a outros implantes.

Outra revisão sistemática, do mesmo ano, buscou as melhores maneiras de restaurar a função do ombro e evitar efeitos prejudiciais do tratamento avaliando 47 estudos (3.179 pessoas com fratura de ombro, maioria mulheres com 60 anos ou mais) realizados em 21 países, sendo que a maioria deles seguiu os participantes por pelo menos um ano. Os autores concluíram que ainda não existe evidência científica suficiente, por meio de ensaios clínicos controlados e randomizados, para basear as escolhas entre tratamento conservador e as diferentes modalidades cirúrgicas no tratamento destas fraturas (Handoll et al 2022). Mesmo em relação ao tratamento conservador, não foram encontradas evidências suficientes para concluir que 1 semana de repouso com imobilização seja melhor ou pior que 3 ou mais semanas (Handoll et al 2022).

### ***Reabilitação***

Após uma fratura de membro superior, frequentemente pacientes são referenciados à fisioterapia para reabilitação visando redução de dor, melhora do arco de movimento e ganho progressivo de força muscular tantos nos casos cirúrgicos como nos conservadores. Embora com claros objetivos, as intervenções utilizadas variam enormemente, podendo incluir modalidades térmicas, recursos eletrofísicos, terapia manual, cinesioterapia, aplicação de órteses dinâmicas, orientações e educação (Peluso et al 2022).

Os protocolos de reabilitação após FUP também não estão bem definidos na literatura. Uma revisão sistemática recente (Schnackers et al 2019) evidenciou a escassez de protocolos de fisioterapia detalhados e baseados em evidências científicas, que visem a devolução das atividades da vida diária dos pacientes e indicou que dentre os estudos avaliados, o ensaio clínico ProFHER (*PROximal Fracture of the Humerus Evaluation by Randomisation)* publicado em 2015 (Handoll et al 2015) era o único a apresentar uma descrição completa neste sentido.

O protocolo de fisioterapia ProFHER apresenta progressão em fases. O tempo da evolução das fases depende de fatores diversos: idade, estágio de consolidação da fratura, tolerância à dor, expectativa do paciente, nível de atividade e condições gerais de saúde. Caso a fratura esteja estável, a fase 3 é iniciada após retirada da tipoia com 3 semanas e a fase 4, iniciada 3 semanas mais tarde. Assim a maioria dos pacientes retornará ao estado funcional em 6 semanas, quando a função independente do ombro é atingida.

Em 2019 Ratajczak et al ressaltaram que o tratamento ideal da FUP depende do tipo de fratura, das expectativas do paciente, do nível de condicionamento físico e da aceitação do tratamento proposto. Deste modo, recomendaram que os exercícios devem ser ajustados conforme a estabilidade da fratura bem como as habilidades e expectativas do paciente. Em casos cirúrgicos, indicam que é importante iniciar a reabilitação imediatamente após remoção da imobilização, que seria entre a quarta e nona semanas. A reabilitação, segundo os autores, deve incluir exercícios passivos, exercícios passivo-ativos e isométricos ativos, concêntricos e excêntricos conforme a função do membro for melhorando, de maneira monitorada e cuidadosa. Os autores sugeriram a reabilitação pós-operatória, por meio de cinesioterapia, em 3 estágios:

Estágio 1: primeiras 3 semanas

- Imobilização com tipoia no membro operado sem sustentação de peso (exceto para artroplastia);
- Iniciação de exercícios pendulares;
- Movimentos passivos assistidos;
- Evitar rotação externa nas primeiras 6 semanas.

Estágio 2: terceira a nona semana, desde que não haja anormalidades na consolidação ou desvio secundário dos fragmentos.

- Exercícios ativos assistidos, flexão e abdução da articulação do ombro
- Exercícios ativos cuidadosos com abdução do membro até que dor ocorra, sem carga nas primeiras 6 semanas. Acrescentar carga gradualmente.

Estágio 3: após a 9 semana

- Exercícios isotônicos, excêntricos e concêntricos;
- Em pacientes que apresentarem consolidação radiográfica da fratura e contratura articular, são indicados exercícios passivos de alongamento com profissional experiente.

Já Monticone et al (2021) compararam a eficácia de exercícios orientados para conclusão de tarefas baseados em atividades de trabalho específicas dos pacientes e terapia ocupacional aos exercícios convencionais e encontraram resultados superiores na melhoria da incapacidade, dor e qualidade de vida em pacientes após cirurgia para tratamento de FUP. Após mobilização precoce (no final da primeira semana após a cirurgia), foram introduzidos exercícios básicos para melhorar a mobilidade glenoumeral e a consciência muscular do membro superior. Os pacientes aprenderam técnicas para que progressivamente ganhassem resistência, velocidade, potência e complexidade nos movimentos padrão dos músculos envolvidos na lesão. Depois também foram introduzidos exercícios baseados nas atividades de trabalho específicas dos pacientes que incluíram mover objetos de diferentes formas e tamanhos em direções diferentes, pegar objetos de uma mesa em diferentes posições, montar objetos complexos misturando componentes dispostos em 1 mesa, pegar objetos arremessados em diferentes alturas e velocidades. Exercícios adicionais foram introduzidos com o objetivo de recuperar destreza, equilíbrio e outras demandas funcionais. Eles incluíram: passar de um sofá para a posição sentado e de uma cadeira para a posição em pé; andar e girar na velocidade preferida; descer e subir escadas e obstáculos. Os terapeutas ocupacionais também orientaram sobre os cuidados com a tipoia e sobre princípios ergonômicos baseados nas atividades que os pacientes executavam antes da fratura.

Como citado anteriormente, a reabilitação após fraturas pode envolver também o uso de recursos eletrofísicos, dentre eles as fontes de luz.

### ***Fotobiomodulação***

A fotobiomodulação (FBM), anteriormente denominada terapia com laser de baixa intensidade, pode ser definida como o uso de fontes de luz para estimular a cura, aliviar a dor e reduzir a inflamação (Hamblin 2017). Esta terapia é segura, minimamente invasiva e não provoca efeitos colaterais (Hamblin 2017, Sharma et al 2023). As fontes mais usadas para FBM são os lasers e LEDs (*light emitting diodes*) em baixa intensidade ou de baixa potência nos comprimentos de onda vermelho (600–700 nm) e infravermelho próximo (770–1200 nm).

A ação da FBM é baseada na absorção da luz por cromóforos celulares. Os cromóforos já descritos podem ser agrupados em 3 categorias: os citocromos mitocondriais (proteínas ligadas membrana interior das mitocôndrias, como a citocromo C oxidase); as opsinas (3 e 4) e os canais de íons de cálcio sensíveis ao calor ou à luz; e os aglomerados de água nanoestruturados (Sharma et al 2023). Canais de íons sensíveis à luz são ativados pela absorção de luz pelas opsinas enquanto os citocromos mitocondriais absorvem luz nos comprimentos de onda vermelho e infravermelho próximo. Luz em comprimentos de onda acima de 980 nm é provavelmente absorvida por aglomerados de água nanoestruturados e ativam canais iônicos receptores de potencial transitório (do inglês *transient receptor potential*, TRP). Já os TRP sensíveis ao calor podem ser ativados por alterações de temperatura, mesmo que discretas (Sharma et al 2023).

A luz vermelha ou infravermelha quando absorvida pela citocromo C oxidase mitocondrial possivelmente dissocia o óxido nítrico inibitório a ela associado levando a um aumento na sua atividade enzimática, no transporte de elétrons, no potencial de membrana mitocondrial e na produção de ATP (Sharma et al 2023). Outra possível explicação para os efeitos da FBM é que canais de íons sensíveis à luz seriam ativados permitindo a entrada de cálcio nas células ou nas mitocôndrias. Ambas as vias seriam capazes então de ativar o metabolismo mitocondrial e muitas vias de sinalização como as espécies reativas de oxigênio (ROS), AMP cíclico, óxido nítrico e íons de cálcio, levando à ativação de fatores de transcrição responsáveis por aumentar a expressão de genes relacionados com a síntese proteica, migração celular, proliferação, sinalização anti-inflamatória, proteínas anti-apoptóticas e enzimas antioxidantes (de Freitas & Hamblin 2016, Sharma et al 2023).

Com relação ao uso da FBM no tratamento de fraturas de membros, uma revisão sistemática com metanálise, publicada em 2020, evidenciou a falta de ensaios controlados randomizados (ECR) sobre o tema incluindo somente 2 estudos com baixo nível de evidência. Na análise conjunta destes ECRs foi evidenciada diferença clínica e estatisticamente significativa, a favor na FBM, na melhoria da função do membro fraturado e redução estatisticamente significativa na dor, porém ausência de diferença em relação à cicatrização radiográfica da fratura (Neto et al 2020). A revisão concluiu ainda que há necessidade de novos ECRs que sigam as recomendações do CONSORT (do inglês, *Consolidated Standards of Reporting Trials*) e busquem a melhor combinação de parâmetros dosimétricos e a avaliação da ocorrência de possíveis efeitos adversos. Outras revisões, que avaliaram o papel da FBM no reparo ósseo usando como base estudos com diversas metodologias concluíram que a FBM pode acelerar a regeneração óssea, mas que há necessidade de buscar padronizar parâmetros e protocolos de tratamento (Deana et al 2018, Escudero et al 2019, Cheng et al 2020).

Posteriormente, Saebo et al (2021) avaliaram o efeito da FBM, aplicada durante o período de imobilização com gesso (tratamento conservador, não cirúrgico) na dor e rigidez em pacientes com fraturas do rádio distal. Em ECR duplo cego, 53 participantes receberam 9 aplicações de FBM (904 nm, 25W - pico de pulso, 60 kHz, 60 mW de potência média, 6.6 J por sessão) e foram avaliados após 4, 8, 12 e 26 semanas após o trauma. O grupo tratado com FBM exibiu melhores resultados que o placebo nas avaliações de amplitude de movimento e força de preensão em todo período de avaliação sendo significativa estatisticamente após 4 semanas. As diferenças entre os punhos lesionados e não lesionados de cada paciente foram significativamente menores no grupo FBM em relação à força de preensão e de pinça nas semanas 4 e 26. Além disso, um número significativamente menor de pacientes reportou dor noturna na terceira semana de acompanhamento no grupo FBM quando comparado ao grupo placebo. Porém, não houve diferença entre o grupo tratado com FBM e o placebo com relação ao questionário padronizado de avaliação de função e percepção de dor no punho pelo paciente e em relação ao consumo de analgésicos e edema local.

O mesmo grupo de autores avaliou o efeito da FBM (904 nm, 25W - pico de pulso, 60 kHz, 60 mW de potência média, 7,2 J por sessão sendo 1,2J por ponto), associada a exercícios, aplicada após remoção da imobilização em 50 pacientes com fraturas do rádio distal tratadas de maneira conservadora (não procedidas cirurgicamente). O ECR triplo cego avaliou questionário padronizado de avaliação de função e percepção de dor no pulso pelo paciente, dor noturna e consumo de analgésicos após 4, 8, 12, e 26 semanas. A dor noturna e o consumo de analgésicos foram avaliados também após 7 semanas. Foram encontrados resultados significativamente melhores no grupo FBM, no questionário padronizado de avaliação de função e na percepção de dor no pulso nos períodos de 8, 12 e 26 semanas. A dor noturna e o consumo de analgésicos foram significativamente menores no grupo FBM nos períodos entre 7 e 26 semanas (Saebo et al 2022).

Já Jana Neto et al (2023) avaliaram o feito da FBM com LEDs no processo de cicatrização de lesões de partes moles associadas a fratura de tíbia procedidas cirurgicamente. Foram incluídos 27 indivíduos adultos que aguardavam resolução das lesões de partes moles para serem submetidos à cirurgia definitiva. A FBM (144 diodos emissores de LED nos comprimentos de onda de 420nm, 660nm e 850nm, 3J por ponto, por 10 minutos) foi usada diariamente até que os tecidos moles apresentassem condições para a cirurgia definitiva. O grupo controle foi tratado com equipamentos com características externas idênticas, mas inativos. O grupo tratado com FBM mostrou melhor média diária de cicatrização, maior alteração média diária na redução do escore de dor, quatro vezes menos ocorrência de infecção no local dos pinos do fixador externo e resolução mais rápida (10 dias a menos) além de não terem sido relatados efeitos adversos.

### ***Avaliação do sucesso terapêutico no tratamento das FUP***

Mesmo após tratamento cirúrgico ou conservador, a FUP pode continuar gerando quadro de dor, limitações para atividades da vida diária, incapacidade no membro e redução na qualidade de vida. Neste sentido, tem sido observado que os ensaios clínicos sobre FUP refletem diferentes desfechos dificultando a comparação dos efeitos dos tratamentos e o estabelecimento de um consenso (Nowak et al 2019, Richard et al 2020).

Deste modo, tem sido sugerido que a avaliação do sucesso terapêutico do tratamento das FUP reflita primordialmente o reestabelecimento das atividades diárias dos pacientes englobando ferramentas chamadas em inglês de *Patient Reported Outcomes* (PRO). Deste modo, para o acompanhamento das FUP é indicada a inclusão de pelo menos um escore funcional (como o DASH - *Disabilities of the Arm, Shoulder and Hand*, ASES - *American Shoulder and Elbow Surgeon’s* *Score* ou *Oxford Shoulder Score)* e PROs que reflitam o estado de saúde e a qualidade de vida por meio de ferramentas como o EQ-5D - *EuroQol-5 Dimension* e o SF6D - *Short Form-6 Dimension* (Nowak et al 2019, Richard et al 2020).

O índice de incapacidade funcional DASH foi desenvolvido em 1996 para ser um instrumento de mensuração, autoadministrado, sobre os sintomas e estado físico funcional dos membros superiores podendo ser usado para fins clínicos e científicos. Ele é composto por perguntas relacionadas às atividades diárias, sintomas e função social/papel na última semana. As 30 perguntas podem ser respondidas por itens que valem de 1 a 5 pontos, totalizando 100 pontos, sendo que 100 corresponde a dificuldades/incapacidades significativas e 0 à ausência de dificuldades (Hudak et al 1996). Este instrumento já foi adaptado para mais de 50 línguas e apresenta quantidade considerável de validação em inúmeros artigos científicos (Kennedy & Beaton 2017, Richard et al 2020). O DASH apresentou forte confiabilidade, validade moderadamente forte e altas propriedades psicométricas na avaliação de pacientes com FUP (Slobogean et al 2010, van de Wateret al 2014).

Uma versão mais curta do DASH foi lançada em 2005 com o nome de Quick DASH e contém 11 itens (Beaton et al 2005). Assim como a versão original, a versão mais curta oferece 5 opções de resposta e tem valor total de 100 pontos. Os dois questionários têm módulos opcionais, que são pontuados separadamente, e são destinados à atletas/artistas performáticos e para o trabalho (Kennedy & Beaton 2017). O QuickDASH pode ser utilizado no lugar do DASH com precisão em distúrbios da extremidade superior sendo um instrumento mais fácil de aplicar e que consome menos tempo (Beaton et al 2005, Gummesson et al 2006).

A versão brasileira do DASH foi validada em 2006 (Orfale et al 2006), já o Quick DASH foi validado mais recentemente (da Silva et al 2020) se mostrando confiável, responsivo e podendo ser utilizado como alternativa ao DASH para avaliar indivíduos com disfunções da extremidade superior relacionadas a lesões ortopédicas e traumáticas.

O EQ-5D e o SF6D mostraram forte confiabilidade e moderadamente forte validade em pacientes com FUP, sendo que o EQ5D também mostrou boa capacidade de resposta e foi recomendado como ferramenta de avaliação de Qualidade de Vida Relacionada à Saúde para pacientes com FUP em revisão sistemática (Olerud et al 2011, Rabi et al 2015, Richard et al 2020).

O EQ-5D-3L contempla cinco domínios de saúde (mobilidade, autocuidado, atividades usuais, dor/desconforto, ansiedade/depressão) com três níveis cada um (sem problemas, alguns problemas e problemas extremos). Além disso, ele contém uma escala análogo-visual (EAV) onde o participante atribui um valor para o seu próprio estado de saúde que varia de zero (“Pior estado de saúde possível”) a 100 (“Melhor estado de saúde possível”) o que pode gerar um total de 243 estados de saúde (EuroQol, 2010).

O instrumento SF-6 deriva da versão adaptada para uso no Brasil do questionário SF-36 (*Medical Outcomes Study 36-Item Short-Form Health Survey*) que é um questionário avaliado em mais de 200 doenças e traduzido em 40 países (Ware & Sherbourne 1992, Garrat et al 2002, Campolina et al 2011).

# **JUSTIFICATIVA**

Muitas evidências mostram a capacidade da FBM em atuar no controle da dor e auxiliar no reparo tecidual. Porém no tratamento de fraturas, existe a necessidade de desenvolver ECRs, com grande rigor metodológico, para estabelecer os melhores parâmetros e resultados da FBM (Deana et al 2018, Escudero et al 2019, Cheng et al 2020, Neto et al 2020, Saebo et al 2021, Saebo et al 2022). No caso da FUP, além da importância de encontrar os melhores parâmetros dosimétricos para a aplicação da FBM, também é apontada a necessidade de padronizar os desfechos a serem avaliados (Richard et al 2020, Nowak et al 2019) de modo a obter um padrão ouro de tratamento pois a grande maioria dos casos apresenta período de recuperação lento acompanhado de dor e limitação de função. Este estudo avaliará os efeitos da FBM (usando um dispositivo LED de uso domiciliar com parâmetros apoiados na literatura) principalmente sobre a recuperação funcional no pós-operatório de fraturas proximais do úmero tratadas cirurgicamente por meio de um estudo clínico controlado, randomizado e duplo cego.

**Hipóteses**

H0 = A fotobiomodulação não é efetiva na recuperação funcional da FUP.

H1 = A fotobiomodulação é efetiva na recuperação funcional da FUP.

# **OBJETIVOS**

O objetivo geral deste estudo será avaliar os efeitos da fotobiomodulação na recuperação funcional de fraturas de úmero proximal, procedidas com RAFI (Redução Aberta e Fixação Interna) e estabilizadas com placa de ângulo fixo.

### **3.1. Objetivo principal**

O desfecho principal do estudo será a avaliação da recuperação funcional após fraturas de úmero proximal, procedidas com RAFI, estabilizadas com placa de ângulo fixo e tratadas com fisioterapia e fotobiomodulação, por meio da versão brasileira do questionário Quick DASH (*Disabilities of the Arm, Shoulder and Hand*).

### **3.2. Objetivos secundários**

Os desfechos secundários serão avaliar os efeitos da FBM aplicada após fraturas de úmero proximal, procedidas com RAFI, estabilizadas com placa de ângulo fixo e tratadas com fisioterapia sobre:

- A amplitude dos movimentos (ADM) do ombro avaliada de maneira temporal e em comparação com o membro não afetado
- A força muscular mensurada de maneira temporal e em comparação com o membro não afetado
- A intensidade da dor espontânea e durante a função do membro
- A ocorrência de dor noturna
- A dor à pressão no local da fratura
- O consumo de analgésicos
- A consolidação da fratura
- A qualidade de vida
- A incidência de eventos adversos
- Os custos diretos e indiretos relacionados aos procedimentos realizados no período da pesquisa para posterior cálculo da relação custo efetividade da intervenção

# **MATERIAL E MÉTODOS**

Trata-se de um ensaio clínico controlado, randomizado paralelo e duplo cego. O delineamento seguirá as recomendações internacionais para ensaios clínicos randomizados do protocolo SPIRIT (*Standard Protocol Items: Recommendations for Interventional Trials* - Chan et al 2013), e as normas regulamentadoras de pesquisa em seres humanos do Comitê Nacional de Ética em Pesquisa (CONEP).

Figura - Fluxograma do estudo nas normas Spirit

Este projeto de pesquisa será submetido ao Comitê de Ética em Pesquisa da Universidade Nove de Julho (UNINOVE) e os sujeitos assinarão o termo de consentimento livre após esclarecimentos (APÊNDICE 1). O protocolo será registrado na plataforma Clinical Trials (<https://clinicaltrials.gov/>). O relato do estudo será conduzido de acordo com a diretriz CONSORT (Consolidated Standards of Reporting Trials).


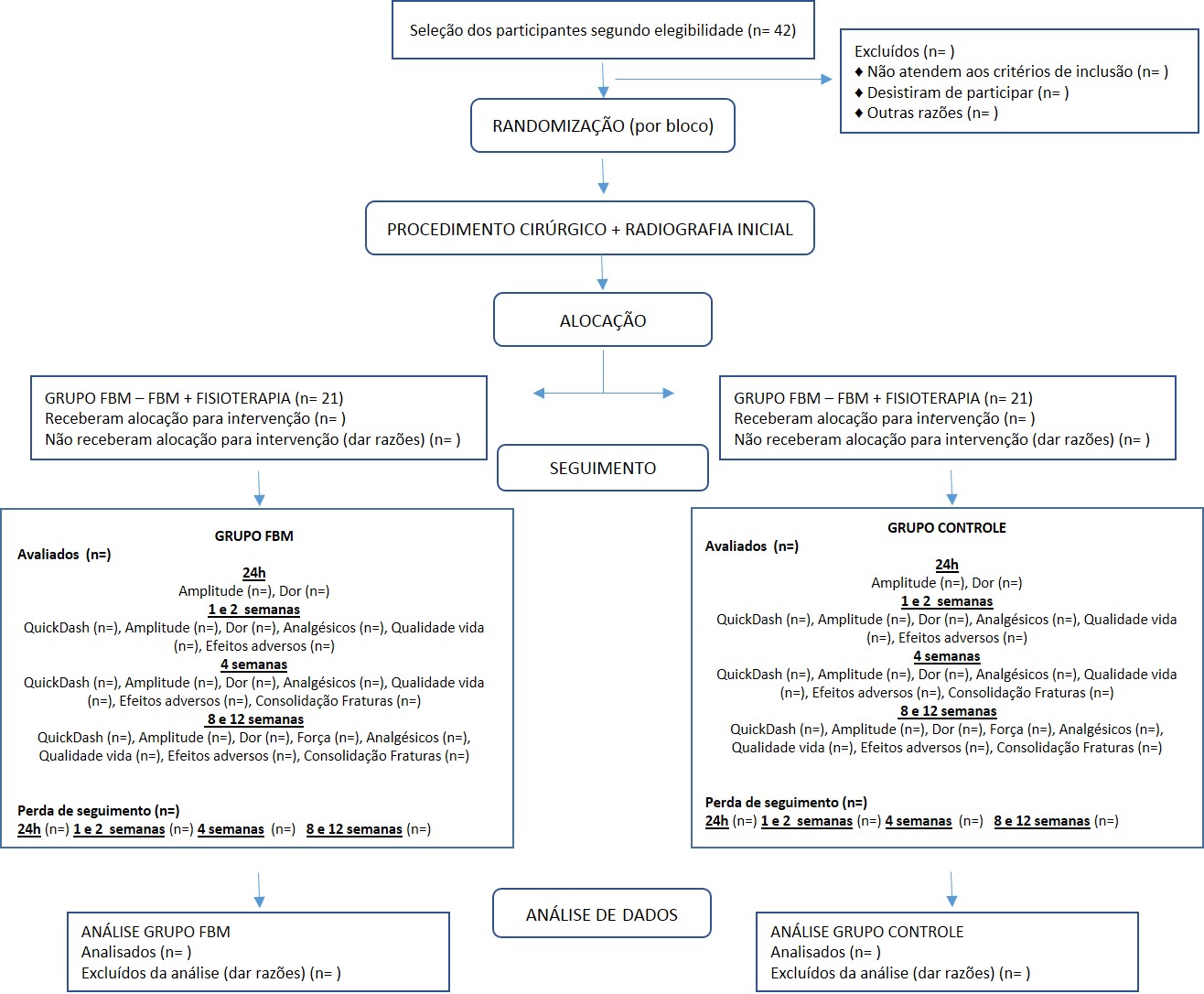


Figura -- Fluxograma

### **4.1. Local de estudo e seleção da amostra**

Os procedimentos cirúrgicos, o acompanhamento ambulatorial e a fisioterapia serão desenvolvidos na Clínica de Ortopedia e Traumatologia do Hospital Dr. Alípio Correa Netto (HMACN) da Secretaria Municipal de Saúde de São Paulo, Brasil no período de abril de 2024 a dezembro de 2025.

### **4.2. Equipe de pesquisa**

A equipe será composta por:

- 1 Pesquisador, que não irá participar de nenhuma avaliação, irá preparar a randomização e os envelopes para garantir o sigilo da alocação.

- 1 Pesquisador (médico residente) que, a partir da inserção dos sujeitos na pesquisa, irá retirar o envelope com a alocação e entregará o equipamento de FBM adequado (ativo ou placebo) assim como fará as recomendações para seu uso em domicílio.

- 4 cirurgiões com experiência em FUP, responsáveis por todos os procedimentos cirúrgicos e pela avaliação de elegibilidade.

- 4 examinadores médicos ortopedistas (cegos quanto ao grupo experimental de cada participante) que estarão encarregados de todas as avaliações desde o pós-operatório até o acompanhamento ambulatorial.

- 1 Pesquisador principal, médico ortopedista, com conhecimento dos grupos para os quais os participantes foram alocados durante a fase de coleta até a conclusão da fase de interpretação dos dados. Responsável pela centralização da coleta de dados, não participando de nenhuma avaliação.

- 1 Fisioterapeuta (cego quanto ao grupo experimental de cada participante) que acompanhará os participantes em todas as sessões.

- 3 Médicos residentes (cegos quanto ao grupo experimental de cada participante) farão contato diariamente com os participantes para controle do uso do dispositivo de FBM, orientações, dúvidas etc.

### **4.3. Calibração e treinamento dos examinadores**

O processo de treinamento e calibração dos 4 pesquisadores que realizarão as avaliações pós-operatórias constará de exercícios conjuntos realizados por 3 vezes em 1 voluntário que não fará parte dos grupos experimentais. Os dados serão discutidos entre estes pesquisadores com o propósito de alcançar-se um nível excelente de concordância. Posteriormente, cada examinador executará individualmente as medidas propostas no estudo, em 10 voluntários adultos que também não farão parte da amostra e os dados obtidos serão submetidos ao teste de Coeficiente de Correlação Intraclasses - ICC (FLEISS, 1986). Sendo a concordância entre os examinadores avaliada como excelente, serão realizadas as medidas nos participantes da amostra.

### **4.4. Caracterização dos participantes**

Serão eleitos para inclusão os sujeitos de ambos os gêneros atendidos no hospital HMACN, portadores fraturas isoladas e fechadas do úmero proximal com desvio e indicação cirúrgica submetidos a RAFI com placa bloqueada de estabilidade angular para úmero proximal (Neer 1070, Carrerra et al 2012, Petros 2019) e de acordo com os critérios de elegibilidade a seguir.

Serão incluídos indivíduos com idade entre 18 e 65 anos, de ambos os gêneros, portadores de FUP classificada como:

- Neer grupo III

- Neer grupo IV

- Neer grupo V

- AO/OTA subgrupos A2 e A3

- AO/OTA grupo B

- AO/OTA grupo C somente em pacientes com menos de 55 anos

Serão excluídos indivíduos que:

- Forem portadores de lesões ou sequelas prévias no ombro e cintura escapular ou déficit motor decorrente de lesões neurológicas centrais ou periféricas;

- Forem portadores de fraturas patológicas;

- Evoluam com infecção ou soltura do implante no pós-operatório;

- Apresentarem fraturas ipsilaterais em outras regiões do membro;

- Apresentarem lesão neuro vascular com déficit sensitivo no local da lesão;

- Apresentarem alterações locais ou sistêmicas que contraindiquem a intervenção cirúrgica ou dificultem o pós-operatório;

- Apresentarem histórico de foto sensibilidade;

- Apresentarem desordens neurológicas e psiquiátricas;

- Apresentarem lesões proliferativas ou infecciosas na pele da região do ombro que receberá a luz LED;

- Utilizaram anti-inflamatórios nos últimos 05 dias prévios ao trauma;

- Gestantes

- Que apresentarem intercorrências cirúrgicas tais como lesões neurológicas ou vasculares bem como extensão do traço de fratura durante o ato cirúrgico pois não estarão no padrão desejado de evolução.

### **4.5. Cálculo do tamanho da amostra**

Para determinar o número de participantes em cada grupo experimental, foi realizado cálculo amostral baseado na variabilidade dos resultados do estudo de Chang et al. (2014), que avaliou os efeitos da FBM com laser na recuperação da função de fraturas de punho e mão por meio do questionário Quick DASH. Utilizando o aplicativo *power/sample size calculator* desenvolvido pela British Columbia University e disponível gratuitamente (https://www.stat.ubc.ca/~rollin/stats/ssize/n2.html), a amostra necessária será de 42 indivíduos, sendo 21 por grupo. Os cálculos foram realizados considerando significância de 0,05, poder de 90% e considerando-se uma perda de 15% para o desfecho citado sendo considerado o maior número obtido.

### **4.6. Composição dos grupos**

Os participantes serão divididos em dois grupos:

**- Grupo FBM:** Os participantes receberão a FBM ativa, por meio de aplicação em seu domicílio, associada ao tratamento terapêutico convencional. A descrição do equipamento, parâmetros de dosimetria e frequência da aplicação da FBM estão relatados no item 4.10.

**- Grupo Controle**: Os participantes receberão a FBM placebo, por meio de aplicação em seu domicílio, associada ao tratamento terapêutico convencional. A descrição do equipamento, placebo está no item 4.10.

### **4.7. Randomização e sigilo na alocação**

A geração da sequência e a preparação dos envelopes serão realizados por um pesquisador não envolvido no estudo.

Para distribuir aleatoriamente os participantes nos dois grupos experimentais, será usado um programa gerador de sequência aleatória (<https://www.randomizer.org/tutorial/>) e selecionada a opção de randomização por blocos de 6 participantes, formando assim 7 blocos. Envelopes opacos serão identificados com cada número e no seu interior será inserida uma folha contendo a informação do grupo experimental correspondente conforme a ordem gerada. Os envelopes serão selados e permanecerão lacrados em ordem numérica em caixa plástica num lugar seguro.

Os participantes serão avaliados pelos 4 cirurgiões e quando preencherem todos os critérios de elegibilidade anteriormente descritos serão incluídos no estudo. Todos serão submetidos ao mesmo protocolo cirúrgico. Um envelope será aberto para cada participante no momento da entrega do dispositivo, 24h após o procedimento cirúrgico. A entrega do dispositivo será realizada pelo pesquisador, médico residente, que será ciente das alocações.

### **4.8.** **Procedimentos iniciais e recrutamento de sujeitos**

Os sujeitos serão recrutados dentre os usuários com trauma em ombro com suspeita de fratura que chegarem ao Pronto Socorro do Hospital Professor Dr. Alípio Correa Netto (HMACN). Todos receberão tratamento de acordo com o protocolo padrão de atendimento do serviço.

Os casos serão avaliados pela equipe de ortopedia de plantão, a qual notificará a equipe de pesquisadores. No pronto socorro, os pesquisadores examinadores solicitarão os exames de imagem padrão: radiografias de ombro nas incidências anteroposterior, perfil escapular, axilar e tomografia quando houver indicação. Também serão solicitados exames pré-operatórios: hemograma completo, coagulograma, tipagem sanguínea, dosagens séricas de sódio, potássio, ureia e creatinina, glicemia, eletrocardiograma e radiografias do tórax nas incidências póstero anterior e perfil. Um ecocardiograma será solicitado nos casos indicados. Avaliações de outras especialidades poderão ser requisitadas quando pertinentes.

A possível inclusão de sujeitos do estudo será definida somente após a avaliação clínica e radiográfica, para certificar que sejam elegíveis. Os usuários serão esclarecidos a respeito dos objetivos e métodos do estudo por explicação verbal e leitura sobre os procedimentos a serem usados e os que concordarem assinarão o Termo de Consentimento Livre e Esclarecido (TCLE), que consta do APENDICE 1.

### **4.9. Prescrição medicamentosa**

Durante o período de internação, será prescrito, para analgesia, dipirona 1g EV de 6/6 horas e cloridrato de tramadol 100 mg EV de 8/8 horas, ambos mantidos nas primeiras 24 horas. Caso o paciente necessite de medicação analgésica de resgate, a primeira opção será antecipar o cloridrato de tramadol de 100 mg de 6/6 horas. Sulfato de morfina será a segunda droga de resgaste na dose de 2mg EV até de 8/8 horas. Os pacientes serão reavaliados quanto à necessidade de ajuste dessa prescrição padrão de analgésicos, passando para “se necessária” a prescrição de opioides, quando possível. Todo o consumo de drogas analgésicas será documentado. Não serão prescritos corticoides ou anti-inflamatórios.

No momento da alta hospitalar o participante receberá prescrição de dipirona 1g via oral de 6/6 h por 05 dias e cloridrato de tramadol 50 mg via oral de 8/8h (se necessário) por no máximo 5 dias. O participante será orientado a utilizar o cloridrato de tramadol somente se dor for persistente com uso da dipirona ou no caso de dor moderada (4 a 7 na escala de dor). Caso algum participante relate uso de anti-inflamatório ou corticoide, será excluído da análise estatística e o caso relatado e discutido.

### **4.10. Procedimento cirúrgico**

Todos os participantes serão submetidos a anestesia geral associada a bloqueio do plexo braquial e procedimento cirúrgico conforme padrão do serviço de ortopedia do HMACN.

As fraturas serão abordadas por incisão delto peitoral em posição de cadeira de praia. Será realizada dissecção cuidadosa evitando exposição excessiva dos fragmentos e preservando a vascularização. Os fragmentos serão reduzidos anatomicamente (Ratajczak et al 2019) por manobras indiretas e estabilizados temporariamente com fios de Kirschner (Biomecânica, Jaú, SP, Brasil). A estabilização definitiva será realizada com placa bloqueada anatômica para úmero proximal (GM Reis, Campinas, SP, Brasil) de 3,5 mm, padrão ouro para estabilização de FUP (Ratajcazak et al 2019, Oldrini et al 2022), posicionada 1 cm abaixo da extremidade superior da tuberosidade maior e 1 cm lateral ao tendão do cabo longo do bíceps, conforme pode ser observado na figura. A redução e o posicionamento da placa e dos parafusos serão avaliados por fluoroscopia durante o ato cirúrgico.


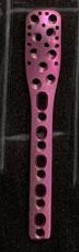


Figura – Placa bloqueada. Fonte: próprio autor

### **4.11. Fisioterapia**

Todos os participantes serão tratados com o protocolo de fisioterapia padronizado sugerido por Ratajczak et al 2019 e pelo grupo AO TRAUMA (<https://www.aofoundation.org/trauma>) e descrito abaixo:

Estágio 1: primeiras 3 semanas com início 24 horas após cirurgia.

- Imobilização com tipoia no membro operado sem sustentação de peso;
- Iniciação de exercícios pendulares;
- Movimentos passivos assistidos;
- Evitar rotação externa nas primeiras 6 semanas.

Estágio 2: terceira a nona semana, desde que não haja anormalidades na consolidação ou desvio secundário dos fragmentos.

- Exercícios ativos assistidos, flexão e abdução da articulação do ombro
- Exercícios ativos cuidadosos com abdução do membro até que dor ocorra, sem carga nas primeiras 6 semanas. Acrescentar carga gradualmente.

Estágio 3: após a 9 semana

- Exercícios isotônicos, excêntricos e concêntricos;
- Em participantes que apresentarem consolidação radiográfica da fratura (avaliada na 12 semana) e contratura articular, serão indicados exercícios passivos de alongamento com profissional experiente.

Para receber o tratamento fisioterápico, os participantes terão que comparecer em consulta com duração 30 minutos, 2 vezes por semana, por 12 semanas.

### **4.12. Aplicação da fotobiomodulação (FBM)**

A FBM será aplicada com dispositivos de LED no formato de ombreira. As aplicações irão ocorrer todos os dias do período experimental a partir de 24h do procedimento cirúrgico. As aplicações ocorrerão no próprio domicílio do participante que será orientado para vestir e manusear o equipamento no momento da alta hospitalar e irá receber também as orientações por escrito (APÊNDICE 9). Cada aplicação terá duração de 10 minutos.

A ombreira da marca Cosmedical (Mauá, SP, Brasil) contém 159 LEDs vermelhos e 159 LEDs infravermelhos intercalados. A área correspondente à placa de fixação, adicionada de margem de segurança de 2 cm em toda extensão, não será irradiada (não serão colocados LEDs) para evitar possível aquecimento da placa por absorção de energia.

Os participantes alocados no grupo controle receberão um dispositivo idêntico ao ativo, porém somente a luz do plug de ativação e o som serão acionados no momento que apertarem o botão, permanecendo os LEDs internos desligados.


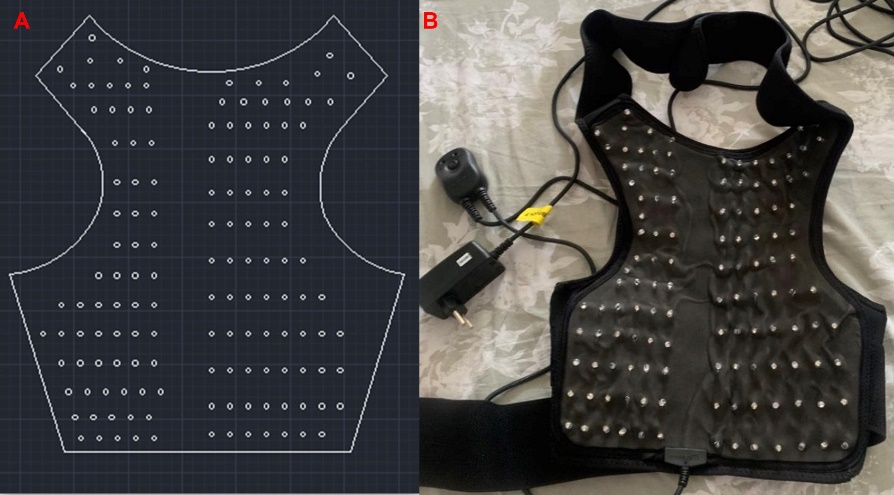


Figura . Projeto evidenciando área em vermelho correspondente à localização da placa bloqueada que não receberá LEDs (A), modelo (B) da ombreira para aplicação da FBM com área isenta de LEDs.

Os relatos da literatura sobre o uso da FBM após fraturas ósseas indicam o uso de fontes vermelhas e infravermelhas com diferentes parâmetros dosimétricos (Tabela 1), sendo que as exposições radiantes variam de 3 a 16 J/cm^2^. O trabalho de Chang et al. (2014), que serviu de base para o cálculo amostral do presente projeto, encontrou efeitos positivos da FBM na recuperação da função de fraturas de punho e mão, por meio do questionário Quick DASH, usando exposição radiante de 9,7 J/cm^2^. Deste modo, neste projeto foram escolhidas exposições radiantes de 10 J/cm^2^ e 12 J/cm^2^.

Tabela 1. Ensaios clínicos randomizados controlados sobre FBM em fraturas ósseas

No presente estudo os parâmetros dosimétricos (Tabela 2) permitirão aliar ondas de maior e menor energia (vermelhos e infravermelhos respectivamente), de maior e menor penetração (infravermelhos e vermelhos respectivamente) com energia por ponto e exposições radiantes dentro dos limites relatados na literatura. Para cálculo dos parâmetros, a potência radiante de cada LED foi mensurada com um medidor de potência com sensor piroelétrico (Coherent, modelo Field Max2, Wilsonville, OR, Estados Unidos).

Tabela 2: parâmetros dosimétricos do dispositivo de FBM

| **Parâmetro** | **FBM** | **FBM** |
| --- | --- | --- |
|  | **vermelho** | **infravermelho** |
| **Comprimento de onda central [nm]** | **660** | **850** |
| **Largura espectral banda (FWHM) [nm]** | **19** | **30** |
| **Modo de operação** | **contínuo** | **contínuo** |
| **Potência radiante por LED [mW]** | **28,5** | **23** |
| **Quantidade de LEDs** | **159** | **159** |
| **Potência radiante total (mW)** | **4531,5** | **3657** |
| **Energia por LED (J)** | **17** | **14** |
| **Polarização** | **aleatória** | **aleatória** |
| **Diâmetro abertura cada LED [mm]** | **3** | **3** |
| **Irradiância na abertura [mW/cm^2^]** | **20,4** | **16,5** |
| **Perfil do feixe** | **multimodo** | **multimodo** |
| **Tempo de exposição (s)** | **600** | **600** |
| **Exposição radiante por LED [J/cm^2^]** | **12** | **10** |
| **Energia radiante por sessão [J]** | **2719** | **2194** |
| **Modo de aplicação** | **em contato direto com a pele** | |
| **Frequência das sessões** | **1 vez ao dia por 12 semanas** | |

### **4.13. Acompanhamento telefônico diário**

Três médicos residentes farão contato telefônico diariamente com os participantes para controle do uso do dispositivo de FBM, orientações, dúvidas e preenchimento da ficha de controle de dor espontânea e noturna e uso de analgésicos. O primeiro contato será realizado por meio de chamada vídeo para repetir as orientações sobre posicionamento e uso do equipamento. Nos primeiros 5 dias, os participantes serão questionados especificamente sobre o uso da dipirona 1g via oral de 6/6 h e do cloridrato de tramadol 50 mg via oral de 8/8h (prescrito para uso em caso de necessidade).

Além do controle de uso e de desfechos, o contato telefônico visa aumentar a aderência dos participantes. Nestes momentos os participantes poderão também fazer qualquer questionamento, solicitar orientações adicionais etc. O participante só receberá o contato da equipe de pesquisa quando estiver em sua residência, ou seja, depois de sua alta hospitalar, depois de receber todas as orientações e de assinar o TCLE presencialmente no hospital. O modelo de formulário a ser preenchido pelos pesquisadores nesta avaliação está no APÊNDICE 2. No APÊNDICE 10 consta o termo de compromisso para entrega do equipamento.

Será usado um único aparelho celular, com número de linha exclusivo para o projeto, que será mantido no hospital. As mensagens de texto ou voz não envolverão dados sensíveis e serão apagadas após finalização do projeto.

### **4.14. Falha da intervenção**

A falha da terapia será caracterizada caso ocorra qualquer uma das seguintes situações:

I.-impossibilidade de aplicação da FBM diariamente

II.- caso participante desenvolva sangramento, sepse, urticária ou qualquer desconforto que justifique a interrupção da terapia com FBM ou FBM simulada.

III.- interrupção do tratamento, por qualquer outro motivo. Nesses casos, os dados coletados até este ponto serão incluídos na análise estatística através de um modelo de regressão com efeitos mistos.

IV.-falta nas avaliações presenciais ou ausência de resposta nos contatos virtuais programados. Nesses casos, os dados coletados até este ponto serão incluídos na análise estatística através de um modelo de regressão com efeitos mistos.

### **4.15. Avaliação dos desfechos**

### **4.15.1. Questionário QuickDASH (Disabilities of the Arm, Shoulder and Hand)**

Neste projeto será usado o componente de disfunções/sintomas do escore QuickDASH validado para o Brasil disponível na internet e que consta no ANEXO 1 e nos APÊNDICES 3 a 8. (https://dash.iwh.on.ca/sites/dash/public/translations/Scoring_QuickDASH_Portuguese_Brazil.pdf).

O cálculo do Escore disfunção e sintomas será realizado conforme as instruções também disponíveis na internet e que estão transcritas a seguir.

(https://dash.iwh.on.ca/sites/dash/public/translations/Scoring_QuickDASH_Portuguese_Brazil.pdf)

Pelo menos 10 dos 11 itens devem ser respondidos. Cada questão respondida terá um valor máximo de 5. Estes valores serão transformados em um escore de 100, subtraindo 1 e multiplicando por 25. Essa transformação é feita para comparar os escores com outras escalas de 0 a 100. Um escore alto indica grande disfunção.

Escore QuickDASH = [(Soma das respostas / n) -1] X 25

n: indica o número de questões respondidas

O QuickDASH será aplicado após 1,2,4,8 e 12 semanas do procedimento cirúrgico.

### **4.15.2. Amplitude de movimento**

A amplitude dos movimentos do ombro, de ambos os lados, será avaliada com o participante em posição ortostática. A posição máxima tolerada na extensão, flexão, abdução, adução, rotação lateral e rotação medial será anotada pelo avaliador (figura). As rotações serão avaliadas em 0° de abdução e flexão (Gracitelli 2015, Tenor Junior et al 2016). Todas as medidas serão realizadas com goniômetro digital (Kaptron 360, Shenzhen, Dongguan China).


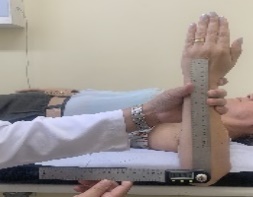

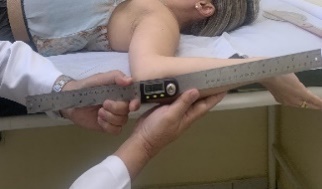

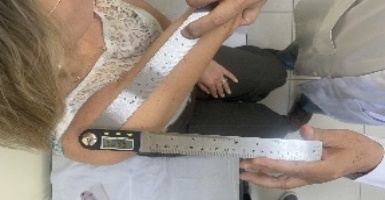

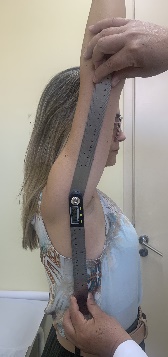

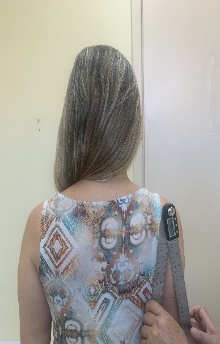

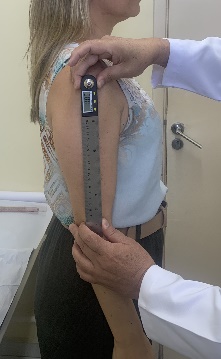

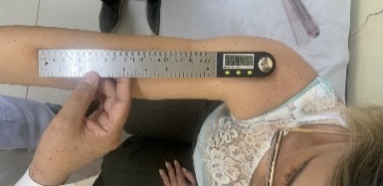

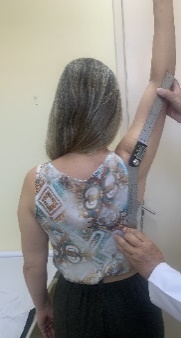

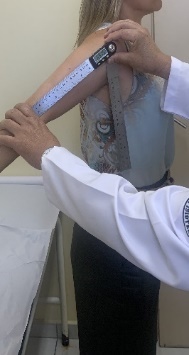

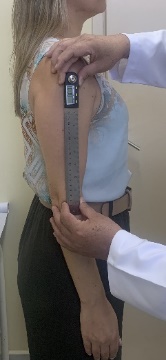


E

A

B

C

D


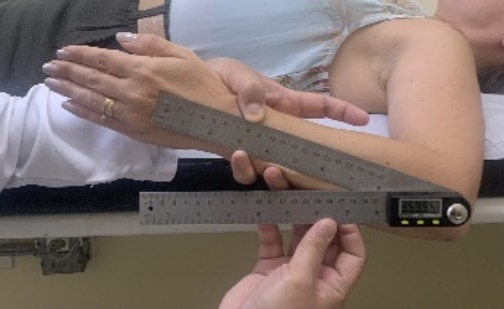

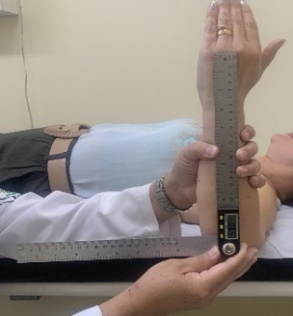


F

Figura. Medida da ADM com goniômetro digital nos movimentos de flexão (A), extensão(B), abdução (C), adução (D), rotação lateral (E) e rotação medial (F) com goniômetro em posição.

A avaliação da amplitude será realizada após 1 dia do ato cirúrgico e posteriormente em 1 e 2 semanas por meio de movimentação passiva. Nas avaliações de 4, 8 e 12 semanas a movimentação será ativa. Os resultados serão transferidos para as respectivas fichas clínicas (APÊNDICES 3 a 8) de cada período experimental.

### **4.15.3. Força muscular**

A força muscular será avaliada, nos períodos de 8 e 12 semanas, por meio da sustentação progressiva de halteres (RLM, Maringá, Paraná, Brasil) com flexão do cotovelo em ambos os braços. A sustentação progressiva será iniciada, no braço não atingido, com 500 gramas e chegará ao peso máximo que o participante conseguir não ultrapassando 05 kg (Barbosa et al 2008). O halter de cada peso será levantado 1 só vez em cada braço. Os resultados serão transferidos para as respectivas fichas clínicas (APÊNDICES 7 a 8) de cada período experimental.

### **4.15.4 Avaliação da dor**

A intensidade da dor espontânea e em função do membro lesionado serão avaliadas por meio da aplicação da escala visual analógica de dor (Jensen et al 1986). A escala escolhida, representada na figura, contém uma linha numerada de 10 cm, que traz desenhos de expressões faciais, escala de cores e indicação de intensidades leve, moderada e intensa para facilitar a compreensão (Thong et al 2018).


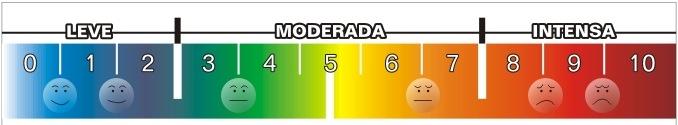


Figura . Escala visual analógica de dor

A dor à pressão no local da fratura será avaliada por algometro digital (MED DOR, Governador Valadares, MG, Brasil).


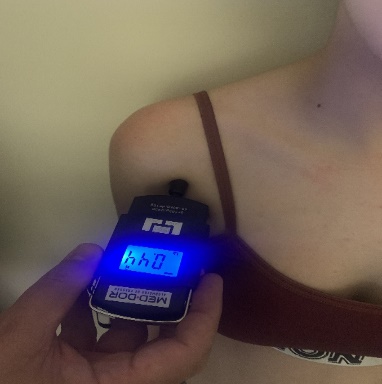


Figura. Algometro digital em posição para mensurar dor à pressão

Os resultados referentes à dor espontânea, em função (flexão e abdução) com uso do algometro serão coletados após 1 dia do ato cirúrgico e posteriormente em 1, 2, 4, 8 e 12 semanas e transferidos para as respectivas fichas clínicas (APÊNDICES 3 a 8) de cada período experimental.

A ocorrência de dor noturna será questionada no momento do controle diário do uso da FBM e irá constar da ficha de controle diário do participante (APÊNDICE 2).

### **4.15.5. Consumo de analgésicos**

O tipo e dosagem de analgésicos consumidos serão questionados no momento do controle diário do uso da FBM e irão constar da ficha de controle diário do participante (APÊNDICE 2).

### **4.15.6. Qualidade de vida**

A qualidade de vida será avaliada por meio do uso do instrumento SF-6 de 2002 (ANEXO 3) na versão adaptada para uso no Brasil (Campolina et al 2011) nos períodos de 1, 2, 4, 8 e 12 semanas e os resultados anotados nas respectivas fichas clínicas (APÊNDICES 3 a 8) de cada período experimental. Na versão brasileira mais recente do SF-6 (2002), assim como na original, foram incluídos 6 itens: capacidade funcional (itens 1, 2 e 10); limitação global (item 3 de aspectos físicos e item 2 de aspectos emocionais); aspectos sociais (item 2); dor (todos os itens); saúde mental (item 1); e vitalidade (item 2) (Campolina et al 2011).

**4.15.7. Ocorrência de efeitos adversos**

A ocorrência de efeitos adversos será questionada no momento do controle diário do uso da FBM e irá constar da ficha de controle diário do participante (APÊNDICE 2).

### **4.15.8. Consolidação das fraturas**

Radiografias de ombro nas incidências anteroposterior, perfil escapular e axilar serão tomadas após 4, 8 e 12 semanas para avaliação da consolidação óssea definida pela presença de calo ósseo em 3 das 4 corticais ósseas unindo os fragmentos principais da fratura sendo confrontadas com a radiografia tomada no pós-cirúrgico imediato (Gracitelli 2015).

### **4.15.9. Análise de custos**

Os valores serão levantados a partir da perspectiva do Sistema Único de Saúde (SUS) como comprador do serviço. Para tanto serão considerados os custos diretos dos procedimentos realizados no período da pesquisa e computados os valores dos materiais utilizados, honorários profissionais, custos hospitalares e custos com a perda de produtividade durante período de afastamento do trabalho. As fontes de referência dos custos em moeda brasileira (Real) serão os bancos de dados ComprasNet, SIGTAP e Banco de Preços do Ministério da Saúde.

A análise final do custo-efetividade e custo incremental seguirá as Diretrizes Metodológicas: Estudos de Avaliação Econômica de Tecnologias em Saúde e Política Nacional de Gestão de Tecnologias em Saúde (BRASIL, 2010).

Tabela 3: Custos do tratamento

| **Custos** | **Valor estimado (R$)** |
| --- | --- |
|  |  |
| Diária de internação rede pública enfermaria |  |
| Atendimento ortopédico com imobilização provisória |  |
| Taxa sala de pronto socorro |  |
| Taxas de sala de gesso  Solução antisséptica  Fios de sutura  Compressa cirúrgica |  |
| Arco cirúrgico/ intensificador de imagem (uso) |  |
| Perfurador pneumático p/ cirurgia (uso) |  |
| Aspirador (uso) |  |
| Oxímetro (uso) |  |
| Monitor multifunção (hora) |  |
| Carro de anestesia (uso) |  |
| Dipirona Monoidratada ampola 2 ml |  |
| Cloridrato de tramadol 100 mg ampola  Enoxaparina 40 mg ampola  Omeprazol ampola 40 mg  Ondansetrona 8 mg ampola |  |
| Cefazolina sódica 1g ampola  Sulfato de morfina 2 ml ampola |  |
| Exames de imagem (TC e RX) |  |
| Gaze estéril 13 fios |  |
| Cremer atadura de crepe 10cm x 1,8mt |  |
| Troca de curativo hospitalar |  |
| Diária de trabalhador afastado (custo indireto) |  |
| Auxílio-doença (estimado, custo indireto) |  |
| Sessão de Fisioterapia |  |
| **Dispositivo de FBM** |  |
| **TOTAL** |  |

# **ANÁLISE DOS RESULTADOS**

Serão realizadas análises descritivas iniciais considerando todas as variáveis medidas no estudo, tanto quantitativas (média e desvio padrão) quanto qualitativas (frequências e porcentagens). Posteriormente serão realizadas as análises de normalidade para determinar os testes estatísticos apropriados para cada conjunto de dados e aplicados os testes estatísticos apropriados para cada análise específica. Serão realizadas também análises por subgrupos (presença de osteoporose, osteopenia, traumas de alta e baixa intensidade, tabagismo, outras comorbidades). Em todos os testes, será adotado o nível de significância de 5% de probabilidade ou o p-valor correspondente. Todas as análises serão realizadas utilizando o programa estatístico SAS for Windows, versão 9.1.

# **BIBLIOGRAFIA**

Abhishek gujar, Mitushi Deshmukh. An overview on low laser therapy in distal radius fracture. Journal of Pharmaceutical Negative Results [Internet]. 2022 Oct. 17 [cited 2022 Nov. 10]:2930-2

Alispahic N, Brorson S, Bahrs C, Joeris A, Steinitz A, Audigé L. Complications after surgical management of proximal humeral fractures: a systematic review of event terms and definitions. BMC Musculoskelet Disord. 2020 May 26;21(1):327. doi: 10.1186/s12891-020-03353-8. PMID: 32456631; PMCID: PMC7251821.

Audigé L, Brorson S, Durchholz H, Lambert S, Moro F, Joeris A. Core set of unfavorable events of proximal humerus fracture treatment defined by an international Delphi consensus process. BMC Musculoskelet Disord. 2021 Nov 30;22(1):1002. doi: 10.1186/s12891-021-04887-1. PMID: 34847888; PMCID: PMC8630858.

Barbosa RI, Marcolino AM, Fonseca MCR, Mazzer N, Zatiti SC. Retrospective functional assessment of patients with humerus proximal fractures fixed internally with a fixed-angle plate of the proximal humerus. Acta Ortop Bras 16(2): 89-92, 2008

Barreto RPG, Barbosa MLL, Balbinotti MAA, Mothes FC, Rosa LHT, Silva MF. The Brazilian version of the Constant-Murley Score (CMS-BR): convergent and construct validity, internal consistency, and unidimensionality. Revista Brasileira de Ortopedia [online]. 2016, v. 51, n. 05 [Accessed 15 December 2022], pp. 515-520. Available from: <https://doi.org/10.1016/j.rboe.2016.08.017>.<https://doi.org/10.1016/j.rboe.2016.08.017>.

Beaton DE, Wright JG, Katz JN; Upper Extremity Collaborative Group. Development of the QuickDASH: comparison of three item-reduction approaches. J Bone Joint Surg Am. 2005 May;87(5):1038-46. doi: 10.2106/JBJS.D.02060. PMID: 15866967.

Bougher H, Nagendiram A, Banks J, Hall LM, Heal C. Imaging to improve agreement for proximal humeral fracture classification in adult patient: A systematic review of quantitative studies. J Clin Orthop Trauma. 2020 Feb;11(Suppl 1):S16-S24. doi: 10.1016/j.jcot.2019.06.019. Epub 2019 Jun 26. PMID: 31992911; PMCID: PMC6977161.

Brasil. Ministério da Saúde. Secretaria de Ciência, Tecnologia e Insumos Estratégicos. Departamento de Ciência e Tecnologia. Política Nacional de Gestão de Tecnologias em Saúde / Ministério da Saúde, Secretaria de Ciência, Tecnologia e Insumos Estratégicos, Departamento de Ciência e Tecnologia. – Brasília: Ministério da Saúde, 2010. 48 p. – (Série B. Textos Básicos em Saúde).

Brorson S, Elliott J, Thillemann T, Aluko P, Handoll H. Interventions for proximal humeral fractures: key messages from a Cochrane review. Acta Orthop. 2022 Jul 4;93:610-612. doi: 10.2340/17453674.2022.3495. PMID: 35819454; PMCID: PMC9275419.

Campolina AG, Bortoluzzo AB, Ferraz MB, Ciconelli RM. O questionário SF-6D Brasil: modelos de construção e aplicações em economia da saúde. Rev Assoc Med Bras [Internet]. 2010;56(Rev. Assoc. Med. Bras., 2010 56(4)). Available from: https://doi.org/10.1590/S0104-42302010000400012

Campolina AG, Bortoluzzo AB, Ferraz MB, Ciconelli RM. Validação da versão brasileira do questionário genérico de qualidade de vida short-form 6 dimensions (SF-6D Brasil). Ciênc saúde coletiva [Internet]. 2011Jul;16(Ciênc. saúde coletiva, 2011 16(7)). Available from: https://doi.org/10.1590/S1413-81232011000800010

Carrerra, Eduardo da Frota et al. Reproducibility of three classifications of proximal humeral fractures. Einstein (São Paulo) [online]. 2012, v. 10, n. 4 [Accessed 3 November 2022], pp. 473-479. Available from: <https://doi.org/10.1590/S1679-45082012000400014>. Epub 22 Jan 2013. ISSN 2317-6385. <https://doi.org/10.1590/S1679-45082012000400014>.

Chan AW, Tetzlaff JM, Altman DG, Laupacis A, Gøtzsche PC, Krleža-Jerić K, Hróbjartsson A, Mann H, Dickersin K, Berlin JA, Doré CJ, Parulekar WR, Summerskill WS, Groves T, Schulz KF, Sox HC, Rockhold FW, Rennie D, Moher D. SPIRIT 2013 statement: defining standard protocol items for clinical trials. Ann Intern Med. 2013 Feb 5;158(3):200-7. doi: 10.7326/0003-4819-158-3-201302050-00583.

Chang WD, Wu JH, Wang HJ, Jiang JA (2014) Therapeutic outcomes of low-level laser therapy for closed bone fracture in the human wrist and hand. Photomed Laser Surg 32(4):212–218.

Chauhan A, Sarin P. Low Level Laser Therapy in Treatment of Stress Fractures Tibia: A Prospective Randomized Trial. Med J Armed Forces India. 2006 Jan;62(1):27-9. doi: 10.1016/S0377-1237(06)80148-6. Epub 2011 Jul 21. PMID: 27407838; PMCID: PMC4923284.

Cheng W, Yao M, Sun K, Li W. Progress in Photobiomodulation for Bone Fractures: A Narrative Review. Photobiomodul Photomed Laser Surg. 2020 May;38(5):260-271. doi: 10.1089/photob.2019.4732. PMID: 32427551.

Constant CR, Murley AH. A Clinical method of functional assessment of the shoulder. Clin Orthop Relat Res. 1987;(214):160-4.

Court-Brown CM, Caesar B. Epidemiology of adult fractures: A review. Injury. 2006 Aug;37(8):691-7. doi: 10.1016/j.injury.2006.04.130. Epub 2006 Jun 30. PMID: 16814787.

da Silva NC, Chaves TC, Dos Santos JB, Sugano RMM, Barbosa RI, Marcolino AM, Mazzer N, Fonseca MCR. Reliability, validity and responsiveness of Brazilian version of QuickDASH. Musculoskelet Sci Pract. 2020 Aug;48:102163. doi: 10.1016/j.msksp.2020.102163. Epub 2020 Apr 5. PMID: 32560867.

Davey MS, Hurley ET, Anil U, Condren S, Kearney J, O'Tuile C, Gaafar M, Mullett H, Pauzenberger L. Management options for proximal humerus fractures - A systematic review & network meta-analysis of randomized control trials. Injury. 2022 Feb;53(2):244-249. doi: 10.1016/j.injury.2021.12.022. Epub 2021 Dec 15. PMID: 34974908.

Deana AM, de Souza AM, Teixeira VP, Mesquita-Ferrari RA, Bussadori SK, Fernandes KPS. The impact of photobiomodulation on osteoblast-like cell: a review. Lasers Med Sci. 2018 Jul;33(5):1147-1158. doi: 10.1007/s10103-018-2486-9. Epub 2018 Mar 23. PMID: 29572767.

de Freitas LF, Hamblin MR. Proposed Mechanisms of Photobiomodulation or Low-Level Light Therapy. IEEE J Sel Top Quantum Electron. 2016 May-Jun;22(3):7000417. doi: 10.1109/JSTQE.2016.2561201. PMID: 28070154; PMCID: PMC5215870.

Escudero JSB, Perez MGB, de Oliveira Rosso MP, Buchaim DV, Pomini KT, Campos LMG, Audi M, Buchaim RL. Photobiomodulation therapy (PBMT) in bone repair: A systematic review. Injury. 2019 Nov;50(11):1853-1867. doi: 10.1016/j.injury.2019.09.031. Epub 2019 Sep 21. PMID: 31585673.

EuroQol. EQ-5D value sets: inventory, comparative review and user guide, 2010. EQ-5D value sets The EuroQol Group’s Task Force On Value Sets, 2010.

Garrat AM, Schmidt L, Mackintosh A, Fitzpatrick R. Quality of life measurement: bibliographic study of patient assessed health outcome measures. BMJ 2002; 324(7351):1417-1421

Gracitelli, Mauro Emilio Conforto. Estudo randomizado da osteossíntese das fraturas da extremidade proximal do úmero com placa ou haste intramedular [tese]. São Paulo: , Faculdade de Medicina; 2015 [citado 2023-02-18]. doi:10.11606/T.5.2016.tde-24022016-091653.

Gummesson C, Ward MM, Atroshi I. The shortened disabilities of the arm, shoulder and hand questionnaire (QuickDASH): validity and reliability based on responses within the full-length DASH. BMC Musculoskelet Disord. 2006 May 18;7:44. doi: 10.1186/1471-2474-7-44. PMID: 16709254; PMCID: PMC1513569.

Hamblin MR. Mechanisms and applications of the anti-inflammatory effects of photobiomodulation. AIMS Biophys. 2017;4(3):337-361. doi: 10.3934/biophy.2017.3.337. Epub 2017 May 19. PMID: 28748217; PMCID: PMC5523874.

Handoll HH, Elliott J, Thillemann TM, Aluko P, Brorson S. Interventions for treating proximal humeral fractures in adults. Cochrane Database Syst Rev. 2022 Jun 21;6(6):CD000434. doi: 10.1002/14651858.CD000434.pub5. PMID: 35727196; PMCID: PMC9211385.

Iglesias-Rodríguez, S., Domínguez-Prado, D.M., García-Reza, A. *et al.* Epidemiology of proximal humerus fractures. *J Orthop Surg Res* **16**, 402 (2021).

Jana Neto FC, Martimbianco ALC, Mesquita-Ferrari RA, Bussadori SK, Alves GP, Almeida PVD, Delgado FG, Fonseca LR, Gama MZG, Jorge MD, Hamblin MR, Fernandes KPS. Effects of multiwavelength photobiomodulation for the treatment of traumatic soft tissue injuries associated with bone fractures: A double-blind, randomized controlled clinical trial. J Biophotonics. 2023 Jan 14:e202200299. doi: 10.1002/jbio.202200299. Epub ahead of print. PMID: 36640122.

Jensen MP, Karoly P, Braver S. The measurement of clinical pain intensity: a comparison of six methods. Pain. 1986;27(1):117-26.

Kennedy CA, Beaton DE. A user's survey of the clinical application and content validity of the DASH (Disabilities of the Arm, Shoulder and Hand) outcome measure. J Hand Ther. 2017 Jan-Mar;30(1):30-40.e2. doi: 10.1016/j.jht.2016.06.008. Epub 2016 Jul 26. PMID: 27469538.

Monticone M, Portoghese I, Cazzaniga D, Liquori V, Marongiu G, Capone A, Campagna M, Zatti G. Task-oriented exercises improve disability of working patients with surgically-treated proximal humeral fractures. A randomized controlled trial with one-year follow-up. BMC Musculoskelet Disord. 2021 Mar 20;22(1):293. doi: 10.1186/s12891-021-04140-9. PMID: 33743670; PMCID: PMC7981858.

Neer II CS. Displaced proximal humerus fractures. Part I. Classification and evaluation. J Bone Joint Surg 1970;52A:1077–1089.

Nesioonpour S, Mokmeli S, Vojdani S, Mohtadi A, Akhondzadeh R, Behaeen K, Moosavi S, Hojjati S. The effect of low-level laser on postoperative pain after tibial fracture surgery: a double-blind controlled randomized clinical trial. Anesth Pain Med. 2014 Jun 21;4(3):e17350. doi: 10.5812/aapm.17350. PMID: 25237637; PMCID: PMC4165037.

Neto FCJ, Martimbianco ALC, de Andrade RP, Bussadori SK, Mesquita-Ferrari RA, Fernandes KPS. Effects of photobiomodulation in the treatment of fractures: a systematic review and meta-analysis of randomized clinical trials. Lasers Med Sci. 2020 Apr;35(3):513-522. doi: 10.1007/s10103-019-02779-4. Epub 2019 Apr 13. PMID: 30982176.

Nowak LL, Davis AM, Mamdani M, Beaton D, Schemitsch EH. A concept analysis and overview of outcome measures used for evaluating patients with proximal humerus fractures. Disabil Rehabil. 2021 May;43(10):1450-1462. doi: 10.1080/09638288.2019.1649728. Epub 2019 Sep 3. PMID: 31479302.

Nussbaum EL, Downes L. Reliability of clinical pressurepain algometric measurements obtained on consecutive days. Phys Ther 1998;78:160–169.

Oldrini LM, Feltri P, Albanese J, Marbach F, Filardo G, Candrian C. PHILOS Synthesis for Proximal Humerus Fractures Has High Complications and Reintervention Rates: A Systematic Review and Meta-Analysis. Life (Basel). 2022 Feb 19;12(2):311. doi: 10.3390/life12020311. PMID: 35207598; PMCID: PMC8880552.

Orfale AG, Araújo PM, Ferraz MB, Natour J. Translation into Brazilian Portuguese, cultural adaptation and evaluation of the reliability of the Disabilities of the Arm, Shoulder and Hand Questionnaire. Braz J Med Biol Res. 2005 Feb;38(2):293-302. doi: 10.1590/s0100-879x2005000200018. Epub 2005 Feb 15. PMID: 15785841.

Peluso R, Hesson J, Aikens J, Bullock M. An Update on Physical Therapy Adjuncts in Orthopedics. Arthroplast Today. 2022 Mar 18;14:163-169. doi: 10.1016/j.artd.2022.02.013. PMID: 35330664; PMCID: PMC8938198.

Petros RSB, Ribeiro FR, Tenor AC, Brasil R, Filardi CS, Molin DCD. PROXIMAL HUMERUS FRACTURE WITH LOCKING PLATE: FUNCTIONAL AND RADIOGRAPHIC RESULTS. Acta ortop bras [Internet]. 2019May;27(Acta ortop. bras., 2019 27(3)). Available from: https://doi.org/10.1590/1413-785220192703142049

Rabi S, Evaniew N, Sprague SA, Bhandari M, Slobogean GP. Operative vs non-operative management of displaced proximal humeral fractures in the elderly: A systematic review and meta-analysis of randomized controlled trials. World J Orthop. 2015 Nov 18;6(10):838-46. doi: 10.5312/wjo.v6.i10.838. PMID: 26601066; PMCID: PMC4644872.

Ratajczak K, Szczęsny G, Małdyk P. Comminuted fractures of the proximal humerus - principles of the diagnosis, treatment and rehabilitation. Ortop Traumatol Rehabil. 2019 Apr 30;21(2):77-93. doi: 10.5604/01.3001.0013.1544. PMID: 31180034.

Relvas Silva M, Linhares D, Leite MJ, Nunes B, Torres J, Neves N, Ribeiro Silva M. Proximal humerus fractures: epidemiology and trends in surgical management of hospital-admitted patients in Portugal. JSES Int. 2022 Jan 24;6(3):380-384. doi: 10.1016/j.jseint.2021.12.003. PMID: 35572441; PMCID: PMC9091738.

Richard GJ, Denard PJ, Kaar SG, Bohsali KI, Horneff JG, Carpenter S, Fedorka CJ, Mamelson K, Garrigues GE, Namdari S, Abboud JA, Paxton ES, Kovacevic D, Hebert-Davies J, Ponce BA, King JJ. Outcome measures reported for the management of proximal humeral fractures: a systematic review. J Shoulder Elbow Surg. 2020 Oct;29(10):2175-2184. doi: 10.1016/j.jse.2020.04.006. Epub 2020 Jun 9. PMID: 32951643.

Roux A, Decroocq L, El Batti S, Bonnevialle N, Moineau G, Trojani C, et al. Epidemiology of proximal humerus fractures managed in a trauma center. Orthop Traumatol Surg Res. 2012;98(6):715–9. <https://doi.org/10.1016/j.otsr.2012.05.013>.

Saebo H, Naterstad IF, Bjordal JM, Stausholm MB, Joensen J. Treatment of Distal Radius Fracture During Immobilization with an Orthopedic Cast: A Double- Blinded Randomized Controlled Trial of Photobiomodulation Therapy. Photobiomodul Photomed Laser Surg. 2021 Apr;39(4):280-288. doi: 10.1089/photob.2020.4964. Epub 2021 Mar 19. PMID: 33751924.

Sæbø H, Naterstad IF, Joensen J, Stausholm MB, Bjordal JM. Pain and Disability of Conservatively Treated Distal Radius Fracture: A Triple-Blinded Randomized Placebo-Controlled Trial of Photobiomodulation Therapy. Photobiomodul Photomed Laser Surg. 2022 Jan;40(1):33-41. doi: 10.1089/photob.2021.0125. PMID:

35030040.

Saebo H, Naterstad IF, Stausholm MB, Bjordal JM, Joensen J. Reliability of pain pressure threshold algometry in persons with conservatively managed wrist fractures. Physiother Res Int 2020;25:e1797

Schnackers MLAP, van Horn YY, Meys GHH, Brink PRG, Smeets RJEM, Seelen HAM. Evidence-based rehabilitation therapy following surgery for (peri-)articular fractures: A systematic review. J Rehabil Med. 2019 Oct 4;51(9):638-645. doi: 10.2340/16501977-2599. PMID: 31495902.

Sharma SK, Sardana S, Hamblin MR. Role of opsins and light or heat activated transient receptor potential ion channels in the mechanisms of photobiomodulation and infrared therapy. Journal of Photochemistry and Photobiology (2023), doi: <https://doi.org/10.1016/j.jpap.2023.100160>

Slobogean GP, Noonan VK, O’Brien PJ. The reliability and validity of the Disabilities of Arm, Shoulder, and Hand, EuroQol-5D, Health Utilities Index, and Short Form-6D outcome instruments in patientswith proximal humeral fractures. J Shoulder Elbow Surg 2010;19:342-8. <https://doi.org/10.1016/j.jse.2009.10.021>

Tenor Junior AC, Granja Cavalcanti AM, Albuquerque BM, Ribeiro FR, da Costa MP, Filho RB. Treatment of proximal humeral fractures using anatomical locking plate: correlation of functional and radiographic results. Rev Bras Ortop. 2016 Apr 19;51(3):261-7. doi: 10.1016/j.rboe.2015.08.018. PMID: 27284546; PMCID: PMC4887510.

Thong ISK, Jensen MP, Miro J, Tan G. The validity of pain intensity measures: what do the NRS, VAS, VRS, and FPS-R measure? Scand J Pain. 2018;18(1):99-107.

van de Water AT, Shields N, Davidson M, Evans M, Taylor NF. Reliability and validity of shoulder function outcome measures in people with a proximal humeral fracture. Disabil Rehabil 2014;36: 1072-9. <https://doi.org/10.3109/09638288.2013.829529>

Ware JE, Sherbourne CD. The MOS 36-item short health survey (SF-36). I. Conceptual framework and item selection. Med Care 1992; 30(6):473-483.
