# Supplementary material for: The role of photobiomodulation in the functional recovery of proximal humerus fractures: a randomized controlled clinical protocol
Source: PLoS One. 2025 Apr 29;20(4):e0321746. doi: 10.1371/journal.pone.0321746 (PMC12040229; doi:10.1371/journal.pone.0321746)
Supplement: S6 Appendix — (PDF) [file pone.0321746.s006.pdf]

ClinicalTrials.gov PRS
Protocol Registration and Results System

ClinicalTrials.gov Protocol Registration and Results System (PRS) Receipt
Release Date: November 16, 2024

ClinicalTrials.gov ID: NCT06113614

Study Identification

Unique Protocol ID: 6.075.552

Brief Title: Effects of Photobiomodulation on Functional Recovery of Proximal Humerus Fractures

Official Title: Effects of Photobiomodulation on Functional Recovery of Proximal Humerus Fractures: a Double-blind Randomized Controlled Clinical Study

Secondary IDs:

Study Status

Record Verification: July 2024

Overall Status: Recruiting

Study Start: April 30, 2024 [Actual]

Primary Completion: December 30, 2025 [Anticipated]

Study Completion: April 30, 2026 [Anticipated]

Sponsor/Collaborators

Sponsor: University of Nove de Julho

Responsible Party: Principal Investigator
Investigator: Kristianne Porta Santos Fernandes [KPSantosFernandes]
Official Title: Principal Invertigator
Affiliation: University of Nove de Julho

Collaborators:

Oversight

U.S. FDA-regulated Drug: No

U.S. FDA-regulated Device: No

U.S. FDA IND/IDE: No

Human Subjects Review: Board Status: Approved
Approval Number: 6.075.552
Board Name: Nove de Julho University Ethics Committee
Board Affiliation: Nove de Julho University
Phone: +55 11 33859197
Email: comitedeetica@uninove.br
Address:

Rua Vergueiro 235/249, Liberdade São Paulo

Data Monitoring: No

FDA Regulated Intervention: No

Study Description

Brief Summary: Among the various complications of the postoperative evolution of proximal humerus fractures (PHF) are pain and joint stiffness, generating significant functional limitation in the affected limb. This randomized controlled double-blind clinical study aims to evaluate the effects of photobiomodulation (PBM) on the functional recovery of participants with PHF surgically treated with special locking plates. The primary outcome, to be assessed at 1, 2, 4, 8, and 12 weeks, will be shoulder function recovery using the Quick-DASH functional scale. Secondary outcomes will include shoulder range of motion assessments with a digital goniometer, quality of life measured using the SF-6 questionnaire, and the occurrence of adverse effects during all experimental periods. Pain will be evaluated at 1, 2, 4, 8, and 12 weeks; fracture consolidation will be assessed through radiographic examinations at 4, 8, and 12 weeks; and muscular strength will be evaluated through progressive weight-bearing exercises at 8 and 12 weeks. Data will be presented as means ± SD, with significance set at a p-value of 0.05.

Detailed Description: Among the complications of postoperative recovery from proximal humerus fractures (PHF) are pain and joint stiffness, which can significantly limit limb function. Physical therapy is standard for both surgical and non-surgical treatments. Research suggests that photobiomodulation (PBM) positively affects fracture repair, regeneration, analgesia, and functional improvement, but more standardization and evidence are needed. This double-blind, randomized controlled study aims to assess PBM's impact on functional recovery in patients with surgically treated PHF using specialized locking plates. Forty-two participants will be randomly assigned (1:1) to either the Control group (standard physiotherapy with simulated PBM) or the FBM group (standard physiotherapy with active PBM). The PBM will be administered at home daily for 10 minutes using a device with 318 LEDs: 159 at 660 nm (28.5 mW; 12 J/cm²; 17 J per LED) and 159 at 850 nm (23 mW; 10 J/cm²; 14 J per LED). PBM and physiotherapy sessions (30 minutes, twice a week) will continue for 12 weeks. Participants and examiners will be blinded to group allocation. The primary outcome is shoulder function recovery, measured by the Quick-DASH scale, that will be evaluated 1, 2, 4, 8, and 12 weeks after surgery . Secondary outcomes include shoulder range of motion (digital goniometer), quality of life (SF-6 questionnaire), and adverse effects. The intensity of spontaneous pain and pain during movement of the injured limb will be assessed using the visual analog scale for pain. Pressure pain at the fracture site will be evaluated with a digital algometer (MED DOR, Governador Valadares, MG, Brazil) at 1, 2, 4, 8, and 12 weeks. Fracture consolidation via radiographs at 4, 8, and 12 weeks, and muscle strength through progressive weight bearing at 8 and 12 weeks. Data will be stored, organized, and analyzed using appropriate statistical tests, with a significance level set at 5%.

Keywords: Photobiomodulation, Humerus Fracture, Quick-DASH, Quality of life, Phototherapy

Conditions

Conditions: Bone Fracture
Humerus Fracture
Surgery

Keywords: photobiomodulation

Study Design

Study Type: Interventional

Primary Purpose: Treatment

Study Phase: N/A

Interventional Study Model: Parallel Assignment
Forty-two participants will be randomly assigned (1:1) into two groups: the Control group (standard physical therapy combined with simulated PBM) and the PBM group (standard physical therapy combined with active PBM). Participants will apply PBM at home daily for 10 minutes using a device containing 318 light-emitting diodes (LEDs), with 159 LEDs at 660 nm (28.5 mW; 12 J/cm2; 17 J per LED) and 159 LEDs at 850 nm (23 mW; 10 J/cm2; 14 J per LED). PBM and physical therapy sessions (30 minutes, twice a week) will be conducted over 12 weeks.

Number of Arms: 2

Masking: Double (Participant, Outcomes Assessor)
1 Researcher, who will prepare the randomization and envelopes to ensure allocation concealment.

1 Researcher (resident physician) who will include subjects, open the envelope with the allocation, deliver the appropriate PBM device, and provide instructions.

4 Surgeons all surgical procedures and eligibility assessments. 4 Orthopedic physician evaluators (blinded to the experimental group of each participant), responsible for all evaluations from the postoperative period through outpatient follow-up.

1. The Principal Investigator will be aware of the groups; he will centralize data collection and will not participate in any evaluations.

1 Physical Therapist (blinded to the experimental group of each participant)
3 resident Physicians (blinded to each participant's experimental group) will contact participants daily

Allocation: Randomized

Enrollment: 42 [Anticipated]

Arms and Interventions

Arms	Assigned Interventions
Experimental: Photobiomodulation Group<br>The shoulder brace (Cosmedical, Mauá, SP, Brazil) contains 159 red LEDs and 159 infrared LEDs interspersed. The PBM group (standard physical therapy combined with active PBM). Participants will apply PBM at home daily for 10 minutes using a device containing 318 light-emitting diodes (LEDs), with 159 LEDs at 660 nm (28.5 mW; 12 J/cm2; 17 J per LED) and 159 LEDs at 850 nm (23 mW; 10 J/cm2; 14 J per LED). PBM and physical therapy sessions (30 minutes, twice a week) will be conducted over 12 weeks.	Procedure/Surgery: Physiotherapy<br>All participants will be treated with the standardized physiotherapy protocol suggested by Ratajczak et al. 2019. To receive physiotherapy treatment, participants will need to attend a 30-minute session, twice a week, for 12 weeks.<br>Device: Photobiomodulation<br>The PBM will be applied with LED devices in the form of a shoulder brace. The applications will occur every day starting 24 hours after the surgical procedure. The shoulder brace from the brand Cosmedical (Mauá, SP, Brazil) contains 70 red LEDs and 70 infrared LEDs. The area corresponding to the fixation plate will

Arms	Assigned Interventions
	not be irradiated (no LEDs will be placed). PBM will be applied every day for 10 minutes using a device containing 318 light-emitting diodes (LEDs), including 159 LEDs at 660 nm (28.5 mW; 12 J/cm2 to 17 J) and 159 LEDs at 850 nm (23 mW; 10 J/cm2 to 14 J).
Sham Comparator: Control Group<br>In the control group (standard physical therapy combined with simulated PBM), participants will receive an identical device to the active one, but only the light from the activation plug and the sound will be triggered when they press the button, while the internal LEDs will remain off.	Procedure/Surgery: Physiotherapy<br>All participants will be treated with the standardized physiotherapy protocol suggested by Ratajczak et al. 2019. To receive physiotherapy treatment, participants will need to attend a 30-minute session, twice a week, for 12 weeks.<br>Device: Simulation of photobiomodulation<br>The simulation of PBM will use the same LED devices in the form of a shoulder brace but without active light. It will be applied following the same regimen of the Photobiomodulation group.

Outcome Measures

Primary Outcome Measure:

1. Quick-DASH functional scale
   In this project, the dysfunction and symptom component of the QuickDASH score, validated for Brazil and available online, will be used.

   The QuickDASH score is calculated as follows: At least 10 out of the 11 items must be answered. Each answered question will have a maximum value of 5. These values are then transformed into a score of 100 by subtracting 1 and multiplying by 25. This transformation is done to compare the scores with other scales ranging from 0 to 100. A high score indicates significant dysfunction.

   QuickDASH Score = [(Sum of responses / n) - 1] X 25 n: the number of questions answered.

   [Time Frame: The QuickDASH will be applied at 1 weeks after the surgical procedure.]
2. Quick-DASH functional scale
   In this project, the dysfunction and symptom component of the QuickDASH score, validated for Brazil and available online, will be used.

   The QuickDASH score is calculated as follows: At least 10 out of the 11 items must be answered. Each answered question will have a maximum value of 5. These values are then transformed into a score of 100 by subtracting 1 and multiplying by 25. This transformation is done to compare the scores with other scales ranging from 0 to 100. A high score indicates significant dysfunction.

   QuickDASH Score = [(Sum of responses / n) - 1] X 25 n: the number of questions answered.

   [Time Frame: The QuickDASH will be applied at 2 weeks after the surgical procedure.]
3. Quick-DASH functional scale
   In this project, the dysfunction and symptom component of the QuickDASH score, validated for Brazil and available online, will be used.

   The QuickDASH score is calculated as follows: At least 10 out of the 11 items must be answered. Each answered question will have a maximum value of 5. These values are then transformed into a score of 100 by subtracting 1 and multiplying by 25. This transformation is done to compare the scores with other scales ranging from 0 to 100. A high score indicates significant dysfunction.

   QuickDASH Score = [(Sum of responses / n) - 1] X 25 n: the number of questions answered.

   [Time Frame: The QuickDASH will be applied at 4 weeks after the surgical procedure.]
4. Quick-DASH functional scale
   In this project, the dysfunction and symptom component of the QuickDASH score, validated for Brazil and available online, will be used.

The QuickDASH score is calculated as follows: At least 10 out of the 11 items must be answered. Each answered question will have a maximum value of 5. These values are then transformed into a score of 100 by subtracting 1 and multiplying by 25. This transformation is done to compare the scores with other scales ranging from 0 to 100. A high score indicates significant dysfunction.

QuickDASH Score = [(Sum of responses / n) - 1] X 25 n: the number of questions answered.

[Time Frame: The QuickDASH will be applied at 8 weeks after the surgical procedure.]

5. Quick-DASH functional scale
   In this project, the dysfunction and symptom component of the QuickDASH score, validated for Brazil and available online, will be used.

   The QuickDASH score is calculated as follows: At least 10 out of the 11 items must be answered. Each answered question will have a maximum value of 5. These values are then transformed into a score of 100 by subtracting 1 and multiplying by 25. This transformation is done to compare the scores with other scales ranging from 0 to 100. A high score indicates significant dysfunction.

   QuickDASH Score = [(Sum of responses / n) - 1] X 25 n: the number of questions answered.

   [Time Frame: The QuickDASH will be applied at 12 weeks after the surgical procedure.]

Secondary Outcome Measure:

6. Range of Motion
   The passive range of motion of the shoulder on both sides will be assessed with the participant in an upright position. The maximum tolerated position for extension, flexion, abduction, adduction, external rotation, and internal rotation will be noted by the evaluator. Rotations will be evaluated at 0° of abduction and flexion. All measurements will be performed with a digital goniometer (Kaptron 360, Shenzhen, Dongguan China).

   [Time Frame: Range of motion will be assessed after 1 day of the surgical procedure]

7. Range of Motion
   The passive range of motion of the shoulder on both sides will be assessed with the participant in an upright position. The maximum tolerated position for extension, flexion, abduction, adduction, external rotation, and internal rotation will be noted by the evaluator. Rotations will be evaluated at 0° of abduction and flexion. All measurements will be performed with a digital goniometer (Kaptron 360, Shenzhen, Dongguan China).

   [Time Frame: Range of motion will be assessed after 1 week of the surgical procedure]

8. Range of Motion
   The passive range of motion of the shoulder on both sides will be assessed with the participant in an upright position. The maximum tolerated position for extension, flexion, abduction, adduction, external rotation, and internal rotation will be noted by the evaluator. Rotations will be evaluated at 0° of abduction and flexion. All measurements will be performed with a digital goniometer (Kaptron 360, Shenzhen, Dongguan China).

   [Time Frame: Range of motion will be assessed after 2 weeks of the surgical procedure]

9. Range of Motion
   The passive range of motion of the shoulder on both sides will be assessed with the participant in an upright position. The maximum tolerated position for extension, flexion, abduction, adduction, external rotation, and internal rotation will be noted by the evaluator. Rotations will be evaluated at 0° of abduction and flexion. All measurements will be performed with a digital goniometer (Kaptron 360, Shenzhen, Dongguan China).

   [Time Frame: Range of motion will be assessed after 4 weeks of the surgical procedure]

10. Range of Motion
    The passive range of motion of the shoulder on both sides will be assessed with the participant in an upright position. The maximum tolerated position for extension, flexion, abduction, adduction, external rotation, and internal rotation will be noted by the evaluator. Rotations will be evaluated at 0° of abduction and flexion. All measurements will be performed with a digital goniometer (Kaptron 360, Shenzhen, Dongguan China).

    [Time Frame: Range of motion will be assessed after 8 weeks of the surgical procedure]

11. Range of Motion
    The passive range of motion of the shoulder on both sides will be assessed with the participant in an upright position. The maximum tolerated position for extension, flexion, abduction, adduction, external rotation, and internal rotation

will be noted by the evaluator. Rotations will be evaluated at 0° of abduction and flexion. All measurements will be performed with a digital goniometer (Kaptron 360, Shenzhen, Dongguan China).

[Time Frame: Range of motion will be assessed after 12 weeks of the surgical procedure]

12. Muscle Strength
    Muscle strength will be assessed by progressive holding of dumbbells with elbow flexion (RLM, Maringá, Paraná, Brazil), starting with 500 grams and progressing to 5 kg in the 8th and 12th weeks.

    [Time Frame: 8th week of the surgical procedure]

13. Muscle Strength
    Muscle strength will be assessed by progressive holding of dumbbells with elbow flexion (RLM, Maringá, Paraná, Brazil), starting with 500 grams and progressing to 5 kg in the 8th and 12th weeks.

    [Time Frame: 12th week of the surgical procedure]

14. Pain Assessment
    The intensity of spontaneous pain and pain during movement of the injured limb will be assessed using the visual analog scale for pain. Pressure pain at the fracture site will be evaluated with a digital algometer (MED DOR, Governador Valadares, MG, Brazil)

    [Time Frame: It will be evaluated at 1 week of the surgical procedure]

15. Pain Assessment
    The intensity of spontaneous pain and pain during movement of the injured limb will be assessed using the visual analog scale for pain. Pressure pain at the fracture site will be evaluated with a digital algometer (MED DOR, Governador Valadares, MG, Brazil)

    [Time Frame: It will be evaluated at 2 weeks of the surgical procedure]

16. Pain Assessment
    The intensity of spontaneous pain and pain during movement of the injured limb will be assessed using the visual analog scale for pain. Pressure pain at the fracture site will be evaluated with a digital algometer (MED DOR, Governador Valadares, MG, Brazil)

    [Time Frame: It will be evaluated at 4 of the surgical procedure]

17. Pain Assessment
    The intensity of spontaneous pain and pain during movement of the injured limb will be assessed using the visual analog scale for pain. Pressure pain at the fracture site will be evaluated with a digital algometer (MED DOR, Governador Valadares, MG, Brazil)

    [Time Frame: It will be evaluated at 8 weeks of the surgical procedure]

18. Pain Assessment
    The intensity of spontaneous pain and pain during movement of the injured limb will be assessed using the visual analog scale for pain. Pressure pain at the fracture site will be evaluated with a digital algometer (MED DOR, Governador Valadares, MG, Brazil)

    [Time Frame: It will be evaluated at 12 weeks of the surgical procedure]

19. Analgesic Consumption
    The type and dosage of analgesics consumed will be inquired during the daily control of PBM use and will be recorded on the participant's daily control form.

    [Time Frame: It will be evaluated during a period of 12 weeks after surgical procedure]

20. SF-6 instrument
    Quality of life will be assessed using the SF-6 instrument from 2002 in the version adapted for use in Brazil at 1, 2, 4, 8, and 12 weeks, with the results recorded in the respective clinical records for each experimental period.

    [Time Frame: It will be evaluated at 1 week after surgical procedure]

21. SF-6 instrument
    Quality of life will be assessed using the SF-6 instrument from 2002 in the version adapted for use in Brazil at 1, 2, 4, 8, and 12 weeks, with the results recorded in the respective clinical records for each experimental period.

    [Time Frame: It will be evaluated at 2 weeks after surgical procedure]

22. SF-6 instrument
Quality of life will be assessed using the SF-6 instrument from 2002 in the version adapted for use in Brazil at 1, 2, 4, 8, and 12 weeks, with the results recorded in the respective clinical records for each experimental period.

[Time Frame: It will be evaluated at 4 weeks after surgical procedure]

23. SF-6 instrument
Quality of life will be assessed using the SF-6 instrument from 2002 in the version adapted for use in Brazil at 1, 2, 4, 8, and 12 weeks, with the results recorded in the respective clinical records for each experimental period.

[Time Frame: It will be evaluated at 8 weeks after surgical procedure]

24. SF-6 instrument
Quality of life will be assessed using the SF-6 instrument from 2002 in the version adapted for use in Brazil at 1, 2, 4, 8, and 12 weeks, with the results recorded in the respective clinical records for each experimental period.

[Time Frame: It will be evaluated at 12 weeks after surgical procedure]

25. Occurrence of Adverse Events
The occurrence of adverse events will be inquired during the daily control of PBM use and will be recorded on the participant's daily control form

[Time Frame: It will be evaluated during a period of 12 weeks after surgical procedure]

26. Fracture Healing
Shoulder X-rays in the anteroposterior, scapular profile, and axillary incidences will be taken after 4, 8, and 12 weeks to assess bone healing, defined by the presence of a bony callus in 3 of the 4 bone cortices joining the main fracture fragments and compared with the X-ray taken immediately post-surgery.

[Time Frame: It will be evaluated 4 weeks after surgical procedure]

27. Fracture Healing
Shoulder X-rays in the anteroposterior, scapular profile, and axillary incidences will be taken after 4, 8, and 12 weeks to assess bone healing, defined by the presence of a bony callus in 3 of the 4 bone cortices joining the main fracture fragments and compared with the X-ray taken immediately post-surgery.

[Time Frame: It will be evaluated 8 weeks after surgical procedure]

28. Fracture Healing
Shoulder X-rays in the anteroposterior, scapular profile, and axillary incidences will be taken after 4, 8, and 12 weeks to assess bone healing, defined by the presence of a bony callus in 3 of the 4 bone cortices joining the main fracture fragments and compared with the X-ray taken immediately post-surgery.

[Time Frame: It will be evaluated 12 weeks after surgical procedure]

29. Procedure-Related Costs
The costs of all procedures will be documented based on the Brazilian Unified Health System (SUS) price list

[Time Frame: It will be evaluated during a period of 12 weeks after surgical procedure]

Eligibility

Minimum Age: 18 Years

Maximum Age: 65 Years

Sex: All

Gender Based: No

Accepts Healthy Volunteers: Yes

Criteria: The subjects selected for inclusion will be individuals of both genders treated at HMACN hospital, with isolated and closed proximal humerus fractures with displacement and surgical indication, undergoing open reduction and internal fixation (ORIF) with a locking angular stability plate for the proximal humerus

(Neer 1070, Carrera et al., 2012; Petros, 2019), and meeting the following eligibility criteria.

Individuals aged between 18 and 65 years, of both genders, with proximal humerus fractures classified as:

Neer group III Neer group IV Neer group V AO/OTA subgroups A2 and A3 AO/OTA group B AO/OTA group C (only for patients under 55 years of age)

Exclusion criteria:

Individuals with pre-existing injuries or sequelae in the shoulder and shoulder girdle or motor deficits due to central or peripheral neurological injuries; Individuals with pathological fractures; Individuals who develop postoperative infection or implant loosening; Individuals with ipsilateral fractures in other regions of the limb; Individuals with neurovascular injuries causing sensory deficits at the injury site; Individuals with local or systemic conditions contraindicating surgical intervention or complicating postoperative recovery; Individuals with a history of photosensitivity; Individuals with neurological or psychiatric disorders; Individuals with proliferative or infectious skin lesions in the shoulder region receive LED light; Individuals who used anti-inflammatory drugs within five days before the trauma; Pregnant women; Individuals with surgical complications such as neurological or vascular injuries or fracture line extension during surgery will not meet the desired evolution pattern.

Contacts/Locations

Central Contact Person: Kristianne PS Fernandes, PhD
Telephone: +55 11 3385-9241
Email: kristianneporta@gmail.com

Central Contact Backup:

Study Officials: Kristianne PS Fernandes, PhD
Study Principal Investigator
University of Nove de Julho

Locations: **Brazil**
ACN Municipal Hospital
[Recruiting]
Sao Paulo, Brazil, 03807-230
Contact: Luiz Claudio Freitas, MD +55(11) 3394-8030

IPDSharing

Plan to Share IPD: Yes
Collected data will be stored, and organized in a repository, and appropriate statistical tests will be applied to specific analyses

Supporting Information:
Study Protocol

Time Frame:
Data will be shared at the end of the study

Access Criteria:
Data will be shared at the end of the study for everyone who requests it

URL:

References

Citations:

Links:

Available IPD/Information:

U.S. National Library of Medicine | U.S. National Institutes of Health | U.S. Department of Health & Human Services
